# Identification of a NUAK1/2 Inhibitor as a Macrophage-Polarizing Compound by a Machine Learning-Based Phenotypic Cell Painting Screen

**DOI:** 10.3390/ijms27167279

**Published:** 2026-08-14

**Authors:** Johanna B. Brüggenthies-Brunner, Jakob Dittmer, Julia Sauer, Sarah Groetzner, Anika Liu, Eva Martin, Anja Teufel, Christine Strasser, Helga Bronner, Robert Ries, Marc A. Grundl, Lina Humbeck, Michael Schuler, Bernd Weigle

**Affiliations:** 1 Eye Health & Research Beyond Borders, Boehringer Ingelheim Pharma GmbH & Co. KG, 88397 Biberach a.d. Riss, Germany; johanna_barbara.brueggenthies-brunner@boehringer-ingelheim.com (J.B.B.-B.); anja.teufel@boehringer-ingelheim.com (A.T.); christine.strasser@boehringer-ingelheim.com (C.S.); 2 Boehringer Ingelheim RCV GmbH & Co. KG, 1121 Vienna, Austria; jakob.dittmer@boehringer-ingelheim.com; 3 Immunology & Respiratory, Boehringer Ingelheim Pharma GmbH & Co. KG, 88397 Biberach a.d. Riss, Germany; julia.sauer@boehringer-ingelheim.com (J.S.); sarah.groetzner@boehringer-ingelheim.com (S.G.); 4 Global Computational Innovation, Boehringer Ingelheim Pharma GmbH & Co. KG, 88397 Biberach a.d. Riss, Germany; anika.liu@boehringer-ingelheim.com; 5 Global Drug Discovery Sciences, Boehringer Ingelheim Pharma GmbH & Co. KG, 88397 Biberach a.d. Riss, Germany; eva.martin@boehringer-ingelheim.com (E.M.); helga.bronner@boehringer-ingelheim.com (H.B.); robert.ries@boerhinger-ingelheim.com (R.R.); michael.schuler@boehringer-ingelheim.com (M.S.); 6 Global Medicinal Chemistry, Boehringer Ingelheim Pharma GmbH & Co. KG, 88397 Biberach a.d. Riss, Germany; marc.grundl@boehringer-ingelheim.com (M.A.G.); lina.humbeck@boehringer-ingelheim.com (L.H.)

**Keywords:** macrophage polarization, macrophage states, small molecules, cell painting, human iPSC-derived and monocyte-derived macrophages, phenotypic high-content screen, machine learning-based image analysis, NUAK1/2 inhibition, phagocytosis/efferocytosis, ferroptosis

## Abstract

Disease-associated macrophage states contribute to the pathogenesis of numerous inflammatory disorders. While small molecule-mediated macrophage repolarization represents a promising strategy to restore homeostasis, current approaches often rely on predefined molecular markers and may therefore overlook previously unrecognized modulators of macrophage plasticity. To address this limitation, we performed a phenotypic high-content cell painting screen in induced pluripotent stem cell-derived and blood monocyte-derived macrophages. Using our previously established machine learning-based cell painting analysis pipeline, we screened the annotated opnMe and JUMP-CP compound libraries and identified 26 compounds that induced M1- or M2-like polarization phenotypes. Among these hits, we identified WZ4003, an inhibitor of AMPK-related kinases NUAK1 and NUAK2, as novel macrophage-polarizing compound. Combining cell painting feature profiling with functional analyses, we revealed that the NUAK1/2 inhibitor WZ4003 induces a distinct macrophage state characterized by a rounded M1-like morphology, ferroptosis protection, mitochondrial reactive oxygen species increase, antioxidative adaptation, and phagocytosis and efferocytosis impairment. These findings link specific morphological signatures to functional macrophage states. Overall, this study provides a resource of macrophage-polarizing compounds and demonstrates the utility of machine learning-based cell painting for identifying novel modulators of macrophage polarization states.

## 1. Introduction

Disease-associated macrophage (MΦ) states are implicated in a broad range of inflammation-driven pathologies, including cancer, fibrosis or metabolic disorders [1,2,3,4]. MΦ polarization critically regulates key functions of host defense, immune regulation, tissue homeostasis and repair processes [5]. Repolarization of disease-associated MΦs toward homeostatic states using small-molecule compounds (cpds) represents a promising strategy in inflammatory disorders [6,7]. Consequently, the identification of cpds capable of modulating MΦ polarization as well as their underlying molecular targets is of considerable interest for early-stage drug discovery [8,9,10,11].

MΦ polarization is commonly characterized by using predefined sets of molecular markers [12]. However, such approaches only partially capture the heterogeneity and functional diversity of MΦ states beyond the simplified pro-inflammatory M1 and anti-inflammatory M2 framework [13,14,15,16,17]. Previous studies indicate that MΦ polarization states are associated with distinct morphological programs. In both murine and human MΦs, cellular shape has been shown to provide a quantitative phenotypic readout that reflects polarization status. While pro-inflammatory polarization is associated with rounded and flattened morphologies, anti-inflammatory states typically exhibit a more elongated shape [8,18,19].

Cell painting is a high-content imaging approach that enables systematic and unbiased characterization of cellular morphology [20]. By combining multiplexed fluorescent staining of major cellular compartments and organelles (DNA, cytoplasmic RNA, nuclei, actin filaments, Golgi apparatus, plasma membrane, ER, and mitochondria), with automated image analysis, thousands of quantitative features describing cell shape, texture, intensity, and intracellular organization can be extracted from individual cells [20,21]. In recent years, cell painting has emerged as a valuable tool for phenotypic profiling, mechanism of action (MoA) studies, and cpd screening across diverse biological systems [22].

While MΦ morphology has been linked to polarization state and cell painting has emerged as a comprehensive platform for unbiased cellular profiling, it remains unclear whether morphology-based phenotypic signatures can be systematically leveraged to identify novel small-molecule modulators of MΦ polarization and to resolve functionally distinct MΦ states. To address this critical knowledge gap, we hypothesized that machine learning (ML)-guided cell painting can bridge this gap [11].

Building on our previous work [11] establishing morphology-based readouts using reference MΦ-polarizing cpds (named M1-like or M2-like modulators [8]), we implemented an automated high-content platform that combines MΦ cell painting with ML-based image analysis. This approach enables quantitative assessment of cpd-induced MΦ (re-)polarizing effects across human induced pluripotent stem cell (iPSC)-derived MΦ s (IDMs) and monocyte-derived MΦs (MDMs). In this context, we optimized the generation of IDMs, which provide a scalable and experimentally accessible alternative to human blood donor-derived MDMs for phenotypic screening applications [12,23,24,25,26,27,28,29,30,31,32,33,34,35,36]. In addition, the ML-based cell painting platform can resolve subtle phenotypic cellular states across the MΦ polarization spectrum, thereby capturing the complexity of MΦ plasticity beyond the traditional M1/M2 dichotomy. Using this platform, we here present a follow-up study in which we screened 540 annotated cpds from two publicly available small molecule libraries (Boehringer-Ingelheim’s opnMe initiative (opnme.com) and the Joint Undertaking for Morphological Profiling-Cell Painting (JUMP-CP) consortium) to identify novel MΦ-polarizing cpds. To assess the reproducibility of identified phenotypes and robustness of hits across MΦ models, cpds were screened in IDMs and MDMs from four independent blood donors. Cpds inducing comparable M1- or M2-like polarization signatures in IDMs as well as in MDMs from at least 3 donors were subsequently evaluated in a hit confirmation screen and profiled in a dose–response analysis for cpd-induced cell toxicity assessment.

Out of the 26 identified and validated MΦ-polarizing cpd hits, we focused on one hit, WZ4003, a dual kinase novel (nua) kinases 1 and 2 (NUAK1/2) inhibitor from the JUMP-CP library for functional characterization. NUAK1 and NUAK2 belong to the family of AMP-activated protein kinase (AMPK)-related kinases (ARKs) and share substantial sequence homology with each other and with the catalytic subunit of AMPK [37,38]. In contrast to AMPK, which functions as a central cellular energy sensor, NUAK1/2 have been implicated in the regulation of diverse metabolic and signaling pathways involved in cellular homeostasis [37,38]. Active AMPK is known to promote anti-inflammatory MΦ polarization [39,40], while evidence linking NUAK1 and NUAK2 to MΦ polarization is scarce.

Subsequently, we investigated the in vitro effects of pharmacological NUAK1/2 inhibition by WZ4003 and related chemical derivatives (HTH-01-015, HTH-02-006) in polarized and reprogrammed MΦs. Thereby, pharmacological inhibition of NUAK1/2 was associated with a distinct polarization signature characterized by impaired phagocytosis/efferocytosis, mitochondrial reactive oxygen species (ROS) accumulation, adaptive antioxidant responses, and protection from RSL3-induced ferroptosis. Furthermore, our mitochondrial cell painting analysis indicated that pharmacological NUAK1/2 inhibition exhibited a feature profile highly reminiscent of that induced by the electron transport chain inhibitors rotenone and antimycin A (Rot/AA).

Taken together, our study highlights the utility of ML-based phenotypic cell painting screens as an approach for identifying modulators of MΦ polarization and explores a previously unrecognized role of pharmacological NUAK1/2 inhibition in inducing a functionally distinct MΦ polarization state.

## 2. Results

### 2.1. AMPK-Related Kinase Modulators (WZ4003 and BI-9774) Are Identified as Novel MΦ-Polarizing cpds in a Phenotypic Cell Painting Screen

**Figure 1:**
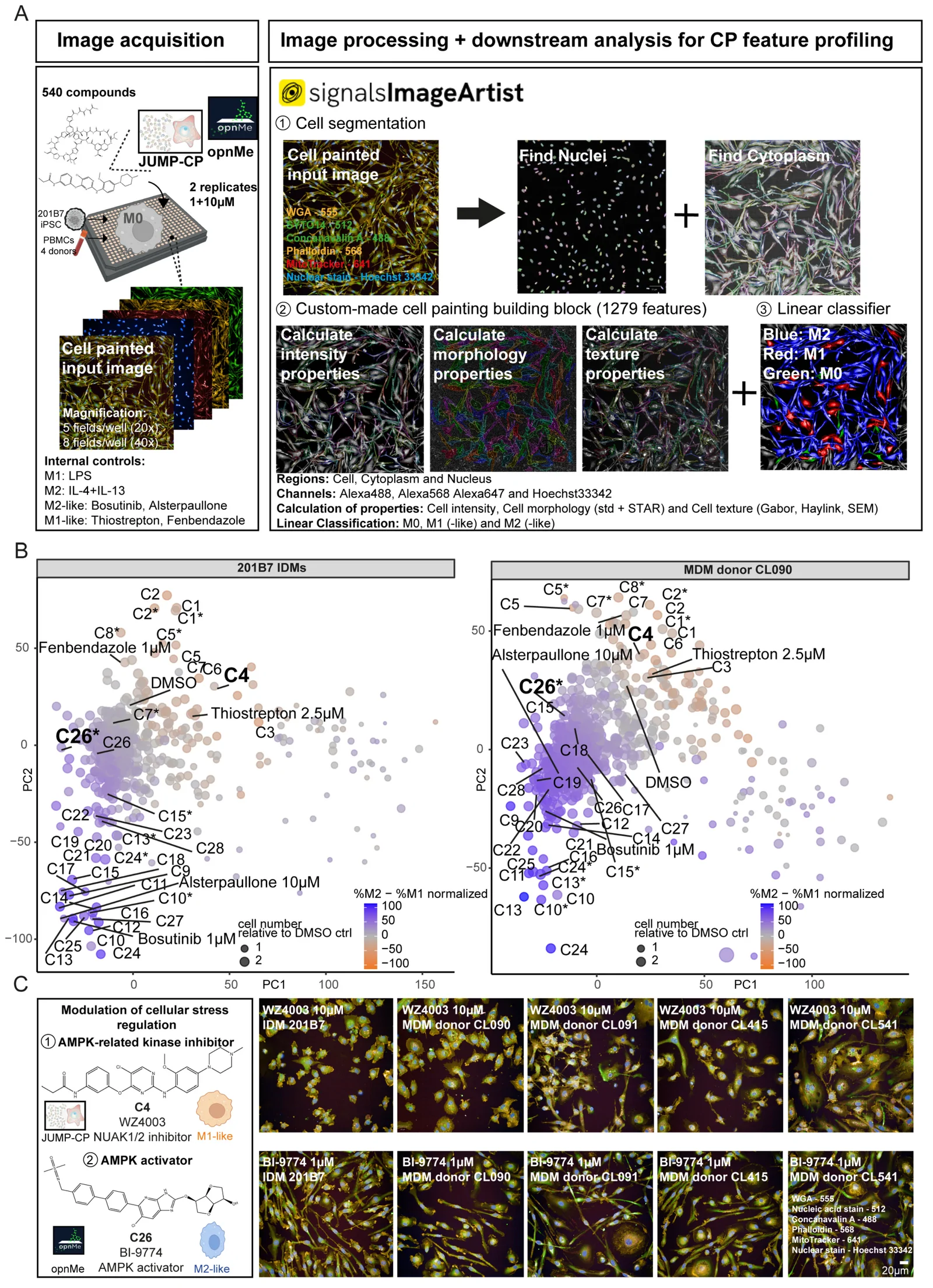
Identification of AMPK-related kinase modulators (WZ4003 and BI-9774) as novel MΦ-polarizing cpds in a phenotypic cell painting screen. (**A**) Schematic overview of the cell painting (CP) feature profiling workflow using Signals ImageArtist (SImA) (created with Adobe Illustrator), as adapted from [8]. Cell painting images (5 fields per well at 20× magnification; 8 fields per well at 40× magnification) from cpds-treated (JUMP-CP and opnMe libraries; controls: thiostrepton, fenbendazole, bosutinib, alsterpaullone) or biologically (LPS or IL-4 + IL-13) stimulated iPSC- and monocyte-derived MΦs (unpolarized M0 state) were acquired using the Opera Phenix™ confocal imaging system. This was followed by cell segmentation and the extraction of 1279 distinct cell painting features encompassing morphology, texture, and intensity properties across all cellular regions and channels, combined with a linear classification of M0, M1-like, and M2-like polarization states. Representative cell images illustrate bosutinib (1 µM)-treated IDMs at the 24 h time point. The phenotypic screen and the downstream image analysis pipeline are described in detail in the Materials and Methods section. (**B**) Principal component analysis (PCA) of the primary screen hits determined by cell painting feature profiling. MΦs were treated for 24 h with the illustrated conditions (540 cpds across the JUMP-CP and opnMe libraries; individual hit cpds are annotated as C1–C28; see Tables S1 and S2; asterisks [*] denote a treatment concentration of 1 µM, all other cpd concentrations are 10 µM unless specified otherwise), alongside internal M1- and M2-like control cpds (bosutinib 1 µM, alsterpaullone 10 µM, thiostrepton 2.5 µM, and fenbendazole 1 µM). DMSO-normalized %M2 − %M1 values represent the mean of two screen replicates (*n* = 2 with 5 fields imaged per well at 20× magnification) from 384-well plates, depicted for IDMs (201B7 line) and a representative MDM donor (CL090). Other control conditions consisted of multiple wells per plate with 5 fields imaged per well, replicated across 384-well plates (*n* = 24 wells per condition). The absolute cell count relative to the vehicle (DMSO) control is represented proportionally by the diameter of the respective data points. The corresponding DMSO-normalized %M2 − %M1 source values are provided in Table S3. PC1 and PC2 capture the highest variance of the extracted features. (**C**) Schematic and representative cell painting images of two identified primary hits. Representative high-content Opera Phenix™ confocal images (40× magnification; one representative field shown) of cell-painted IDMs and MDMs (donors CL091, 090, 541, and 415) treated for 24 h with cpd C26 (opnMe; AMPK activator; BI-9774) and cpd C4 (JUMP-CP; NUAK1/2 inhibitor; WZ4003) at the indicated concentrations. Cell painting was performed using the PhenoVue™ Kit components (specifically: PhenoVue™ 641 Mitochondrial Stain, PhenoVue™ Hoechst 33342 Nuclear Stain, PhenoVue™ Fluor 488-Concanavalin A, PhenoVue™ 512 Nucleic Acid Stain, PhenoVue™ Fluor 555-WGA, and PhenoVue™ Fluor 568-Phalloidin). The schematic was created with Adobe Illustrator, BioRender.com, and ChemDraw (version 26.0).

In this study, we performed a phenotypic cell painting high-content screen of 540 cpds at 1 µM and 10 µM from the two annotated publicly available libraries (opnMe: 144 cpds; partial JUMP-CP: 396 cpds; Table S1) to identify novel MΦ-polarizing cpds (Figure 1A). To ensure hit robustness across MΦ models, we used unpolarized (M0) IDMs and primary MDMs from four donors, which were already quality controlled and characterized in our previous study [11]. Following a 24 h treatment, cells were painted to visualize nine cellular compartments (mitochondria, nucleus, RNA, nucleoli, Golgi apparatus, cytoplasm, ER, actin and plasma membrane) and imaged via a high-content confocal Opera Phenix™ microscope. Biological references (LPS, IL-4 + IL-13) and M1-/M2-like control cpds (fenbendazole, thiostrepton, bosutinib, and alsterpaullone) were included as internal standards and the terminology M1-like and M2-like for cpd-induced MΦ polarization changes indicated by morphological cell shape changes was adapted from [8]. For image processing and downstream cell painting feature profiling, we first segmented the cells by the nucleus stain (Hoechst33342 channel) and defined the cell surroundings by the cytoplasm/ER concanavalin A stain (Alexa488 channel). Images were processed using a customized cell painting image analysis pipeline within Signals ImageArtist (SImA) to extract 1279 cell painting features (morphology, texture, and intensity properties) across several regions (cell, cytoplasm, and nucleus) of each channel (Alexa488, 647, 568, and Hoechst33342). This analysis was combined with an IDM-trained linear classification algorithm to quantify MΦ polarization states (M0, M1-like and M2-like) (Figure 1A).

We evaluated cpd-induced cell-state changes using a DMSO-normalized cell roundness Z-score, where Z-score > 0 indicates rounded (M1-like) and Z-score < 0 indicates elongated (M2-like) shapes. Reference cpds showed consistent cell shape changes across both MΦ models (Figure S1A). Based on control-defined thresholds, we identified 20 elongated hits and 8 rounded hits consistently across both MΦ models (Figure S1A and Table S2). We noted cell shape heterogeneity across primary MDM donors (Figure S1A; e.g., donor CL541), reflecting expected inter-donor variance and phenotypic heterogeneity of primary cells [41]. To confirm the 28 hits, we performed the cell painting feature analysis as an additional orthologous phenotypic read-out (Figure 1B and Figure S1B). We quantified for each cpd the DMSO-normalized %M2 − %M1 value and the cell number to assess cell cytotoxicity. The identified 28 hits showed consistent %M2 − %M1 shifts across the cell models (Figure 1B and Figure S1B and Table S3). Again, inter-donor effects became apparent, as represented in the PCA analyses by variation of absolute values or signature distribution across the MDM donors (Figure 1B and Figure S1B; e.g., donor CL541).

The hit list (Table S2) contained several cpds known to impact MΦ polarization, validating our approach: (1) Known MΦ polarizers, such as arcyriaflavin A (C10) and SU11274 (C17) [8]. (2) Cpds targeting pathways involved in MΦ regulation (MAPK, JAK-STAT, PI3K/AKT [6]): the JAK1/2 inhibitor ruxolitinib (C12), a JNK inhibitor (C13), three p38 MAPK inhibitors (C23, C25, and C28), a pan-RSK inhibitor (C24), and a c-Raf inhibitor (C14) all scored as M2-like. Conversely, the pan-PI3K inhibitor buparlisib (C6) and two microtubule stabilizers/αβ-tubulin binders (C1 [42], C2) were identified as M1-like. (3) Structurally similar cpds, like oxibendazole (C8), which showed a similar M1-like profile to its derivative control, fenbendazole [43].

Intriguingly, the hit list highlighted metabolic regulators, specifically two cpds addressing the ARK family with distinct MoA and opposing phenotypic changes: the pan-AMPK activator BI-9774 (opnMe; C26; [44]) induced an elongated phenotype, whereas the NUAK1/2 inhibitor WZ4003 (JUMP-CP; C4) induced a rounded shape (Figure 1C and Figure S1A). Cell painting quantification revealed the modest induction of 13.4% more M1-like cells upon NUAK1/2 inhibition (WZ4003, 10 µM), compared to 31.2% more M2-like cells upon AMPK activation (BI-9774, 1 µM) in IDMs (Table S3 and Figure S1B).

In summary, our phenotypic screen identified 20 M2-like and 8 M1-like cpds modulating MΦ polarization in both IDMs and MDMs. Strikingly, our data indicate that WZ4003 (NUAK1/2 inhibitor) acts as a novel M1-like-polarizing cpd, while activation of AMPK—the prototypical ARK—by BI-9774 exerts the opposite phenotypic effect.

### 2.2. WZ4003- and BI-9774-Induced MΦ Polarization Is Characterized by Cell Shape Changes and Impacts MΦs Efferocytic Capacity

**Figure 2:**
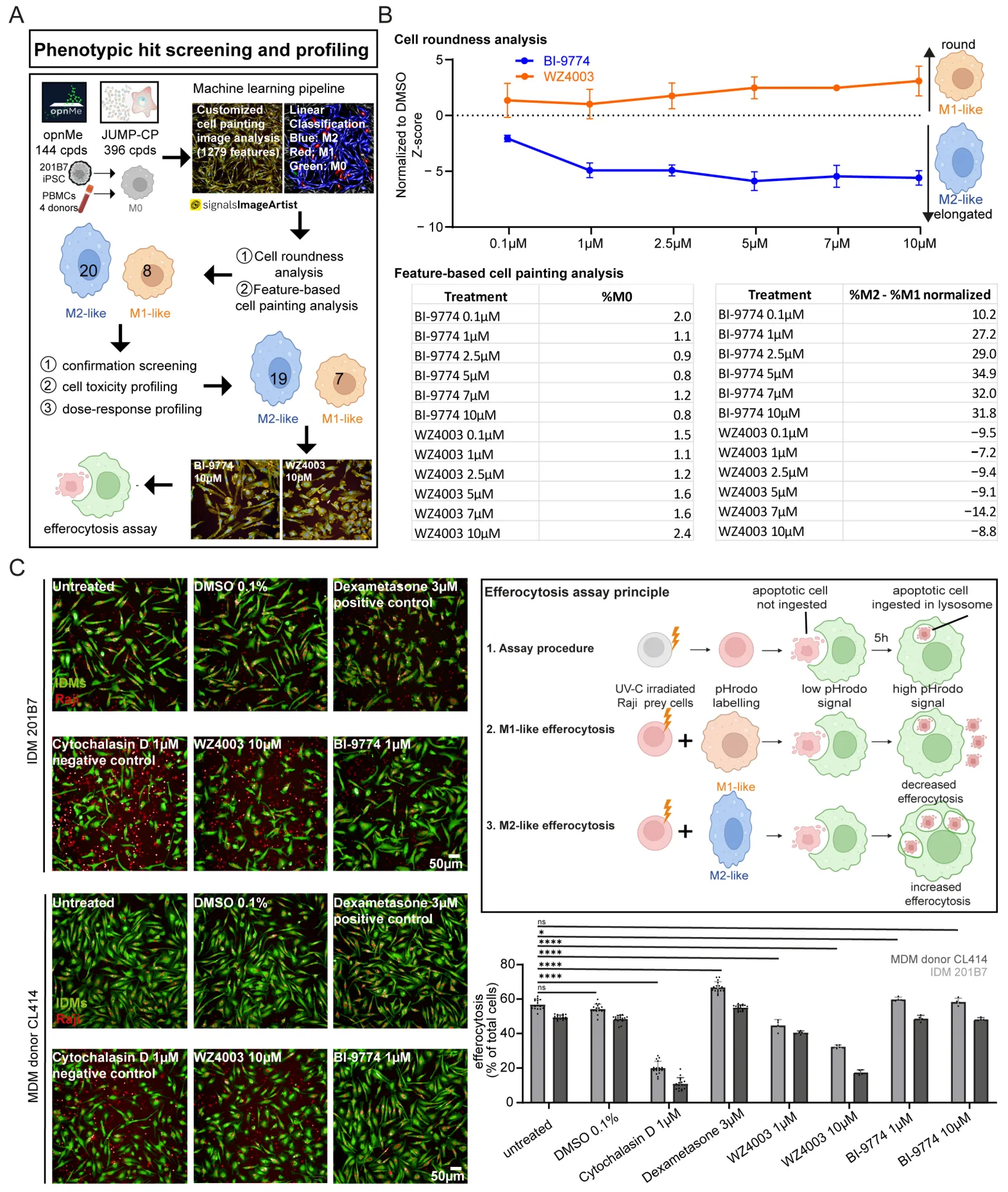
WZ4003- and BI-9774-induced MΦ polarization modulates cellular shape and efferocytic function. (**A**) Schematic overview of the phenotypic cell painting screening and profiling validation cascade: hit identification and validation (created with Adobe Illustrator). In total, 26 cpds out of 540 cpds from the opnMe (144 cpds) and JUMP-CP (partially; 396 cpds) libraries were identified as robust hits crossing the selection thresholds across MΦ models (IDM 201B7 line and four MDM donors). This was achieved using primary phenotypic screen read-outs (cell roundness and feature-based cell painting analysis) followed by downstream profiling via a hit confirmation screen, dose–response assessments, and cellular cytotoxicity validation. The two hits, BI-9774 and WZ4003, were functionally characterized using an efferocytosis assay. (**B**) Dose-dependent Z-score calculation for cell roundness analysis of WZ4003- and BI-9774-treated IDMs. DMSO-normalized Z-score values were determined from 20× magnified images and are depicted as mean ± SD for IDMs (201B7 line). Cells were treated with the illustrated conditions (WZ4003 and BI-9774) for 24 h at six different concentrations (0.1 µM, 1 µM, 2.5 µM, 5 µM, 7 µM, and 10 µM). Data points represent the mean ± SD calculated across two screen replicates (each consisting of 2 wells per plate, with 5 fields imaged per well, totaling *n* = 4 wells per condition). Corresponding cell painting analysis values for DMSO-normalized %M2 − %M1 and %M0 populations are shown in the accompanying table. (**C**) Efferocytosis of WZ4003- and BI-9774-treated MΦs. The schematic (created with BioRender.com and Adobe Illustrator) indicates the experimental principle: CellTracker™ Green CMFDA dye-labeled IDMs (201B7 line) and MDMs (donor CL414) were treated for 24 h with WZ4003 or BI-9774, followed by the addition of UV-irradiated, pHrodo Red-labeled apoptotic Raji cells. Measurements were taken after 5 h co-cultivation using the Opera Phenix™ imaging system (5 fields per well at 20× magnification). Data are depicted as the percentage of total MΦs that engulfed apoptotic Raji cells (*n* = 4 replicates per cpd concentration; *n* = 16 replicates for vehicle/positive/negative controls). Representative high-content Opera Phenix™ confocal images (40× magnification; one representative field shown) illustrate the engulfed apoptotic Raji cells (red) within cpd-treated MΦs (green). Cytochalasin D (1 µM, 1 h pre-treatment) and dexamethasone (3 µM, 24 h treatment) served as internal negative and positive controls, respectively. Corresponding cell numbers can be found in Table S5. Statistics: Data were analyzed using two-way ANOVA followed by Dunnett’s multiple comparisons test against the vehicle (DMSO 0.1%) control; asterisks denote statistical significance (**** *p* < 0.0001, * *p* < 0.05, ns = non-significant).

Overall, the primary phenotypic screen initially identified 28 MΦ-polarizing cpds (Table S2). To confirm these hits, we performed a hit confirmation screen using a different batch of IDMs and MDMs from a new donor (CL414), followed by dose–response and cytotoxicity profiling of the 28 selected hits (Figure 2A). To link phenotypes to function, the hit cpds of greatest interest—the NUAK1/2 inhibitor WZ4003 and the AMPK activator BI-9774—were functionally characterized using an efferocytosis assay.

Cell roundness analysis and feature-based cell painting yielded effect sizes similar to the primary screen (Table S4 and Figure S2A,B). Most cpds showed coupled, dose-dependent changes in both cell shape and polarization state (Figure S2C,D). In contrast, the microtubule stabilizers (C1 and C2) did not show a dose-dependent roundness trend, maintaining a high M1-like population even at lower doses. Moreover, the GSK-3 inhibitor (C27) and delanzomib (C5) induced dose-dependent cytotoxicity (Figure S2C,D). Consequently, we excluded C5 and C27, refining our final selection of hits.

In alignment with our primary data, WZ4003 (C4) induced a shift toward rounded cell shapes in both IDMs and MDMs (Figure S2A). Conversely, AMPK activation via BI-9774 (C26) was associated with an elongated phenotype in IDMs. Although a similar trend was seen for WZ4003 in MDMs (donor CL414), it did not reach it for BI-9774 (Figure S2A). This aligns with our cell painting feature analysis, where WZ4003 shifted both MΦ models toward an M1-like state, whereas BI-9774 drove an M2-like shift exclusively in IDMs (Figure S2B and Table S4). This outcome underscores the phenotypic heterogeneity of donor-derived MDMs, suggesting that IDMs can serve as a valuable, reproducible screening model in phenotypic drug discovery [12,23,24,25,26,27,28,29,30,31,32,33,34,35,36]. Therefore, follow-up experiments were conducted exclusively in IDMs. Dose–response assays (0.1–10 µM) in IDMs confirmed that both cpds dose-dependently induced the expected morphological and polarization shifts (Figure 2B and Figure S2C,D). At 10 µM, BI-9774 increased the M2-like population by 31.8% relative to the DMSO control, while WZ4003 expanded the M1-like population by 8.8% over untreated cells (Figure 2B).

To link phenotypic effects of these cpds to efferocytosis, a hallmark of MΦ function, we tracked the engulfment of pHrodo Red-labeled apoptotic Raji cells by green-labeled MΦs (Figure 2C). Cytochalasin D and dexamethasone served as negative control for apoptotic cell engulfment and positive control for clearance activation, respectively. Cell viability remained stable across all conditions (Table S5). WZ4003 treatment reduced the engulfment of apoptotic Raji cells after 5 h of co-cultivation in a dose-dependent manner, resulting in a 1.7-fold reduction in IDMs and a 2.8-fold reduction in MDMs (Figure 2C). In contrast, BI-9774 treatment modestly enhanced efferocytosis (by 5%) at both 1 µM and 10 µM in IDMs (Figure 2C). This opposing yet congruent functional behavior matches the opposing morphological phenotypes induced by the two cpds.

In summary, our validation cascade refined the initial hit list to 26 robust cpds by screening alternative cell batches and filtering out cytotoxic artifacts (C5, C27). Crucially, these phenotypic shifts correlated with distinct functional outcomes: AMPK activation (BI-9774) promoted an elongated M2-like phenotype that sustained or enhanced efferocytosis, whereas NUAK1/2 inhibition (WZ4003) drove a rounded M1-like phenotype that impaired apoptotic cell clearance.

### 2.3. MΦ Reprogramming by NUAK1/2 Inhibition Reduces the Phagocytic Capacity

Active AMPK is known to guide metabolic programming toward OXPHOS and promote anti-inflammatory MΦ polarization [39,40]. While evidence linking NUAK1 and NUAK2 to MΦ polarization is scarce, their roles in metabolic stress and signaling (e.g., NUAK1: AKT/mTOR signaling in cancer, NUAK1/2: TGF-β signaling in fibrosis) [45,46,47,48,49,50] suggest biological relevance. Therefore, we aimed to better understand the (re-)polarizing capacity of WZ4003-induced NUAK1/2 inhibition on a phenotypic and functional level.

**Figure 3:**
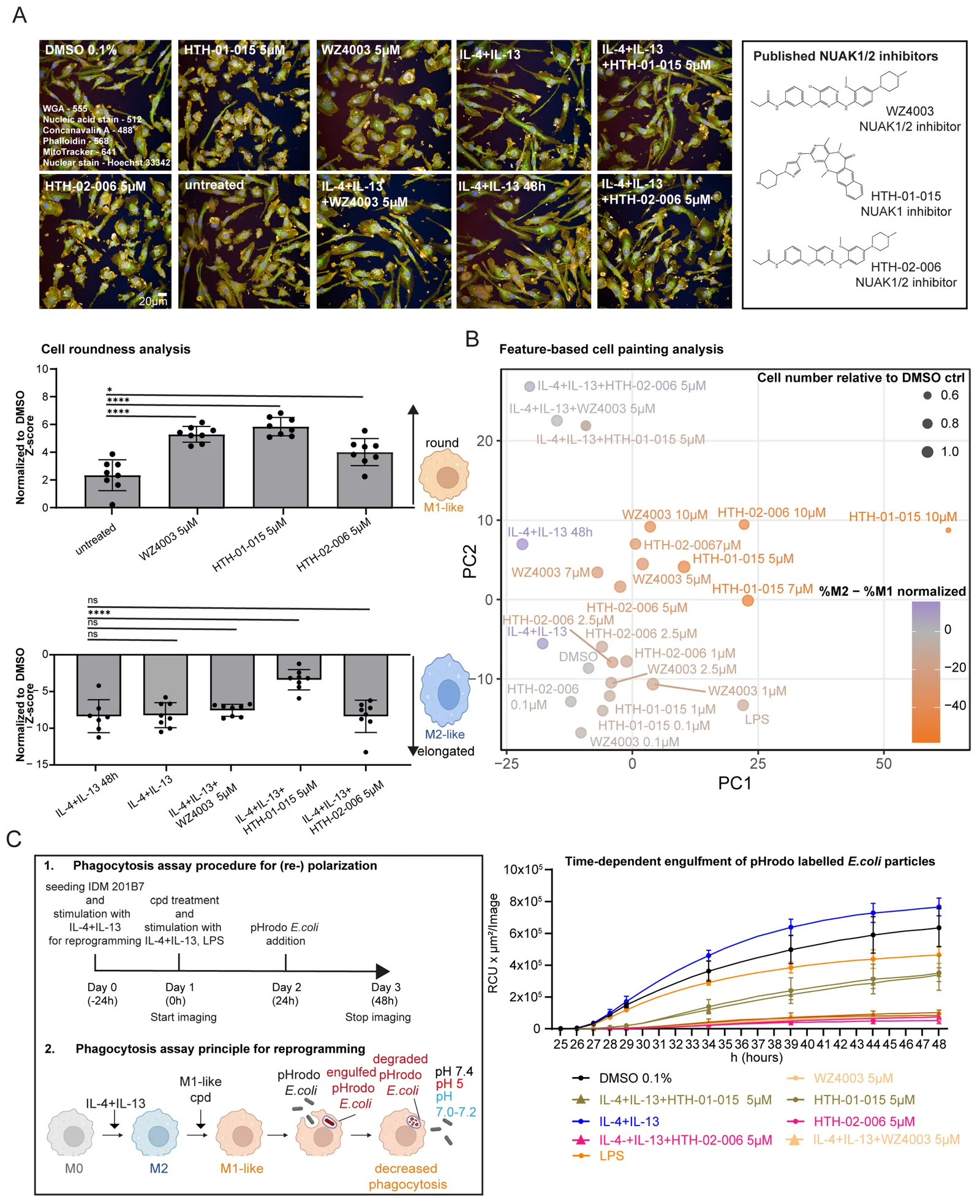
Reprogramming of NUAK1/2-inhibited MΦs reduces their phagocytic capacity. (**A**) Schematic overview and Z-score calculation for cell roundness analysis of NUAK1/2-inhibited IDMs. DMSO-normalized Z-score values were determined from 20× magnified images and are depicted as mean ± SD for IDMs (201B7 line). Cells were treated with the indicated NUAK1/NUAK2 inhibitors (WZ4003, HTH-01-015, and HTH-02-006) or biological stimuli (IL-4 + IL-13, 20 ng/mL) for 24 h or 48 h. In the case of MΦ repolarization, cells were pre-treated for 24 h with IL-4 + IL-13 (20 ng/mL), followed by a subsequent 24 h co-treatment with the respective cpds. Data points represent the mean ± SD calculated across two screen replicates (each consisting of 4 wells per plate, with 5 fields imaged per well, totaling *n* = 8 wells per condition). Representative high-content Opera Phenix™ confocal images (40× magnification; one representative field shown) illustrate cell-painted and polarized or repolarized IDMs. Cell painting was performed using PhenoVue™ Kit components (specifically: PhenoVue™ 641 Mitochondrial Stain, PhenoVue™ Hoechst 33342 Nuclear Stain, PhenoVue™ Fluor 488-Concanavalin A, PhenoVue™ 512 Nucleic Acid Stain, PhenoVue™ Fluor 555-WGA, and PhenoVue™ Fluor 568-Phalloidin). The schematic indicates the chemical structures of the three publicly available NUAK1 and/or NUAK2 inhibitors used in this study (created with BioRender.com, Adobe Illustrator, and ChemDraw). Statistics: Data were analyzed using one-way ANOVA followed by Dunnett’s multiple comparisons test against the respective control group; asterisks denote statistical significance (**** *p* < 0.0001, * *p* < 0.05, ns = non-significant). (**B**) Principal component analysis (PCA) of cell-painted profiles of NUAK1/2-inhibited IDMs. IDMs were treated dose-dependently with the conditions described in A. DMSO-normalized %M2 − %M1 values represent the mean calculated across two replicates (each consisting of 2 wells per plate, totaling *n* = 4 wells per condition, with 5 fields imaged per well at 20× magnification). The absolute cell count relative to the vehicle (DMSO) control is represented proportionally by the diameter of the data points. (**C**) Schematic and time-course analysis of *E. coli* phagocytosis in NUAK1/2-inhibited IDMs. IDMs were treated as described in A (*n* = 3 wells per condition). Data are depicted as total red object integrated intensity (RCU × µm^2^/image) reflecting the time-dependent engulfment of pHrodo Red-labeled *E. coli* particles, monitored using an IncuCyte^®^ SX3 live-cell imaging system (from 0 h to 24 h post-addition; imaged every hour at 10× magnification). The schematic illustrates the experimental timeline for cpd and *E. coli* addition, alongside the general pH-dependent fluorescence principle of the pHrodo Red assay. For clarity, error bars are omitted and statistics are in Figure S3F.

To strengthen confidence in our findings, we incorporated two additional publicly available NUAK1/2 inhibitors into our study: HTH-01-015 (NUAK1 IC_50_ 100 nM; NUAK2 IC_50_ > 10,000 nM in biochemical assays) and HTH-02-006 (NUAK1: >99% inhibition at 1000 nM; NUAK2 IC_50_ 126 nM in biochemical assays) [51,52] (Figure 3A). Despite the higher potency of HTH-01-015 for NUAK1, the micromolar concentrations used in our assays suggest that both kinases, NUAK1 and NUAK2, were inhibited [51,52]. Therefore, we decided to use for all three cpds (HTH-01-015, WZ4003, and HTH-02-006) the terminology NUAK1/2 inhibition or NUAK1/2i to indicate pharmacological inhibition of both kinases. IDMs treated with HTH-01-015 or HTH-02-006 exhibited a dose-dependent M1-like phenotypic shift (increased roundness and %M1-like population) that mirrored the effects of WZ4003 (Figure 3A,B and Figure S3A,B). Based on toxicity profiles and literature, 5 µM was selected as the standard experimental dose.

Immunoblotting revealed that IL-4 + IL-13 stimulation upregulated both NUAK1 and NUAK2 protein expression (Figure S3C). We next evaluated the repolarization capacity of the three inhibitors by stimulating MΦs with an anti-inflammatory M2 stimulus (IL-4 + IL-13) for 24 h before cpd co-treatment, mimicking physiological conditions [8]. Morphological profiling of these reprogrammed cells showed an expanded M1-like population, though a prominent M2 -like population persisted (Figure 3A,B and Figure S3A,B). This heterogeneous morphology and incomplete shift are likely driven by the continuous presence of unwashed IL-4 + IL-13 during the assay. Notably, no cellular toxicity was observed under this setup (Figure S3D,E).

To assess functional repolarization, we tracked the engulfment of pHrodo Red-labeled *E. coli* particles using live-cell imaging. Corroborating our earlier efferocytosis data (Figure 2C), NUAK1/2 inhibition suppressed the phagocytic capacity of IDMs over time during both initial polarization and subsequent repolarization (Figure 3C and Figure S3F). To exclude lineage-specific artifacts, this assay was validated in ChiPSC12-derived MΦs, which yielded identical results to the primary 201B7 line (Figure S3G). To determine if this functional defect was reversible, we washed out the IL-4 + IL-13 stimulus and cpds by washing three times with PBS, replenished the cells with fresh medium, and monitored phagocytosis kinetics for 24 h. Upon cpd removal, WZ4003-polarized or repolarized IDMs recovered their engulfment capacity, matching 48 h non-treated controls (Figure S3H). Crucially, introducing WZ4003 after washing out the IL-4 + IL-13 stimulus still suppressed phagocytosis, suggesting that NUAK1/2i-mediated functional restriction can operate independently of active IL-4 + IL-13 signaling (Figure S3H; named: IL-4 + IL-13 + wash + WZ4003).

To evaluate general relevance, we extended our workflow to iPSC-derived microglia, where research links NUAK1 to axonal mitochondrial trafficking and WZ4003 to the regulation of tau hyperphosphorylation (Ser365) in Alzheimer’s disease and primary tauopathies [53,54]. After confirming baseline NUAK1/2 protein expression in these parenchymal cells (Figure S3I), WZ4003-mediated NUAK1/2 inhibition effectively suppressed the phagocytic capacity of both polarized and repolarized iPSC-derived microglia, mirroring our MΦ data (Figure S3J).

In conclusion, our data indicate that NUAK1 and NUAK2 inhibition modulates the phagocytic capacity of iPSC-derived MΦs and microglia, highlighting their potential relevance for reprogramming engulfment functions. This functional reprogramming appears to be mediated by NUAK1/2 inhibition, is fully reversible upon cpd removal, and was stringently validated using a multi-inhibitor panel (WZ4003, HTH-01-015, HTH-02-006) to exclude confounding off-target effects.

### 2.4. Mitochondrial Cell Painting Profiling Reveals NUAK1/2 Inhibition-Induced Alterations of MΦs Mitochondrial Bioenergetics

Recent studies in metabolic, oncologic, and fibrotic diseases have implicated NUAK1 in the regulation of cellular energy homeostasis and mitochondrial function through effects on mitochondrial dynamics, bioenergetics, TGF-β-signaling and oxidative stress responses [47,48,49,53,55,56,57]. While the role of NUAK1 in mitochondrial biology has been studied more extensively, the contribution of NUAK2 remains less clear [38]. Moreover, the impact of both kinases on mitochondrial function of MΦs is still under investigation.

Therefore, we assessed the mitochondrial function of NUAK1/2i-(re-)polarized IDMs by measuring the oxygen consumption rate (OCR) by real-time extracellular flux analysis. As an initial, we characterized baseline responses to established biological stimuli (Figure S4A). In contrast to LPS stimulation, IL-4 + IL-13 treatment increased both basal and maximal respiratory capacity (Figure S4A), consistent with reports that anti-inflammatory IDMs maintain a higher spare respiratory capacity. In agreement with the literature [58,59,60], human LPS-stimulated IDMs retained mitochondrial activity comparable to unstimulated controls (Figure S4A), differing from the metabolic switch to aerobic glycolysis commonly described in murine MΦs [58,59,60]. Assessing WZ4003-polarized IDMs, we found that NUAK1/2 inhibition reduced basal respiratory capacity, though function was maintained at baseline levels (Figure S4B). During repolarization experiments, all three NUAK1/2 inhibitors (WZ4003, HTH-01-015, and HTH-02-006) reduced both basal and maximal respiration compared to IL-4 + IL-13 controls (Figure S4B). Moreover, withdrawal of IL-4 + IL-13 before to NUAK1/2 inhibition (IL-4 + IL-13 + wash + NUAK1/2i) led to a more pronounced reduction in OCR values than continuous cytokine exposure (Figure S4B). These findings suggest that sustained IL-4 + IL-13 signaling may partially preserve mitochondrial respiratory capacity during reprogramming.

To further characterize cellular energy metabolism, we performed real-time ATP production rate assays. Untreated IDMs exhibited a balanced energy phenotype favoring glycolysis (a 1.36:1 ratio; Figure S4C). The initial enhancement of mitoATP (associated with OXPHOS) production by IL-4 + IL-13 was reduced upon repolarization with NUAK1/2 inhibitors, particularly when the cytokines were removed prior to repolarization, whereas glycoATP (associated with conversion of glucose to lactate) was largely unaffected (Figure S4C). Notably, mitochondrial mass did not differ substantially between conditions (Figure S4D), indicating that the observed bioenergetic changes were not accompanied by major alterations in total mitochondrial content.

**Figure 4:**
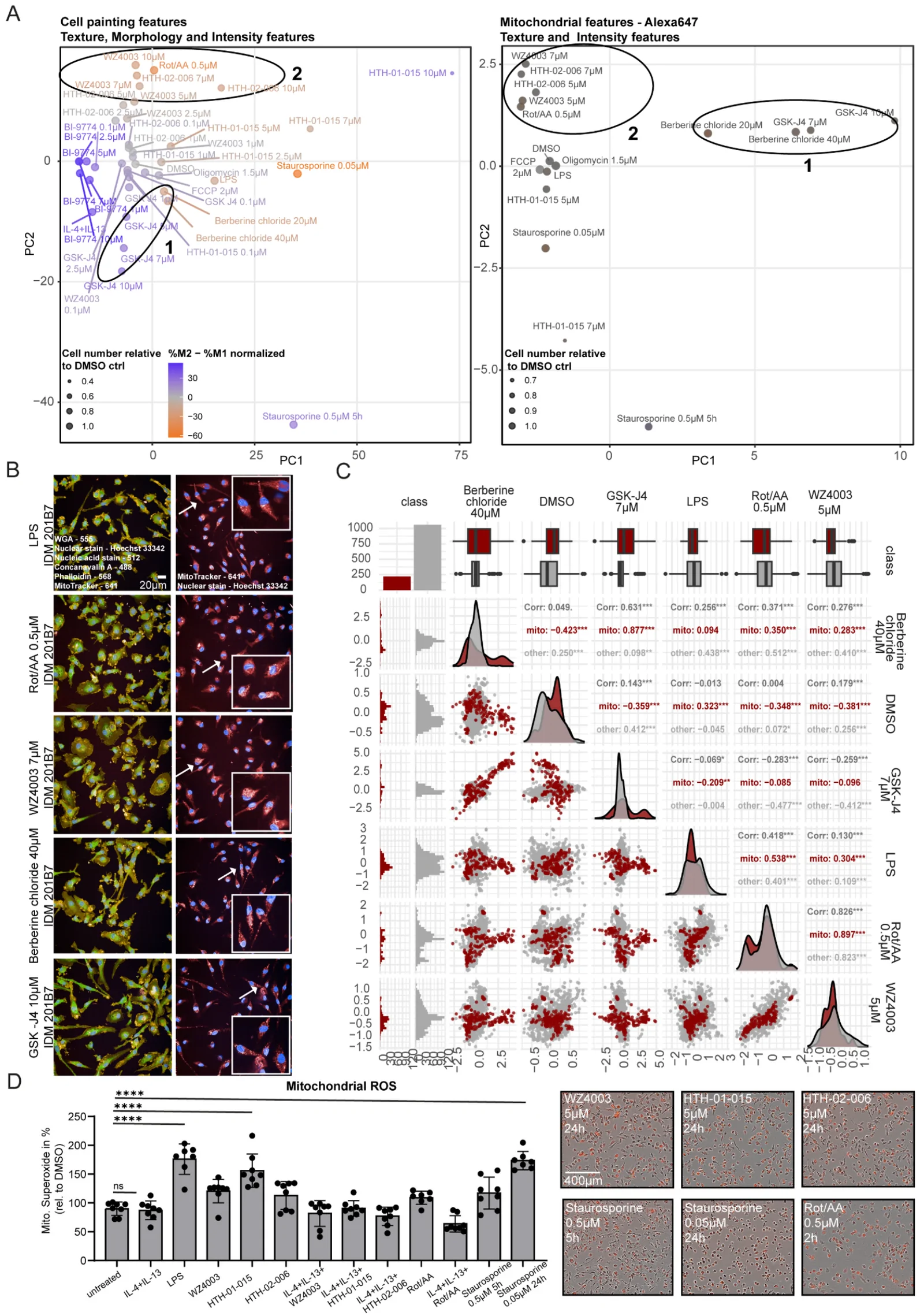
NUAK1/2 inhibition modulates mitochondrial morphological signatures and alters mitochondrial ROS levels. (**A**) Principal component analysis (PCA) of total cell painting feature profiling (1279 features) and targeted cell painting mitochondrial profiling (222 features) of NUAK1/2-inhibited IDMs. IDMs were treated for 24 h or 5 h with the indicated conditions (DMSO 0.1%, untreated, LPS 5 ng/mL, Rot/AA 0.5 µM, staurosporine 0.05 µM, WZ4003 5 and 7 µM, HTH-01-015 5 and 7 µM, HTH-02-006 5 and 7 µM, GSK-J4 7 and 10 µM, berberine chloride 20 and 40 µM, FCCP 2 µM, and oligomycin 1.5 µM). Data points represent the mean calculated across replicates (totaling *n* = 4 wells per condition, with 5 fields imaged per well at 20× magnification). The absolute cell count relative to the vehicle (DMSO) control is represented proportionally by the diameter of the data points. Annotations for Cluster 1 and Cluster 2 highlight distinct morphological and textural alterations induced in the mitochondrial channel by the respective cpds. (**B**) High-content imaging of MitoTracker-stained mitochondria in modulated IDMs. Representative high-content Opera Phenix™ confocal images (40× magnification; one representative field shown) illustrate cell-painted and polarized or repolarized IDMs. Cell painting was performed using PhenoVue™ Kit components (specifically: PhenoVue™ 641 Mitochondrial Stain, PhenoVue™ Hoechst 33342 Nuclear Stain, PhenoVue™ Fluor 488-Concanavalin A, PhenoVue™ 512 Nucleic Acid Stain, PhenoVue™ Fluor 555-WGA, and PhenoVue™ Fluor 568-Phalloidin). Images correspond to the treatment conditions analyzed in A; arrows indicate representative intracellular regions displaying structural mitochondrial alterations upon treatment. (**C**) Pearson correlation matrix comparing phenotypic profiles across selected treatment conditions. Pairwise correlation coefficients (r) are plotted between normalized feature profiles and do so for mitochondrial features (mito), other features (other), as well as all features (corr) combined across WZ4003, Rot/AA, LPS, and berberine chloride treatments, alongside the respective feature probability distributions. Asterisks denote statistically significant correlation coefficients (*** *p* < 0.001, ** *p* < 0.01, * *p* < 0.05). (**D**) Mitochondrial ROS quantification in NUAK1/2 inhibitor- and Rot/AA-treated IDMs. IDMs were treated for 24 h with the indicated conditions. For MΦ repolarization, cells were pre-treated for 24 h with IL-4 + IL-13 (20 ng/mL) prior to a subsequent 24 h co-treatment with the respective cpds. Mitochondrial superoxide levels are expressed as a percentage relative to the vehicle control (data depicted as mean ± SD; *n* = 4 replicates per condition), monitored using an IncuCyte^®^ SX3 live-cell imaging system (quantification at 30 min post-probe addition; 10× magnification). Staurosporine (0.05 µM) and Rot/AA (0.5 µM) served as internal controls for mitochondrial stress and superoxide induction at the indicated time points. Representative live-cell phase-contrast images are overlaid with the fluorescent mitochondrial superoxide probe (red). Statistical analysis was performed using a one-way ANOVA followed by Dunnett’s post hoc test for comparisons against the vehicle control (DMSO 0.1%), and an unpaired *t*-test for the specific comparison between untreated and IL-4 + IL-13 groups; asterisks denote statistical significance (**** *p* < 0.0001, ns = non-significant).

Due to these bioenergetic shifts, we re-analyzed our primary screen images by performing targeted mitochondrial profiling (222 features in the 647 channel). In the primary screen, the staining control berberine chloride [61,62] and the JUMP-CP cpd GSK-J4 shared similar profiles, showing pronounced alterations in the mitochondrial channel (cluster 1; Figure S4E,F). To contextualize these findings, we expanded our panel with diverse mitochondrial modulators (FCCP, oligomycin, Rot/AA, and staurosporine). Interestingly, dose-dependent NUAK1/2 inhibition using HTH-01-015, WZ4003, and HTH-02-006 yielded global cell painting and mitochondria-specific profiles that clustered with the Rot/AA treatment (cluster 2; Figure 4A). Consistent with this observation, we found a strong Pearson correlation between Rot/AA and WZ4003 for both total cell painting features (r = 0.826) and mitochondrial features (r = 0.897; Figure 4C). In contrast, weaker correlations were found between Rot/AA and LPS or berberine chloride treatment. Interestingly, although LPS, Rot/AA, and NUAK1/2i all induce a rounded M1-like phenotype, Rot/AA-treated IDMs did not show reduced phagocytic activity (Figure S4G), indicating the functional plasticity of MΦ states.

To further investigate mitochondrial responses, we quantified ROS in the mitochondria by a MitoSOX assay. As expected, Rot/AA treatment led to mitochondrial ROS accumulation (mitoROS) in IDMs due to electron leakage induced by complex I/III blockage, forming superoxides with oxygen. Interestingly, NUAK1/2 inhibition induced a comparable, albeit modest, increase in mitochondrial ROS accumulation (Figure 4D). In contrast, LPS exposure produced a more pronounced increase in mitochondrial ROS levels. During repolarization experiments, we detected no mitoROS accumulation in reprogrammed MΦs by NUAK1/2i or Rot/AA (Figure 4D). Furthermore, total intracellular ROS levels remained largely unchanged across polarized and repolarized conditions (Figure S4H). These findings may indicate that MΦs exposed to IL-4 + IL-13 are better able to buffer mitoROS induction by NUAK1/2 inhibition, potentially due to enhanced respiratory capacity and antioxidant defense mechanisms. Next, we measured the ratio of GSH/GSSG under unstimulated, polarized and repolarized conditions. As shown in Figure S4I, IL-4 + IL-13 stimulated IDMs show elevated GSH/GSSG ratios that are also present upon repolarized conditions with NUAK1/2 inhibition.

In summary, our data indicate that NUAK1/2 inhibition modulates mitochondrial function and mitoATP production in polarized and repolarized MΦs. Mitochondrial cell painting profiling revealed a morphological signature that resembled the phenotype induced by Rot/AA treatment, consistent with the observed accumulation of mitochondrial ROS under both conditions. Combining our mitochondrial cell painting analysis pipeline with functional in vitro assays, such as ROS measurements or phagocytosis read-outs, showed a spectrum of rounded M1-like phenotypes across LPS, Rot/AA, and NUAK1/2 inhibitor treatments, each characterized by distinct functional profiles.

### 2.5. NUAK1/2 Inhibition Leads to an Antioxidative Stress Response Adaptation and Protection from RSL3-Induced Ferroptosis in MΦs

Mitochondrial ROS regulation links mitochondrial function to cellular redox dynamics [63,64], governing MΦ survival or death [65,66,67,68,69]. Inflammatory stimuli disrupt redox balance and promote oxidative stress, triggering ferroptosis, a regulated iron-dependent form of cell death [70,71,72,73]. Because NUAK1/2 inhibition modulates mitochondrial ROS levels in IDMs, we investigated its impact on ferroptotic cell fate decisions.

**Figure 5:**
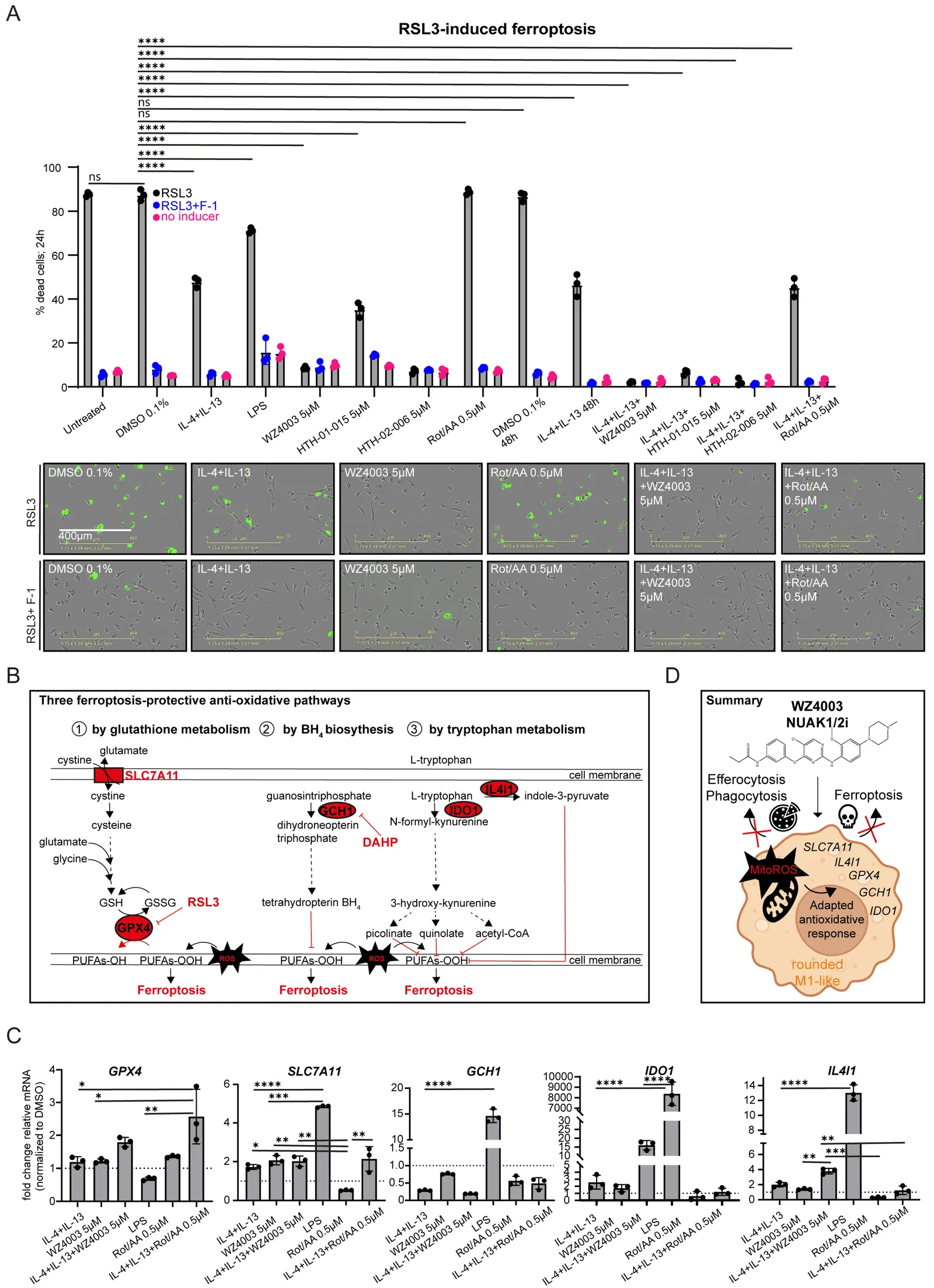
Quantification of RSL3-induced ferroptosis in phenotypically modulated IDMs. (**A**) IDMs (201B7 line) were treated for 24 h or 48 h with the indicated conditions (DMSO 0.1%, untreated, LPS 5 ng/mL, IL-4 + IL-13 20 ng/mL, WZ4003 5 µM, HTH-01-015 5 µM, HTH-02-006 5 µM, and Rot/AA 0.5 µM). For MΦ repolarization, cells were pre-treated for 24 h with IL-4 + IL-13 (20 ng/mL) prior to a subsequent 24 h co-treatment with the respective small molecules. To induce and modulate ferroptosis, cells were challenged with RSL3 (0.1 µM) in the presence (blue data points/bars) or absence (black data points/bars) of the ferroptosis inhibitor ferrostatin-1 (F-1, 1 µM); a control group without any cell death inducer (magenta data points/bars) was evaluated in parallel. Quantitative data represent the percentage of dead cells determined by cell-impermeant CellTox™ Green dye fluorescence, monitored using an IncuCyte^®^ SX3 live-cell imaging system (data depicted as mean ± SD; *n* = 4 replicates per condition; quantified at the 24 h post-challenge time point; 10× magnification). Representative live-cell phase-contrast images are split into RSL3 challenge alone (top row) and RSL3 + F-1 co-treatment (bottom row), overlaid with the green fluorescent cell death probe. Statistical analysis was performed using a two-way ANOVA followed by Tukey’s multiple comparisons test to evaluate differences between the respective treatment conditions and control groups; asterisks denote statistical significance (**** *p* < 0.0001, ns = non-significant). (**B**) Schematic illustration of three major antioxidant metabolic pathways implicated in ferroptosis defense. The illustrated pathways comprise: (1) glutathione metabolism mediated by SLC7A11 and GPX4, (2) BH_4_ biosynthesis regulated by GCH1, and (3) tryptophan metabolism mediated by IDO1 and IL4I1 (created with BioRender.com, Adobe Illustrator, and ChemDraw). (**C**) Relative transcript abundance of key genes involved in antioxidant ferroptosis defense pathways. IDMs were treated for 24 h as described in A, in the absence of RSL3 or F-1. Quantitative data represent vehicle (DMSO)-normalized fold-changes in mRNA expression levels for GPX4, SLC7A11, GCH1, IDO1, and IL4I1 (data depicted as mean ± SEM; *n* = 3 replicates per condition). Statistical analysis was performed using a one-way ANOVA followed by Dunnett’s multiple comparisons test against the vehicle control (DMSO 0.1%) or between the specifically indicated pairing groups; asterisks denote statistical significance (**** *p* < 0.0001, *** *p* < 0.001, ** *p* < 0.01, * *p* < 0.05). (**D**) Summary diagram of the proposed mechanism. The schematic illustrates how WZ4003-mediated NUAK1/2 inhibition shifts MΦs toward a distinct, rounded M1-like state characterized by elevated mitochondrial ROS production, an adapted antioxidant gene program, protection against ferroptosis, and attenuated phagocytic and efferocytic capacities (created with BioRender.com, Adobe Illustrator, and ChemDraw).

Accumulation of lipid hydroperoxides, particularly oxidized polyunsaturated phospholipids (PUFAs) in cellular membranes, drives ferroptosis. RSL3 is a well-studied ferroptosis inducer that targets the selenoprotein GPX4 (Glutathione Peroxidase 4), which directly detoxifies lipid hydroperoxides using GSH as a cofactor [74], as well as TXNRD1 (Thioredoxin Reductase 1). Consistent with previous studies [60,75,76], we found polarization-dependent differences in ferroptosis sensitivity: IL-4 + IL-13-polarized MΦs were more protected from RSL3-induced ferroptosis than LPS-polarized cells (Figure 5A), potentially reflecting their higher mitochondrial respiratory capacity (Figure S4A). Of note, LPS-polarized MΦs showed enhanced basal ferroptosis levels, even in the absence of RSL3. Treatment with the ferroptosis inhibitor ferrostatin-1 (F-1) reversed RSL3-induced cytotoxicity, confirming ferroptosis as the predominant mode of cell death (Figure 5A) [77]. Interestingly, NUAK1/2-inhibited IDMs were more protected from RSL3 than LPS-polarized or Rot/AA-treated cells (Figure 5A and Figure S5A). Repolarization provided protection, an effect further supported by time-course analyses demonstrating reduced cell death kinetics compared with polarized cells (Figure S5A). Consistent with a role for IL-4 + IL-13 in mitochondrial fitness, co-treatment with IL-4 + IL-13 and Rot/AA reduced RSL3-induced ferroptosis by 43.5% after 24 h (Figure 5A and Figure S5A).

A link between NUAK2 and ferroptosis has previously been identified in breast cancer cells, where NUAK2 suppresses the expression of *GPX4* at the mRNA level and enhances ferroptosis triggered by GPX4 inhibitors [78]. Beyond GSH/GPX4 metabolism, MΦs can engage alternative ferroptosis defense mechanisms: tetrahydrobiopterin (BH_4_) biosynthesis and tryptophan catabolism (Figure 5B) [79,80]. GTP Cyclohydrolase 1 (GCH1)-mediated BH_4_ production provides a GPX4-independent antioxidant pathway by suppressing lipid peroxidation [81,82]. In this regard, a recent study [60] highlighted a GCH1–BH_4_ axis that governs MΦ ferroptosis susceptibility. We phenocopied their experimental setup and found that NUAK1/2-inhibited MΦs remained ferroptosis protected upon pharmacological GCH1 inhibition with DAHP (2,4-Diamino-6-hydroxypyrimidine) (Figure S5B). However, as neither GCH1 activity nor intracellular BH_4_ levels were directly measured, a contribution of this pathway cannot be excluded.

Transcriptional analysis indicated that NUAK1/2 inhibition moderately upregulates *GPX4* and *SLC7A11* (cystine/glutamate antiporter system Xc^−^) whereas *GCH1* expression remained unchanged (Figure 5C). In contrast, the tryptophan-catabolizing enzymes *IDO1* and *IL4I1* were strongly induced (Indoleamine 2,3-Dioxygenase 1 and Interleukin-4-Induced-1; [83]) (Figure 5C). Both enzymes generate metabolites with reported antioxidant and redox-regulatory properties, suggesting an alternative mechanism supporting ferroptosis protection [80,84,85,86]. Nevertheless, these associations remain correlative rather than causative, as transcript abundance did not consistently mirror responses across Rot/AA, LPS, and WZ4003 treatments (Figure 5C).

Together, these findings suggest that all three M1-like agents (LPS, Rot/AA and NUAK1/2i) employ different adaptive antioxidant programs in response to mitochondrial ROS stress, thereby shaping their sensitivity to ferroptosis.

In summary, NUAK1/2 inhibition protected MΦs against RSL3-induced ferroptosis that was further enhanced by polarization with IL-4 + IL-13, whereas Rot/AA-treated only cells remained sensitive. Collectively, our data indicate that LPS-, Rot/AA-, and NUAK1/2i-induced MΦ states, despite sharing M1-like characteristics, result in distinctive functional outcomes across ferroptosis, mitochondrial function, and phagocytic capacity. 

## 3. Discussion

Pharmaceutical companies and academic institutions increasingly employ phenomics-based approaches, including cell painting and image-based profiling, to characterize cpd-induced cellular states and support drug discovery efforts in areas like oncology and neuroscience. By combining high-content imaging with ML, these approaches facilitate the systematic comparison of perturbed cell states and may assist in the identification and prioritization of cpds with shared or distinct biological activities [22]. The growing interest in this field is reflected by several phenomics-derived drug candidates currently undergoing clinical evaluation (Recursion.com).

In the present study, we integrated our previously published MΦ cell painting assay and ML-based analysis pipeline [11] into a high-content screening platform for early drug discovery (Figure 1). We aimed to identify previously unrecognized MΦ-polarizing cpds from annotated libraries and to explore MΦ phenotypic diversity beyond conventional marker-based approaches. The resulting dataset expands the available maps of phenotypic MΦ responses and may provide a useful resource for future studies investigating MΦ polarization and plasticity.

To facilitate target interpretation, we screened annotated cpds from two publicly available small molecule libraries with well-characterized MoA and documented bioactivity in human cells (Table S1). Furthermore, we employed two MΦ models: human IDMs and blood-derived MDMs from several donors. This experimental design enabled the prioritization of cpds that generated consistent phenotypic effects across different donors and cellular backgrounds. The use of both systems may help mitigate limitations associated with donor-to-donor variability, limited availability of blood donor-derived cells, and the altered biology often reported for MΦ-like tumor cell lines such as THP-1 or U937 [33,87].

Across our hit validation workflow, approximately 5% of the evaluated cpds were confirmed as reproducible modulators of MΦ polarization in both IDMs and MDMs. Notably, the validated hit list included several cpds with shared molecular targets, related chemical scaffolds, or overlapping MoA, in addition to previously described myeloid-polarizing cpds such as arcyriaflavin A and SU11274 [8]. Nevertheless, potential off-target activities cannot be excluded, particularly given that many kinase inhibitors display complex target engagement profiles. Therefore, the specific molecular mechanisms underlying the observed phenotypes will require further investigation using complementary approaches, including chemical proteomics, transcriptomics and genetic perturbation studies.

Consistent with previous reports highlighting MΦ heterogeneity, donor-dependent variability was also observed among MDM populations in the present study (Figure 1 and Figure 2). While our analysis was based on median cell painting profiles, future studies employing single-cell morphological profiling may provide additional insights into the diversity and distribution of MΦ polarization states at the individual cell level.

Interestingly, our screening approach identified cpds targeting members of the ARK family that induced opposing MΦ phenotypes. Specifically, WZ4003, a dual NUAK1/2 inhibitor identified from the JUMP-CP library, induced a reproducible and dose-dependent shift toward round M1-like cell shapes in IDMs and MDMs. In contrast, pharmacological activation of AMPK by BI-9774 promoted an elongated, M2-like morphology (Figure 1 and Figure 2). These morphological changes were accompanied by distinct functional responses. WZ4003-treated cells exhibited an approximately 2-fold reduced efferocytosis of apoptotic Raji cells, whereas BI-9774 treatment slightly enhanced clearance capacity in IDMs (Figure 2). Similarly, live cell phagocytosis assays using *E. coli* bioparticles confirmed opposing effects of pharmacological NUAK1/2 inhibition and AMPK activation on particle uptake (Figure 3). Furthermore, inhibition of NUAK1/2 using three publicly available cpds (WZ4003, HTH-01-15, HTH-02-006) consistently reduced phagocytic activity in IDMs and iPSC-derived microglia (Figure 3), supporting a potential role of NUAK1/2 signaling in regulating cellular clearance functions.

In addition, mitochondrial cell painting analyses and extracellular flux measurements indicated that pharmacological NUAK1/2 inhibition induces a profile of mitochondrial electron transport chain restriction via Rot/AA exposure (Figure 4). Together with the observed increase in mitochondrial superoxide levels, these findings suggest that pharmacological NUAK1/2 inhibition affects mitochondrial homeostasis. We further observed activation of antioxidant response pathways and reduced sensitivity to RSL3-induced ferroptosis (Figure 4 and Figure 5). Although these observations are consistent with an adaptive response to increased mitochondrial ROS, the mechanistic relationship between NUAK1/2 signaling, mitochondrial dysfunction, ROS generation, antioxidant adaptation, and ferroptosis protection remains to be established experimentally.

Taken together, our data indicate that pharmacological inhibition of NUAK1/2 is associated with a distinct MΦ phenotype characterized by specific morphological, metabolic, and functional features. Further studies will be required to determine the molecular basis and physiological relevance of this MΦ state in more complex disease settings.

Previous studies have reported divergent effects of NUAK inhibition on oxidative stress pathways [88,89]. For example, shRNA-mediated depletion of NUAK2 in A549 cells was associated with increased lipid ROS accumulation and enhanced ferroptosis sensitivity [90]. In contrast, our observation of elevated mitochondrial ROS levels following WZ4003 treatment is consistent with findings reported in HCT116 tumor cells. These observations suggest that the impact of NUAK1/2 signaling on cellular redox homeostasis may be highly cell type- and context-dependent. Importantly, despite the induction of mitochondrial ROS, NUAK1/2 inhibition in our system was associated with activation of antioxidant response pathways and reduced susceptibility to ferroptosis, indicating a more complex relationship between ROS generation and ferroptotic cell death than would be predicted from ROS levels alone.

Moreover, whether the MΦ phenotype associated with pharmacological NUAK1/2 inhibition could prove beneficial in a therapeutic context will require further investigation in more complex in vitro systems and appropriate preclinical disease models. Existing in vivo data do not currently indicate overt toxicity associated with systemic NUAK1/2 inhibition. For example, treatment with 9q, a WZ4003-derived NUAK1/2 inhibitor lacking a methoxy group, did not result in obvious adverse effects in a SW480 colorectal cancer xenograft model [51]. However, such observations should be interpreted cautiously, as the safety, efficacy, and immunological consequences of sustained NUAK1/2 inhibition remain insufficiently characterized across different disease settings.

Nevertheless, several aspects of the observed phenotype warrant discussion in the context of disease-relevant MΦ functions.

One aspect of the reduced phagocytic activity induced by pharmacological NUAK1/2 inhibition is the increasingly recognized role of MΦ as regulators of tumor immunity, leading to growing interest in MΦ reprogramming as a potential therapeutic strategy in cancer [6,91,92]. One prominent example is the CD47–SIRPα axis. CD47, frequently overexpressed by tumor cells, acts as a “don’t eat me” signal that suppresses MΦ-mediated phagocytosis through engagement of SIRPα on tumor-associated MΦs (TAMs). Therapeutic blockade of this pathway has been shown to enhance the phagocytic clearance of viable tumor cells and is currently being evaluated in clinical studies [93,94]. In this context, the reduced phagocytic activity observed following NUAK1/2 inhibition could potentially limit MΦ-mediated tumor cell clearance. Conversely, accumulating evidence suggests that efferocytosis of apoptotic tumor cells can promote immunoregulatory and M2-like MΦ programs that may support tumor progression [95]. Consequently, signaling pathways linked to efferocytosis are increasingly explored as potential targets for cancer immunotherapy. Within this context, the reduced efferocytic activity observed following pharmacological NUAK1/2 inhibition may be of interest. However, whether such effects would ultimately enhance or impair anti-tumor immunity is difficult to predict due to the context-dependent and often opposing functions of MΦ -mediated clearance pathways within the tumor microenvironment.

Several limitations should be acknowledged: First, we focused exclusively on in vitro systems and therefore did not address the role of NUAK1/2 in disease-relevant tissue environments or in vivo models. Future studies employing disease-relevant in vitro systems and in vivo models will be required to determine whether NUAK1/2 inhibition can modulate MΦ functions in a therapeutically meaningful manner [95,96,97]. Second, the molecular mechanisms linking NUAK1/2 inhibition to MΦ polarization remain incompletely understood. Given the role of NUAK1/2 substrates such as MYPT1 (myosin phosphatase target subunit 1) [98,99,100] in regulating actin cytoskeleton dynamics, it will be important to determine how alterations in cytoskeletal dynamics contribute to the observed MΦ phenotypes. Third, CRISPR-Cas9-mediated knockout or siRNA-based approaches, potentially combined with multi-omics, will be required to determine whether these functional outcomes are driven by NUAK1, NUAK2, or both. In addition, this approach would expand more broadly on the functional and biochemical consequences of NUAK1/2i-induced MΦ polarization beyond the ones indicated in this study. Finally, although our data implicate mitochondrial ROS accumulation, antioxidant response adaptation, phagocytic/efferocytic impairment and ferroptosis protection as key downstream processes upon NUAK1/2 inhibition, a causal relationship among these functions remains to be established using tools like iron chelators, lipid peroxides, cell stress inducers or ROS scavengers. Combining multi-omics with cell painting will also provide target and pathway engagement and link cell painting classification with MΦ polarization.

Collectively, our study illustrates the utility of ML-assisted phenotypic screening of cell-painted MΦs for the identification of MΦ-polarizing cpds. Using this approach, we identified NUAK1/2 inhibitors as modulators of MΦ polarization, mitochondrial homeostasis, phagocytic and efferocytic activity, and ferroptosis sensitivity. Further studies will be required to define the underlying molecular mechanisms and to determine the relevance of these findings in disease-relevant settings.

## 4. Materials and Methods

### 4.1. Isolation of Human Monocytes from Leukapheresis Products and Differentiation Toward MΦs

In accordance with ethical standards and local regulations, anonymized leukapheresis products from healthy human volunteers (donors) were obtained from Deutsches Rotes Kreuz (Ulm, Germany). The donors (CL090, CL091, CL541, CL415, and CL414) that were used in this study are the same as those mentioned in [11]. For all donors, human peripheral blood mononuclear cells were isolated and differentiated toward MDMs, and quality checked as previously published in [11]. MDMs were cultivated in complete DMEM GlutaMAX (31966-021, Gibco Thermo Fisher Scientific, Karlsruhe, Germany) supplemented with 10% FCS (10500064, Gibco Thermo Fisher Scientific, Karlsruhe, Germany, LOT 10500-064) and 1% NEAA (11140050, Gibco Thermo Fisher Scientific, Karlsruhe, Germany) in the presence of 100 ng/mL recombinant human M-CSF (216-MC-500, R&D Systems, Wiesbaden, Germany).

### 4.2. Differentiation of Human iPSC Line CD34^+^-Derived Progenitors Toward MΦs

201B7 [30] and Cellartis human iPSC line 12 (ChiPSC12; cat. no. Y00285, Takara Bio Europe AB; Saint Germain-en-Laye; France) were cultured according to the manufacturer’s instructions in a feeder-free environment as a monolayer. The quality control (pluripotency and differentiation stage), the maintenance, and the differentiation of both lines toward CD34^+^-derived MΦs were performed as mentioned in [11]. IDMs were cultivated in complete DMEM GlutaMAX (31966-021, Gibco Thermo Fisher Scientific, Karlsruhe, Germany) supplemented with 10% FCS (10500064, Gibco Thermo Fisher Scientific, Karlsruhe, Germany, LOT 10500-064) and 1% NEAA (11140050, Gibco Thermo Fisher Scientific, Karlsruhe, Germany) in the presence of 100 ng/mL recombinant human M-CSF (216-MC-500, R&D Systems, Wiesbaden, Germany).

### 4.3. Cultivation of Human iPSC-Derived Microglia

Human iPSC-derived microglia (iPSC-derived iCell^®^ Microglia, donor 01279; cat. no. R1131; TREM2^+^, CX3CR1^+^, TMEM119^+^, P2RY12^+^, and IBA1^+^) were purchased from FUJIFILM Cellular Dynamics (Madison, WI, USA). The cells were derived at FUJIFILM Cellular Dynamics (Madison, WI, USA) following the method described by [101]. According to the manufacturer’s instructions, the cells were kept for three days in iCell Glial Base Medium (with supplements A, B, C) in the respective poly-D-lysine-coated plates (655946 for a 96-well plate or 657940 for a 6-well plate; Greiner, Frickenhausen, Germany) until in vitro assays were performed.

### 4.4. Cell Painting High-Content Screening

The cell painting high-content screen was performed automatically in the in-house automated screening facility with robotic handlers (TX90XL, Stäubli, Bayreuth, Germany) regulated by the Momentum™ software (version 7.6, Thermo Fisher Scientific, Karlsruhe, Germany) using a CyBio QuadStack (Analytik Jena, Jena, Germany) for plate storage, a CERTUS Flex (Fritz Gyger AG, Thun, Switzerland) for staining and biological control stimulation, a CyBi-Well CyBio Automated Simultaneous Pipettor (ARTISAN. Lüchow, Germany) for cpd library transfer, a Multidrop^TM^ Combi Reagent Dispenser (Thermo Fisher Scientific, Karlsruhe, Germany) for PFA fixation, a Bio Tek ELX405 (BioTek, Waldbronn, Germany) for washing processes, a Cytomat™ 10 (Thermo Fisher Scientific, Karlsruhe, Germany) and Liconic STX220 incubator (Liconic Instruments, Montabaur, Germany) for cell plate-incubation, automated Roll Heat Sealer (formerly a4S, AZENTA Life Sciences, Griesheim, Germany) for plate sealing, a barcode reader SR-1000 (Keyence, Frankfurt am Main, Germany) for plate barcoding, a LidPark (F01318, Thermo Fisher Scientific, Karlsruhe, Germany) for lid removal and an Opera Phenix^TM^ confocal imaging system for imaging. The screen was performed per cell model over one week and split into five process parts: IDM and MDM generation, library generation, cpd library treatment, cell painting and confocal imaging. In this context, MΦs (IDMs and MDM donors CL090, CL091, CL415 and CL541) were quality checked by FACS analysis for CD14, CD45, CD34, CD206, CD163, CD80 and CD86 expression (mentioned in [11]) and seeded at a specific density (IDMs: 9000 cells/well; MDMs: 5000 cells/well; 45 µL MΦ cultivation medium/well) in barcoded black 384-well Phenoplates (6057300302, Revvity, Hamburg, Germany) using a Multidrop^TM^ Combi Reagent Dispenser (5840300, Thermo Fisher Scientific, Karlsruhe, Germany). To minimize edge effects, cell seeding was restricted to the inner wells of the plate, while the peripheral wells were filled with 1× PBS (10010023, Gibco Thermo Fisher Scientific, Karlsruhe, Germany). After overnight incubation of the black 384-well Phenoplates (6057300302, Revvity, Hamburg, Germany) at 37 °C and 5% CO_2_, the cells were treated and stimulated with the cpd libraries (2 plates of JUMP-CP with 396 cpds and one plate of opnMe with 144 cpds per cell model; Table S1) and the internal control conditions (cpd and biological controls) following storage for 24 h at 37 °C and 5% CO_2_. In detail, 5 µL per condition in a pre-prepared 384-well deep-well storage plate (784201, Greiner, Frickenhausen, Germany) was transferred into the black 384-well Phenoplate (6057300302, Revvity, Hamburg, Germany) automatically with the CyBi-Well CyBio Automated Simultaneous Pipettor (69917-2, ARTISAN, Lüchow, Germany). For both libraries, each cpd was transferred in duplicate per tested concentration (1 µM and 10 µM) to enable each replicate at the same well position on both barcoded black 384-well Phenoplates (6057300302, Revvity, Hamburg, Germany). The following internal biological controls were used in their indicated final concentrations: 20 ng/mL rh IL-4 (201-IL/CF 10 µg, R&D Systems, Wiesbaden, Germany), 20 ng/mL rh IL-13 (213-IL/CF 25 µg, R&D Systems, Wiesbaden, Germany), 5 ng/mL LPS (L6143 from Salmonella, Sigma Aldrich, Schnelldorf, Germany) and untreated (only MΦ cultivation medium). Each condition was diluted with DMSO (12611S, CST, Frankfurt am Main, Germany) to reach a final concentration of 0.1%. The following internal control cpds were used at different concentrations mentioned in the respective figure legend: thiostrepton (598226, Sigma Aldrich, Schnelldorf, Germany), bosutinib (PZ0192, Sigma Aldrich, Schnelldorf, Germany), fenbendazole (1269403, USP, Basel, Switzerland), alsterpaullone (A4847, Sigma), etoposide (D1225, Selleckchem, Cologne, Germany), CA-074Me (205531, Millipore, Darmstadt, Germany), cytochalasin D (C8273, Sigma Aldrich, Schnelldorf, Germany), berberine chloride (PHR1502, Sigma Aldrich, Schnelldorf, Germany) and tetrandrine (S2403, Selleckchem, Cologne, Germany). For the internal control conditions (cpds and biological stimuli), extra stimulated plates without library cpds were used. Next, the MΦs were cell-painted and images were acquired using the Harmony software (version 5.2) of the Opera Phenix^TM^ and processed for data analysis in the SImA1.3 software from Revvity, Hamburg, Germany, as previously described by [11]. The opnMe library (active and inactive probes; Table S1) was purchased from the opnMe^®^ platform from Boehringer Ingelheim GmbH & Co. KG and the JUMP-CP library (partial; Table S1) was purchased from Specs (eMolecules—Zoetermeer, The Netherlands).

### 4.5. Cell Roundness Analysis

The cell roundness analysis was conducted as mentioned in [11]. The cell roundness values are 0.1% DMSO-normalized and based on 20× magnified images. The threshold for M1-like and M2-like hits was set according to the internal cpd controls bosutinib (1 µM), alsterpaullone (1 and10 µM), thiostrepton (2.5 µM) and fenbendazole (1 µM). Data were visualized using GraphPad Prism v10.1.2 (GraphPad Software, San Diego, CA, USA) and Adobe Illustrator 10 (2022, version 26.4).

### 4.6. Feature-Based Cell Painting Analysis

The feature-based cell painting analysis was setup and performed referring to [11]. The cell painting features from texture, intensity and morphology properties were determined in all four channels (Alexa488, Alexa568, Alexa647 and Hoechst33342) over three regions (Cytoplasm, Nucleus and Full Cell) using the customized cell painting image analysis pipeline (1279 features in total).

Established in [11], M2, M1 and M0 classification was performed by a linear classifier trained with the PhenoLOGIC™ classification algorithm in the SImA1.3 software from Revvity, Hamburg, Germany. Each class was trained on over 100 manually annotated IDMs using internal M1- and M2-like cpd controls (bosutinib 1 μM, alsterpaullone 1 μM, thiostrepton 2.5 μM and fenbendazole 1 μM) and M0 controls (0.1% DMSO, untreated) as training conditions. A linear combination of a subset of features for optimal class separation was automatically selected by the PhenoLOGIC™ classification tool (Supplementary File S2). The classifier was used to assign cells to one of the three predefined classes, class percentages were calculated on a per well level and normalized by subtraction of the respective class percentages in DMSO treated wells (%M0 − %M0_DMSO_, %M1 − %M1_DMSO_ and %M2 − %M2_DMSO_) to calculate the DMSO-normalized mean %M2 − %M1 values. In addition, the cell number was calculated to estimate cpd-induced cytotoxicity. For dimensionality reduction features were centered by subtraction of the mean and scaled by dividing by each features standard deviation. Uninformative features were removed by a “near-zero variance” filter and highly correlated features filtered using a pairwise correlation threshold of (0.99). The remaining features were then subjected to PCA calculating the first 2 principal components accounting for the highest amount of variance in the dataset (Supplementary File S1).

To evaluate treatment-induced changes across channels, we compared normalized feature values to DMSO 0.1% using a *t*-test and applied Bonferroni adjustment on the derived *p*-values (GraphPad Software, San Diego, CA, USA).

To evaluate morphological similarity between cpds, we computed the Pearson correlation between normalized feature profiles and do so for mitochondrial features, other features as well as all features combined.

### 4.7. LDH Assay

IDMs were seeded at a density of 0.3 × 10^5^ cells/well in triplicates in a white opaque 96-well plate (353296, Falcon Corning, Kaiserslautern, Germany) and treated for 24 h or 48 h with the respective conditions mentioned in the corresponding figure legends. The LDH assay was performed as described by [11]. 10% triton X-100 served as the internal positive control to determine the cytotoxicity of each tested condition indicated in the respective figure legend. Data were visualized using GraphPad Prism v10.1.2 (GraphPad Software, San Diego, CA, USA) and Adobe Illustrator 10 (2022, version 26.4).

### 4.8. Efferocytosis Assay

The efferocytosis assay was performed as described by [28]. In brief, MDMs or IDMs were resuspended in serum-free complete medium (DMEM GlutaMAX (31966-021, Gibco Thermo Fisher Scientific, Karlsruhe, Germany) supplemented with 1% NEAA (11140050, Gibco Thermo Fisher Scientific, Karlsruhe, Germany) in the presence of 100 ng/mL recombinant human M-CSF (216-MC-500, R&D Systems, Wiesbaden, Germany)) containing 0.5 µM CellTracker^TM^ Green CMFDA dye (C7025, Invitrogen, Darmstadt, Germany) and incubated for 45 min at 37 °C and 5% CO_2_. After washing in serum-containing complete medium ((DMEM GlutaMAX (31966-021, Gibco Thermo Fisher Scientific, Karlsruhe, Germany) supplemented with 10% heat-inactivated fetal calf serum (FCS; 10500064, Gibco Thermo Fisher Scientific, Karlsruhe, Germany; LOT 10500-064) and 1% NEAA (11140050, Gibco Thermo Fisher Scientific, Karlsruhe, Germany) in the presence of 100 ng/mL recombinant human M-CSF (216-MC-500, R&D Systems, Wiesbaden, Germany)), the MΦs were seeded at a specific density (IDMs: 9000 cells/well; MDMs: 5000 cells/well) into a black 384-well Phenoplate (6057300302, Revvity, Hamburg, Germany) in 20 µL serum-containing complete medium using a Multidrop^TM^ Combi Reagent Dispenser (5840300, Thermo Fisher Scientific, Karlsruhe, Germany). After overnight incubation at 37 °C and 5% CO_2_, the MΦs were treated for 24 h or 48 h with the respective conditions in quadruplicate, as mentioned in the figure legends before prey cell addition. Raji suspension cells (CCL-86; ATCC, Wesel, Germany) were used as prey cells, cultivated in RPMI1640 + GlutaMAX-I medium (61870–010, Gibco Thermo Fisher Scientific, Karlsruhe, Germany) with 10% fetal bovine serum (16000–044, Gibco Thermo Fisher Scientific, Karlsruhe, Germany) and 1% P/S at 37 °C and 5% CO_2_. Raji cells were UV-C-irradiated in a UV chamber for 7 min and pHrodo Red -labeled based on the manufacturer’s instructions of the IncuCyte^®^ pHrodo^®^ Red Cell Labeling Kit (4649, Sartorius, Göttingen, Germany). After one hour, the labeled and UV-C-irradiated Raji cells (0.3 × 10^5^ cells/well in 20 µL) were added to the previously seeded MΦs into a black 384-well Phenoplate (6057300302, Revvity, Hamburg, Germany) in 20 µL serum-containing complete medium using a Multidrop^TM^ Combi Reagent Dispenser (5840300, Thermo Fisher Scientific, Karlsruhe, Germany). Five hours after incubation at 37 °C and 5% CO_2_, the black 384-well Phenoplate (6057300302, Revvity, Hamburg, Germany) was measured using a confocal Opera Phenix^TM^ high-content screening device with the Harmony software (Revvity, Hamburg, Germany) measuring five fields per well at 20× magnification. Image processing and analysis were performed in the SImA1.3 software (Revvity, Hamburg, Germany) based on the algorithm established by [28]. The percentage of efferocytosing MΦs was calculated by the following equation: efferocytosing MΦ s [%] = (number of efferocytosis positive MΦs/total number of MΦs) ∗ 100. As internal negative and positive controls, cytochalasin D (1 µM for 1 h treatment; PHZ1063, Invitrogen, Darmstadt, Germany) and dexamethasone (3 µM for 24 h treatment; D1756, Sigma Aldrich, Schnelldorf, Germany) were used. Data were visualized using GraphPad Prism v10.1.2 (GraphPad Software, San Diego, CA, USA) and Adobe Illustrator 10 (2022, version 26.4).

### 4.9. Phagocytosis Assay

IDMs (201B7 or ChiPSC12 lines) or iPSC-derived microglia (iCell^®^ Microglia, FUJIFILM Cellular Dynamics, Madison, WI, USA) were seeded at a density of 0.3 × 10^5^ cells/well in quadruplicate in a black 96-well plate (3603, Falcon Corning, Kaiserslautern, Germany) and treated for 24 h or 48 h with the respective conditions mentioned in the corresponding figure legends. After treatment, the cells were incubated with 0.625 µg/well in 50 µL pHrodo™ Red *E. coli* BioParticles™ (4615, Sartorius, Göttingen, Germany) following the manufacturer’s instructions. Cellular uptake of labeled *E.coli* particles was monitored over time in an IncuCyte S3 live-cell imaging analysis system. Images were captured every hour using a 10× objective at an exposure time of 400 ms. The kinetic index of engulfed labeled *E. coli* particles was calculated by the total integrated red object intensity (RCU × µm^2^/image) in the IncuCyte software (version 2023B) by using a mask generated with the positive control cytochalasin D (1 µM for 1 h treatment; PHZ1063, Invitrogen, Darmstadt, Germany). Data were visualized using GraphPad Prism v10.1.2 (GraphPad Software, San Diego, CA, USA) and Adobe Illustrator 10 (2022, version 26.4).

### 4.10. GSH/GSSSG Assay

IDMs were seeded at a density of 0.3 × 10^5^ cells/well in quadruplicates in a white opaque 96-well plate (353296, Falcon Corning, Kaiserslautern, Germany) and treated for 24 h or 48 h with the respective conditions mentioned in the corresponding figure legends. To quantify total glutathione ratios, a luminescent -based GSH/GSSG-Glo™ Assay (V6611, Promega, Walldorf, Germany) was performed following the manufacturer’s instructions for adherent cells using the total glutathione lysis reagent. No-cell, cell-only, and vehicle (untreated, 0.1% DMSO) conditions were used as internal controls and measured in triplicate. GSH/GSSG ratios were calculated directly from luminescence measurements (Net RLU) without a standard curve. Data were normalized to the 0.1% DMSO control and visualized using GraphPad Prism v10.1.2 (GraphPad Software, San Diego, CA, USA) and Adobe Illustrator 10 (2022, version 26.4).

### 4.11. Ferroptosis Assay

IDMs were seeded at a density of 0.09 × 10^5^ cells/well in triplicates in a black 96-well plate (3603, Falcon Corning, Kaiserslautern, Germany) and treated for 24 h or 48 h with the respective conditions mentioned in the corresponding figure legends. To induce ferroptosis, 0.1 µM RSL3 (SML2234, Sigma Aldrich, Schnelldorf, Germany) was added after the treatment period in combination with CellTox^TM^ Green dye (1:5000; Promega, Walldorf, Germany, G8743) following the manufacturer’s instructions. To inhibit RSL3-induced ferroptosis, 1 µM ferrostatin-1 (F-1; SML0583, Sigma Aldrich, Schnelldorf, Germany) was added together with RSL3 and the CellTox^TM^ Green dye. To address the GCH1 inhibition, 4 mM DAHP was added (2,4-Diamino-6-hydroxypyrimidine, B21240, Thermo Fisher Scientific, Karlsruhe, Germany). Ferroptotic cell death was monitored over time in the IncuCyte S3 live-cell imaging analysis system. Images were captured on cell-by-cell adherent levels every hour using a 10× objective at an exposure time of 800 ms. The percentage of dead cells was calculated by the total integrated green object intensity (GCU × µm^2^/image) using the IncuCyte software (version 2023B) by using a mask generated with the positive control of untreated RSL3 versus RSL3+F-1-treated cells. Data were visualized using GraphPad Prism v10.1.2 (GraphPad Software, San Diego, CA, USA) and Adobe Illustrator 10 (2022, version 26.4).

### 4.12. Mitochondrial and Cytosolic ROS Assay

IDMs were seeded at a density of 0.3 × 10^5^ cells/well in quadruplicates in a 96-well plate (16303, Nunc, Schwerte, Germany) and treated for 24 h or 48 h with the respective conditions mentioned in the corresponding figure legends. To assess mitochondrial ROS levels, the MitoSOX^TM^ Red Mitochondrial Superoxide Indicator (M36008, Invitrogen, Darmstadt, Germany) was reconstituted in DMSO (12611S, CST, Frankfurt am Main, Germany) and added at a 1:1000 dilution in HBSS buffer (14025092, Gibco Thermo Fisher Scientific, Karlsruhe, Germany). IDMs were washed once with 1× PBS, followed by the addition of 100 µL/well of the prepared solution. After a 30 min incubation at 37 °C and 5% CO_2_, mitochondrial ROS levels were monitored over time in the IncuCyte S3 live-cell imaging analysis system. Images were captured on an adherent cell-by-cell level every hour using a 10× objective at an exposure time of 400 ms. The percentage of mitochondrial superoxides was calculated from the total integrated red object intensity (RCU/GCU × µm^2^/image) using the IncuCyte software (IncuCyte 2023B) by using a mask generated with the positive control staurosporine (0.05 µM 24 h treatment; S4400, Sigma Aldrich, Schnelldorf, Germany). Data were normalized to the 0.1% DMSO control (100%) and visualized using GraphPad Prism v10.1.2 (GraphPad Software, San Diego, CA, USA) and Adobe Illustrator 10 (2022, version 26.4).

IDMs were seeded at a density of 0.3 × 10^5^ cells/well in quadruplicates in a 96-well plate (16303, Nunc, Schwerte, Germany) and treated for 24 h or 48 h with the respective conditions mentioned in the corresponding figure legends. To assess total cytosolic ROS levels, the Amplex™ Red Hydrogen Peroxide/Peroxidase Assay Kit (Invitrogen, Darmstadt, Germany, A22188) was used. Following the manufacturer’s instructions, the Amplex Red reagent was reconstituted in 60 µL DMSO (12611S, CST, Frankfurt am Main, Germany) and the peroxidase vial was reconstituted in 1 mL of 1× assay buffer. The final working solution contained 50 µM Amplex Red, 0.1 U/mL horseradish peroxidase, 1 U/mL superoxide dismutase (S5395, Sigma Aldrich, Schnelldorf, Germany), 5 µg/mL digitonin (4005.1, Carl Roth, Karlsruhe, Germany) in Krebs-Ringer bicarbonate buffer (K4002, Sigma Aldrich, Schnelldorf, Germany). After a 1× PBS wash, 100 µL/well of the final working solution was added, and the plate was incubated at 37 °C for a 90 min measurement. The red fluorescence intensity (excitation/emission: 540/590 nm) was measured using a SpectraMax^®^ M-Series Multi-Mode Microplate Readers (Molecular Devices, Puchheim, Germany). Data were normalized to the baseline (0 min), represented relative to the 0.1% DMSO control (100%) and visualized using GraphPad Prism v10.1.2 (GraphPad Software, San Diego, CA, USA) and Adobe Illustrator 10 (2022, version 26.4).

### 4.13. Immunoblotting

IDMs were seeded at a density of 0.8 × 10^6^ cells/well in a 6-well plate (140685 Thermo Fisher Scientific, Karlsruhe, Germany). iPSC-derived microglia (iCell^®^ Microglia, FUJIFILM Cellular Dynamics, Madison, WI, USA) were seeded at a density of 0.8*10^6^ cells/well in a PDL-coated 6-well plate (657940, Greiner, Frickenhausen, Germany). Both cell types were treated for 24 h with the respective conditions mentioned in the corresponding figure legends. Cells (IDMs line 201B7 or iPSC-derived microglia iCell^®^ Microglia) were lysed in RIPA lysis buffer (R0278, Sigma Aldrich, Schnelldorf, Germany) supplemented with a protease and phosphatase inhibitor cocktail (100×; 78440, Thermo Fisher Scientific, Karlsruhe, Germany). Protein concentrations were determined using the Pierce™ BCA Protein Assay Kit (A55864, Thermo Fisher Scientific, Karlsruhe, Germany) according to the manufacturer’s instructions and absorbance was measured at 562 nm using a SpectraMax^®^ M-Series Multi-Mode Microplate Readers (Molecular Devices, Puchheim, Germany). Cell lysates were separated on Novex™ WedgeWell™ 4 bis 12%, Tris-Glycin, 1,0 mm, Mini-Protein-Gele (NW04125BOX, Invitrogen, Darmstadt, Germany) using a Bio-Rad Precision Plus markers (161-0394, Bio-Rad Laboratories, Feldkirchen, Germany) in a Bio-Rad Mini PROTEAN Tetra System (Bio-Rad Laboratories, Feldkirchen, Germany). Following transfer to nitrocellulose membranes (0.2 μm, 10600001, Amersham, Freiburg im Breisgau, Germany) and the membranes were blocked in 5% milk (42590.01, SERVA, Heidelberg, Germany) in Tris-buffered saline containing 0.1% Tween-20 (TBS-T; 9997, CST, Frankfurt am Main, Germany) for 2 h. The membranes were cut according to the molecular weight of the target proteins. Membranes were incubated overnight at 4 °C with the following primary antibodies in blocking buffer: GRB2 (1:1000; 610112, Becton Dickinson, Heidelberg, Germany), ARK5 (E4T2A) (1:500; 47677, CST, Frankfurt am Main, Germany) and SNARK/NUAK2 (E4V4Q) (1:500; 15452, CST, Frankfurt am Main, Germany). Membranes were washed in 1×TBS-T and water before incubation with horseradish peroxidase–conjugated secondary antibodies (goat anti-rabbit (1:2500; RPN4301, Amersham, Freiburg im Breisgau, Germany) and goat anti-mouse (1:1500; A8924, Sigma Aldrich, Schnelldorf, Germany)) for 1 h at room temperature before visualization with SuperSignal™ West Femto Maximum Sensitivity Substrat (34096; Thermo Fisher Scientific, Karlsruhe, Germany). Visualization of the protein was monitored using an Odyssey Fc Imaging System (LI-COR Biosciences, Bad Homburg vor der Höhe, Germany). Image analysis was performed using the LI-COR Acquisition Software (version 2.4, Bad Homburg vor der Höhe, Germany). GRB2 was used as a loading control. Data were visualized using Adobe Illustrator 10 (2022, version 26.4).

### 4.14. Gene Expression

IDMs were seeded at a density of 0.5 × 10^5^ cells/well in a 12-well plate (150628, Thermo Fisher Scientific, Karlsruhe, Germany) and treated for 24 h or 48 h with the respective conditions in triplicate, as mentioned in the corresponding figure legends. After treatment, the cells were lysed using RLT Plus buffer (1053393, Qiagen, Hilden, Germany), and total RNA was extracted using the RNeasy Plus Mini Kit (74136, Qiagen, Hilden, Germany), following the manufacturer’s instructions. In the final step, RNA was eluted in RNase-free water and quantified at absorbance 260/280 nm using a NanoDrop spectrophotometer (NanoDrop LitePlusUV, Thermo Fisher Scientific, Karlsruhe, Germany). Complementary DNA was synthesized using the High-Capacity cDNA Reverse Transcription Kit (4368813, Thermo Fisher Scientific, Karlsruhe, Germany). Quantitative PCR was conducted on a QuantStudio 6 Flex system (Thermo Fisher Scientific, Karlsruhe, Germany) using TaqMan gene expression assay (Applied Biosystems Assay ID: *POLR2A* (HS00172187_m1), *GCH1* (Hs00609198_m1), *GPX4* (Hs00157812_m1), *IL4I1* (Hs00541746_m1), *SLC7A11* (Hs00921938_m1) and *IDO1* (Hs00984148_m1)). Relative gene expression was calculated using the ΔΔCq method. Data were normalized to PO*LR2A* to untreated control. Data were visualized using GraphPad Prism v10.1.2 (GraphPad Software, San Diego, CA, USA) and Adobe Illustrator 10 (2022, version 26.4).

### 4.15. ATP Rate and Mitochondrial Function Assay

Total ATP production rate in IDMs was quantified using the Agilent Seahorse XF Real-Time ATP Rate Assay (103592-100, Agilent Technologies, Frankfurt am Main, Germany). To determine the mitochondrial function of IDMs, the Agilent Seahorse XF Cell Mito Stress Test Assay (103015-100, Agilent Technologies, Frankfurt am Main, Germany) was used. Both assays were performed in accordance with the manufacturer’s instructions. IDMs at a density of 0.3 × 10^5^ cells/well were seeded in a XFe96/XF Pro Cell Culture Microplate (103794-100, Agilent Technologies, Frankfurt am Main, Germany) and treated for 24 h or 48 h with the respective conditions in quadruplicate as mentioned in the corresponding figure legends. Prior to the assay, IDMs were washed once with PBS and incubated for 30 min at 37 °C in a non-CO_2_ incubator with Seahorse XF assay medium (Agilent Technologies, Frankfurt am Main, Germany 103680-100), consisting of non-buffered DMEM supplemented with 10 mM glucose, 1 mM pyruvate, and 2 mM glutamine. To interrogate mitochondrial function, the following cpds (1.5 µM oligomycin, 2 µM FCCP and 0.5 µM Rot/AA) were sequentially injected during the assay procedure. Data were recorded in real time, normalized to protein content as determined by the Pierce™ BCA Protein Assay Kit (Thermo Fisher Scientific, Karlsruhe, Germany, A55864), analyzed using the Agilent Seahorse Analytics software interface (version 2.6.4, seahorseanalytics.agilent.com., Agilent Technologies) and visualized using GraphPad Prism v10.1.2 (GraphPad Software, San Diego, CA, USA) and Adobe Illustrator 10 (2022, version 26.4).

### 4.16. Statistical Analysis

Data processing, visualization, and statistical analyses were performed using GraphPad Prism v10.1.2 (GraphPad Software, San Diego, CA, USA). All quantitative data are reported as mean ± standard deviation (SD) or mean ± standard error of the mean (SEM) of biological replicates or screen replicates, with specific sample sizes (*n*) and experimental setups detailed in the respective figure legends. For continuous variables with multiple treatment groups, statistical significance was evaluated using one-way or two-way analysis of variance (ANOVA), as appropriate. To account for multiple comparisons, Dunnett’s post hoc test was applied when comparing multiple treatment arms against a single vehicle or control group, whereas Tukey’s post hoc test was utilized for pairwise comparisons across all experimental conditions. For direct comparisons between two specific groups, two-tailed unpaired (t)-tests were deployed. For correlation analyses of cellular profiling features, Pearson correlation coefficients were calculated. Statistical significance is indicated across all figures as follows: * *p* < 0.05, ** *p* < 0.01, *** *p* < 0.001, **** *p* < 0.0001; ns denotes non-significant differences (*p* ≥ 0.05).

### 4.17. Figure Visualization

All schematics were created with BioRender.com and Adobe Illustrator 10 (2022 version, 26.4). Data were visualized using GraphPad Prism v10.1.2 (GraphPad Software, San Diego, CA, USA) and Adobe Illustrator 10 (2022, version 26.4).

## 5. Conclusions

In this study, we applied an ML-based phenotypic high-content cell painting screen in human IDMs and MDMs from four donors, identifying 26 MΦ-polarizing compounds (19 M2-like and 7 M1-like) from a total of 540 compounds screened across two annotated libraries (opnMe and a subset of JUMP-CP). The M1- and M2-like phenotypic responses were quantified using complementary imaging-based read-outs, including cell roundness and cell painting feature profiling, as previously described, following toxicity assessment in dose–response studies.

Among the identified hits, the pan-AMPK activator BI-9774 induced an elongated, M2-like phenotype, whereas inhibition of the AMPK-related kinases NUAK1/2 by WZ4003 promoted a rounded, M1-like state. These opposing phenotypes were accompanied by opposing effects on phagocytosis and efferocytosis in IDMs. In addition, mitochondrial cell painting profiling indicated that NUAK1/2 inhibition shares a similar profile with Rot/AA treatment. Furthermore, pharmacological NUAK1/2 inhibition was associated with altered mitochondrial function, an adaptive antioxidant response, and protection against RSL3-induced ferroptosis.

Collectively, our study highlights the utility of phenotypic cell painting approaches to link MΦ morphology, cellular state, and functional outcomes. Moreover, it indicates the identification of WZ4003 as modulator of MΦ (re-)polarization.

## Data Availability

The imaging data presented in this study are available in SImA3.1. You receive access to the imaging raw data by email to the corresponding author. Feature extracted data from the primary and confirmation screen can be found in the Supplementary Information Files S3 and S4.

## Supplementary Materials

The following supporting information can be downloaded at: https://www.mdpi.com/article/10.3390/ijms27167279/s1.
