# Supplementary material for: Identification of a NUAK1/2 Inhibitor as a Macrophage-Polarizing Compound by a Machine Learning-Based Phenotypic Cell Painting Screen
Source: Int J Mol Sci. 2026 Aug 14;27(16):7279. doi: 10.3390/ijms27167279 (PMC13513706; doi:10.3390/ijms27167279)
Supplement: Supplementary file 1 [file ijms-27-07279-s001.zip › S2_linear_classifier_data.pdf]

linear_classifier_data_model_2

Jakob Dittmer

load packages

```
library(tidyverse)
```

```
## -- Attaching core tidyverse packages ------------------------ tidyverse 2.0.0 --
## v dplyr     1.1.4     v readr     2.1.6
## v forcats   1.0.1     v stringr   1.5.2
## v ggplot2   4.0.1     v tibble    3.3.1
## v lubridate 1.9.4     v tidyr     1.3.1
## v purrr     1.1.0
## -- Conflicts ------------------------------------------ tidyverse_conflicts() --
## x dplyr::filter() masks stats::filter()
## x dplyr::lag()    masks stats::lag()
## i Use the conflicted package (<http://conflicted.r-lib.org/>) to force all conflicts to become errors
```

```
library(readxl)
library(ggrepel)
library(tidymodels)
```

```
## -- Attaching packages -------------------------------------- tidymodels 1.4.1 --
## v broom        1.0.10     v rsample      1.3.1
## v dials        1.4.2      v tailor       0.1.0
## v infer        1.0.9      v tune         2.0.0
## v modeldata    1.5.1      v workflows    1.3.0
## v parsnip      1.3.3      v workflowsets 1.1.1
## v recipes      1.3.1      v yardstick    1.3.2
## -- Conflicts ----------------------------------------- tidymodels_conflicts() --
## x scales::discard() masks purrr::discard()
## x dplyr::filter()   masks stats::filter()
## x recipes::fixed()  masks stringr::fixed()
## x dplyr::lag()      masks stats::lag()
## x yardstick::spec() masks readr::spec()
## x recipes::step()   masks stats::step()
```

```
library(writexl)
library(scales)
```

import metadata

```
##import the plate layouts and pivot to tidy format
df_layout_1 <- readxl::read_xlsx("import/efferocytosis_data/layout_efferocytosis.xlsx",
                                 skip = 12, n_max = 16) %>%
  pivot_longer(cols = 2:ncol(.), names_to = "Column", values_to = "Treatment") %>%
  rename(Row = ...1) %>%
  unite("Well_ID", Row, Column, sep = "", remove = FALSE) %>%
```

```
  mutate(`library` = case_when(
    between(as.numeric(Column), 1, 4) ~ "ctrl",
    between(as.numeric(Column), 6, 23) ~ "OpnMe",
    TRUE ~ NA),
    sourcefile = "JB0000390",
    incubation = "24h",
    celltype = "201B7 IDM")
```

```
## New names:
## * `` -> `...1`
```

```
df_layout_1_2 <- readxl::read_xlsx("import/efferocytosis_data/layout_efferocytosis.xlsx",
                                   skip = 12, n_max = 16) %>%
  pivot_longer(cols = 2:ncol(.), names_to = "Column", values_to = "Treatment") %>%
  rename(Row = ...1) %>%
  unite("Well_ID", Row, Column, sep = "", remove = FALSE) %>%
  mutate(`library` = case_when(
    between(as.numeric(Column), 1, 4) ~ "ctrl",
    between(as.numeric(Column), 6, 23) ~ "OpnMe",
    TRUE ~ NA),
    sourcefile = "JB0000394",
    incubation = "24h",
    celltype = "MDM 414")
```

```
## New names:
## * `` -> `...1`
```

```
df_layout_2 <- readxl::read_xlsx("import/efferocytosis_data/layout_efferocytosis.xlsx",
                                 skip = 31, n_max = 16) %>%
  pivot_longer(cols = 2:ncol(.), names_to = "Column", values_to = "Treatment") %>%
  rename(Row = ...1) %>%
  unite("Well_ID", Row, Column, sep = "", remove = FALSE) %>%
  mutate(`library` = case_when(
    between(as.numeric(Column), 1, 4) ~ "ctrl",
    between(as.numeric(Column), 13, 23) ~ "JumpCP",
    between(as.numeric(Column), 6, 12) ~ "OpnMe",
    TRUE ~ NA),
    sourcefile = "JB0000391",
    incubation = "24h",
    celltype = "201B7 IDM") %>%
  mutate(`library` = case_when(
    Well_ID %in% c("I12", "J12", "K12", "L12", "M12", "N12", "O12", "P12") ~ "JumpCP",
    TRUE ~ `library`))
```

```
## New names:
## * `` -> `...1`
```

```
df_layout_2_2 <- readxl::read_xlsx("import/efferocytosis_data/layout_efferocytosis.xlsx",
                                   skip = 31, n_max = 16) %>%
  pivot_longer(cols = 2:ncol(.), names_to = "Column", values_to = "Treatment") %>%
  rename(Row = ...1) %>%
  unite("Well_ID", Row, Column, sep = "", remove = FALSE) %>%
  mutate(`library` = case_when(
    between(as.numeric(Column), 1, 4) ~ "ctrl",
    between(as.numeric(Column), 13, 23) ~ "JumpCP",
```

```
    between(as.numeric(Column), 6, 12) ~ "OpnMe",
    TRUE ~ NA),
    sourcefile = "JB0000395",
    incubation = "24h",
    celltype = "MDM 414") %>%
  mutate(`library` = case_when(
    Well_ID %in% c("I12", "J12", "K12", "L12", "M12", "N12", "O12", "P12") ~ "JumpCP",
    TRUE ~ `library`))
```

```
## New names:
## * `` -> `...1`
```

```
df_layout_3 <- readxl::read_xlsx("import/efferocytosis_data/layout_efferocytosis.xlsx",
                                 skip = 50, n_max = 16) %>%
  pivot_longer(cols = 2:ncol(.), names_to = "Column", values_to = "Treatment") %>%
  rename(Row = ...1) %>%
  unite("Well_ID", Row, Column, sep = "", remove = FALSE) %>%
  mutate(`library` = case_when(
    between(as.numeric(Column), 1, 4) ~ "ctrl",
    between(as.numeric(Column), 6, 23) ~ "JumpCP",
    TRUE ~ NA),
    sourcefile = "JB0000389",
    incubation = "24h",
    celltype = "201B7 IDM")
```

```
## New names:
## * `` -> `...1`
```

```
df_layout_3_2 <- readxl::read_xlsx("import/efferocytosis_data/layout_efferocytosis.xlsx",
                                   skip = 50, n_max = 16) %>%
  pivot_longer(cols = 2:ncol(.), names_to = "Column", values_to = "Treatment") %>%
  rename(Row = ...1) %>%
  unite("Well_ID", Row, Column, sep = "", remove = FALSE) %>%
  mutate(`library` = case_when(
    between(as.numeric(Column), 1, 4) ~ "ctrl",
    between(as.numeric(Column), 6, 23) ~ "JumpCP",
    TRUE ~ NA),
    sourcefile = "JB0000396",
    incubation = "24h",
    celltype = "MDM 414")
```

```
## New names:
## * `` -> `...1`
```

```
df_layout_4 <- readxl::read_xlsx("import/efferocytosis_data/layout_efferocytosis.xlsx",
                                 skip = 70, n_max = 16) %>%
  pivot_longer(cols = 2:ncol(.), names_to = "Column", values_to = "Treatment") %>%
  rename(Row = ...1) %>%
  unite("Well_ID", Row, Column, sep = "", remove = FALSE) %>%
  mutate(`library` = case_when(
    between(as.numeric(Column), 1, 4) ~ "ctrl",
    between(as.numeric(Column), 7, 8) ~ "JumpCP",
    TRUE ~ NA),
    sourcefile = "JB0000392",
    incubation = "24h",
```

```
    celltype = "201B7 IDM")
```

```
## New names:
## * `` -> `...1`
```

```
df_layout_4_2 <- readxl::read_xlsx("import/efferocytosis_data/layout_efferocytosis.xlsx",
                                   skip = 70, n_max = 16) %>%
  pivot_longer(cols = 2:ncol(.), names_to = "Column", values_to = "Treatment") %>%
  rename(Row = ...1) %>%
  unite("Well_ID", Row, Column, sep = "", remove = FALSE) %>%
  mutate(`library` = case_when(
    between(as.numeric(Column), 1, 4) ~ "ctrl",
    between(as.numeric(Column), 7, 8) ~ "JumpCP",
    TRUE ~ NA),
    sourcefile = "JB0000393",
    incubation = "24h",
    celltype = "MDM 414")
```

```
## New names:
## * `` -> `...1`
```

```
df_layout_complete <-
  bind_rows(
    df_layout_1,
    df_layout_1_2,
    df_layout_2,
    df_layout_2_2,
    df_layout_3,
    df_layout_3_2,
    df_layout_4,
    df_layout_4_2) %>%
  select(incubation, Treatment, Well_ID, sourcefile, celltype, everything())
```

import data

```
#import rawdata files

#set directory for model 2 new data
directory_model_2 <- "import/efferocytosis_data/model_2_389-396"

#list of files
files_model_2 <- list.files(path = directory_model_2, full.names = TRUE)

#import data and drop unneeded variables
df_data_model_2 <- files_model_2 %>%
  map_df(~mutate(read_tsv(.), sourcefile = tools::file_path_sans_ext(basename(.))))
```

```
## Rows: 368 Columns: 1291
## -- Column specification --------------------------------------------------------
## Delimiter: "\t"
## chr    (2): WellName, Well URL
## dbl (1289): Row, Col, Cells Selected - Number of Objects, Cells Selected - I...
##
## i Use `spec()` to retrieve the full column specification for this data.
## i Specify the column types or set `show_col_types = FALSE` to quiet this message.
```

```
## Rows: 368 Columns: 1291
## -- Column specification --------------------------------------------------------
## Delimiter: "\t"
## chr    (2): WellName, Well URL
## dbl (1289): Row, Col, Cells Selected - Number of Objects, Cells Selected - I...
##
## i Use `spec()` to retrieve the full column specification for this data.
## i Specify the column types or set `show_col_types = FALSE` to quiet this message.
## Rows: 368 Columns: 1291
## -- Column specification --------------------------------------------------------
## Delimiter: "\t"
## chr    (2): WellName, Well URL
## dbl (1289): Row, Col, Cells Selected - Number of Objects, Cells Selected - I...
##
## i Use `spec()` to retrieve the full column specification for this data.
## i Specify the column types or set `show_col_types = FALSE` to quiet this message.
## Rows: 368 Columns: 1291
## -- Column specification --------------------------------------------------------
## Delimiter: "\t"
## chr    (2): WellName, Well URL
## dbl (1289): Row, Col, Cells Selected - Number of Objects, Cells Selected - I...
##
## i Use `spec()` to retrieve the full column specification for this data.
## i Specify the column types or set `show_col_types = FALSE` to quiet this message.
## Rows: 368 Columns: 1291
## -- Column specification --------------------------------------------------------
## Delimiter: "\t"
## chr    (2): WellName, Well URL
## dbl (1289): Row, Col, Cells Selected - Number of Objects, Cells Selected - I...
##
## i Use `spec()` to retrieve the full column specification for this data.
## i Specify the column types or set `show_col_types = FALSE` to quiet this message.
## Rows: 368 Columns: 1291
## -- Column specification --------------------------------------------------------
## Delimiter: "\t"
## chr    (2): WellName, Well URL
## dbl (1289): Row, Col, Cells Selected - Number of Objects, Cells Selected - I...
##
## i Use `spec()` to retrieve the full column specification for this data.
## i Specify the column types or set `show_col_types = FALSE` to quiet this message.
## Rows: 368 Columns: 1291
## -- Column specification --------------------------------------------------------
## Delimiter: "\t"
## chr    (2): WellName, Well URL
## dbl (1289): Row, Col, Cells Selected - Number of Objects, Cells Selected - I...
##
## i Use `spec()` to retrieve the full column specification for this data.
## i Specify the column types or set `show_col_types = FALSE` to quiet this message.
## Rows: 368 Columns: 1291
## -- Column specification --------------------------------------------------------
## Delimiter: "\t"
## chr    (2): WellName, Well URL
## dbl (1289): Row, Col, Cells Selected - Number of Objects, Cells Selected - I...
##
```

```
## i Use `spec()` to retrieve the full column specification for this data.
## i Specify the column types or set `show_col_types = FALSE` to quiet this message.
```

```
df_data_model_2 <- df_data_model_2 %>%
  select(
    -`Number of Analyzed Fields`,
    -`Well URL`,
    -Row,
    -Col,
    -`M0 - Number of Objects`,
    -`M1 - Number of Objects`,
    -`M2 - Number of Objects`
  ) %>%
  rename(Well_ID = WellName) %>%
  select(sourcefile, Well_ID, `%M0`, `%M1`, `%M2`, everything()) %>%
  mutate(sourcefile = str_extract(sourcefile, "^[^_]*"))
```

label with metadata

```
df_data_model_2_annotated <- df_data_model_2 %>%
  left_join(df_layout_complete, by = c("Well_ID", "sourcefile")) %>%
  filter(!is.na(Treatment)) %>% #remove empty wells
  select(incubation,celltype, Treatment, everything())
```

normalize and aggregate data

```
#create a dataset that has values normalized to DMSO ctrl
df_ctrls_new <- df_data_model_2_annotated %>%
  filter(Treatment == "DMSO 0.1%") %>%
  select(sourcefile, `Cells Selected - Number of Objects`,
         `%M1`, `%M2`) %>%
  group_by(sourcefile) %>%
  summarise(`Cells Selected - Number of Objects` = mean(`Cells Selected - Number of Objects`),
            `%M1` = mean(`%M1`),
            `%M2` = mean(`%M2`))

df_data_model_2_annotated <- df_data_model_2_annotated %>%
  left_join(df_ctrls_new,
            by = "sourcefile",
            suffix = c("", "_ctrl")) %>%
  mutate(`%M2 - %M1 normalized` = (`%M2` - `%M2_ctrl`) - (`%M1` - `%M1_ctrl`),
         `Cell number relative to DMSO ctrl` =
           `Cells Selected - Number of Objects` / `Cells Selected - Number of Objects_ctrl`) %>%
  select(`%M2 - %M1 normalized`,
         ends_with("ctrl"),everything())

#aggregate data grouped per treatment, celltype and incubation time level
df_data_model_2_aggregated <- df_data_model_2_annotated %>%
  select(-Well_ID) %>%
  group_by(
    celltype,
    Treatment,
    incubation
```

```
  ) %>%
  summarise(across(everything(), mean, na.rm = TRUE)) %>%
  ungroup()
```

```
## Warning: There were 1905 warnings in `summarise()`.
## The first warning was:
## i In argument: `across(everything(), mean, na.rm = TRUE)`.
## i In group 1: `celltype = "201B7 IDM"`, `Treatment = "Alsterpaullone 1µM"`,
##   `incubation = "24h"`.
## Caused by warning:
## ! The `...` argument of `across()` is deprecated as of dplyr 1.1.0.
## Supply arguments directly to `.fns` through an anonymous function instead.
##
##   # Previously
##   across(a:b, mean, na.rm = TRUE)
##
##   # Now
##   across(a:b, \(x) mean(x, na.rm = TRUE))
## i Run `dplyr::last_dplyr_warnings()` to see the 1904 remaining warnings.

## `summarise()` has grouped output by 'celltype', 'Treatment'. You can override
## using the `.groups` argument.
```

clean data before performing PCA

```
#identify columns with wissing values
missing_value_features <- df_data_model_2_aggregated %>%
  summarise(across(everything(), ~sum(is.na(.)))) %>%
  pivot_longer(1:ncol(.), names_to = "column name", values_to = "number of missing values") %>%
  arrange(desc(`number of missing values`)) %>%
  filter(`number of missing values` > 0) %>%
  pull(`column name`)

#clean dataset
df_data_model_2_aggregated <- df_data_model_2_aggregated %>%
  select(!all_of(missing_value_features))

#split into IDM and MDM datasets
df_data_aggregated_model_2_IDM <- df_data_model_2_aggregated %>%
  filter(celltype == "201B7 IDM")

df_data_aggregated_model_2_MDM <- df_data_model_2_aggregated %>%
  filter(celltype == "MDM 414")
```

normalize data and calculate PCAs for each celltype

```
#split datasets into features and metadate and perform PCA for the features
df_data_PCA_IDM <- df_data_aggregated_model_2_IDM %>%
  select(!1:12) %>% #separate metadata
  recipe( ~ ., data = .) %>%
  step_normalize(all_numeric_predictors()) %>% #scale and center (subtract mean and devide by sd)
  step_nzv(all_numeric_predictors()) %>% #remove near zero variance features (none affected)
  step_corr(all_numeric_predictors(), threshold = 0.99) %>% #decorrelate (99% correlation threshold)
```

```
  step_pca(all_numeric_predictors(), num_comp = 2) %>% #calculate first 2 PCs
  prep() %>%
  juice() %>%
  bind_cols(select(df_data_aggregated_model_2_IDM, 1:12)) #reattach metadata

df_data_PCA_MDM <- df_data_aggregated_model_2_MDM %>%
  select(!1:12) %>% #separate metadata
  recipe( ~ ., data = .) %>%
  step_normalize(all_numeric_predictors()) %>% #scale and center (subtract mean and devide by sd)
  step_nzv(all_numeric_predictors()) %>% #remove near zero variance features (none affected)
  step_corr(all_numeric_predictors(), threshold = 0.99) %>% #decorrelate (99% correlation threshold)
  step_pca(all_numeric_predictors(), num_comp = 2) %>% #calculate first 2 PCs
  prep() %>%
  juice() %>%
  bind_cols(select(df_data_aggregated_model_2_MDM, 1:12)) #reattach metadata
```

export data

```
bind_rows(df_data_PCA_IDM, df_data_PCA_MDM) %>%
  write_xlsx("figures/secondary_screen_data_model_2.xlsx")
```

isolate mitochondira features, normalize data and calculate PCAs for each celltype

```
#split datasets into features and metadata and perform PCA for the features
df_data_PCA_IDM_mito <- df_data_aggregated_model_2_IDM %>%
  select(!1:12) %>% #separate metadata
  select(contains("647")) %>% #select mitochondria channel data
  recipe( ~ ., data = .) %>%
  step_normalize(all_numeric_predictors()) %>% #scale and center (subtract mean and devide by sd)
  step_nzv(all_numeric_predictors()) %>% #remove near zero variance features (none affected)
  step_corr(all_numeric_predictors(), threshold = 0.99) %>% #decorrelate (99% correlation threshold)
  step_pca(all_numeric_predictors(), num_comp = 2) %>% #calculate first 2 PCs
  prep() %>%
  juice() %>%
  bind_cols(select(df_data_aggregated_model_2_IDM, 1:12)) #reattach metadata

df_data_PCA_MDM_mito <- df_data_aggregated_model_2_MDM %>%
  select(!1:12) %>% #separate metadata
  select(contains("647")) %>% #select mitochondria channel data
  recipe( ~ ., data = .) %>%
  step_normalize(all_numeric_predictors()) %>% #scale and center (subtract mean and devide by sd)
  step_nzv(all_numeric_predictors()) %>% #remove near zero variance features (none affected)
  step_corr(all_numeric_predictors(), threshold = 0.99) %>% #decorrelate (99% correlation threshold)
  step_pca(all_numeric_predictors(), num_comp = 2) %>% #calculate first 2 PCs
  prep() %>%
  juice() %>%
  bind_cols(select(df_data_aggregated_model_2_MDM, 1:12)) #reattach metadata
```

export data

```
bind_rows(df_data_PCA_IDM_mito, df_data_PCA_MDM_mito) %>%
  write_xlsx("figures/secondary_screen_data_model_2_mito.xlsx")
```
