# Supplementary material for: Identification of a NUAK1/2 Inhibitor as a Macrophage-Polarizing Compound by a Machine Learning-Based Phenotypic Cell Painting Screen
Source: Int J Mol Sci. 2026 Aug 14;27(16):7279. doi: 10.3390/ijms27167279 (PMC13513706; doi:10.3390/ijms27167279)
Supplement: Supplementary file 1 [file ijms-27-07279-s001.zip › SUPPLEMENTARY INFORMATION_JB.pdf]

Brüggenthies-Brunner et al.

Identification of a NUAK1/2 inhibitor as a macrophage-polarizing compound by a machine learning-based phenotypic cell painting screen

SUPPLEMENTARY INFORMATION

Supplementary Figure

A

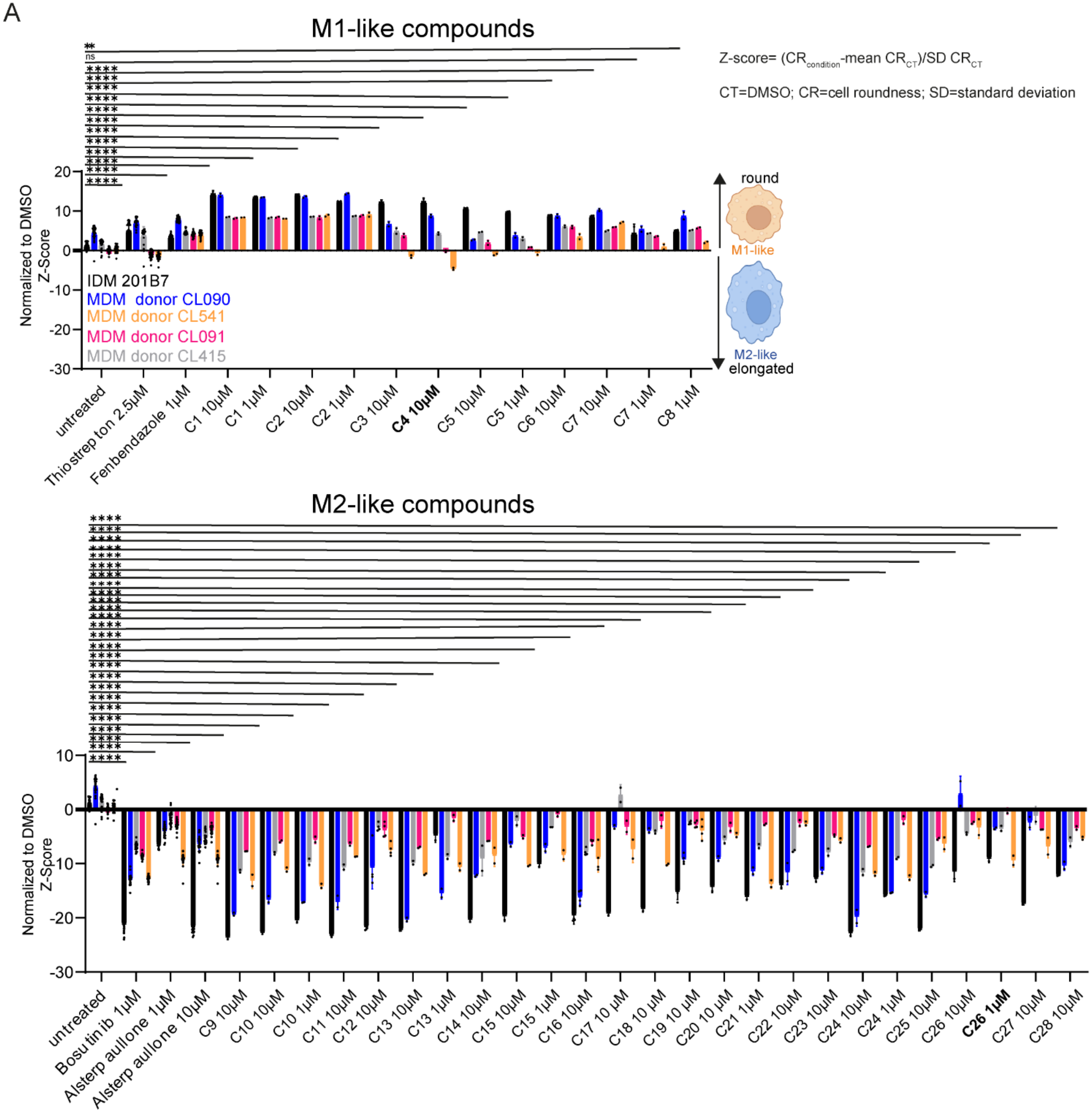

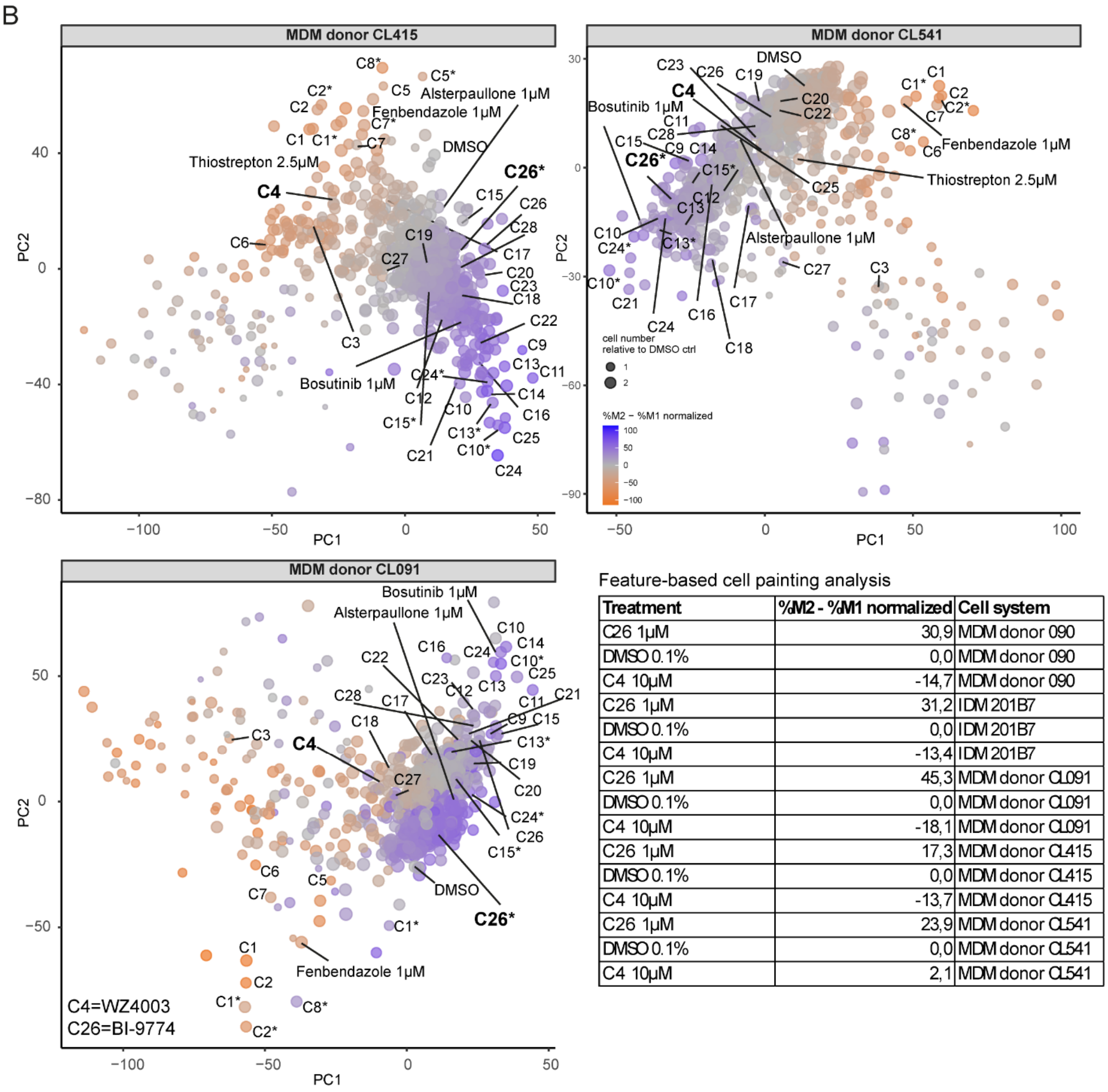

Treatment	%M2 - %M1 normalized	Cell system
C26 1µM	30,9	MDM donor 090
DMSO 0.1%	0,0	MDM donor 090
C4 10µM	-14,7	MDM donor 090
C26 1µM	31,2	IDM 201B7
DMSO 0.1%	0,0	IDM 201B7
C4 10µM	-13,4	IDM 201B7
C26 1µM	45,3	MDM donor CL091
DMSO 0.1%	0,0	MDM donor CL091
C4 10µM	-18,1	MDM donor CL091
C26 1µM	17,3	MDM donor CL415
DMSO 0.1%	0,0	MDM donor CL415
C4 10µM	-13,7	MDM donor CL415
C26 1µM	23,9	MDM donor CL541
DMSO 0.1%	0,0	MDM donor CL541
C4 10µM	2,1	MDM donor CL541

Figure S1. **(A)** Mathematical equation and quantitative Z-score calculations for cell roundness analysis of the primary screen hits, categorized into M1- and M2-like cpds. DMSO-normalized Z-scores were determined from 20x magnified images and are depicted as mean ± SD across the utilized MΦ models, including IDMs (201B7 line) and four distinct MDM donors (CL090, CL541, CL091, and CL415). Cells were treated with the indicated conditions (C = cpd; see Table S1 for cpd annotations) for 24 h. Hit selection thresholds were defined based on the internal M1- and M2-like control cpds (bosutinib 1 µM, alsterpaullone 1 and 10 µM, thiostrepton 2.5 µM, and fenbendazole 1 µM). Each library-cpd condition consisted of 2 wells per plate with 5 fields imaged per well, replicated across two 384-well plates (totaling $n = 2$ wells per condition). Other control conditions consisted of multiple wells per plate with 5 fields imaged per well, replicated across 384-well plates (totaling $n = 24$ wells per condition). Statistical analysis was performed using a two-way ANOVA followed by Dunnett's multiple comparisons test

against the vehicle control (DMSO 0.1%); asterisks denote statistical significance (**p < 0.01, ****p < 0.0001, ns = non-significant) illustrated for IDMs. **(B)** Principal component analysis (PCA) of the primary screen hits determined by cell painting feature profiling along the PC1 and PC2 axes. Profiling was executed utilizing a customized cell painting image analysis pipeline combined with a linear classifier based on 1,279 extracted features. DMSO-normalized %M2 − %M1 values represent the mean of two screen replicates (*n* = 2 wells per condition, 5 fields imaged per well at 20x magnification) and are depicted for three representative MDM donors (CL541, CL415, and CL091). Other control conditions consisted of multiple wells per plate with 5 fields imaged per well, replicated across 384-well plates (totaling *n* = 24 wells per condition). The absolute cell count relative to the vehicle (DMSO) control is represented proportionally by the diameter of the data points. Exact DMSO-normalized %M2 − %M1 source values for the screen hits C4 (WZ4003) and C26 (BI-9774) across the different cell models are provided in the embedded data table. Treatment conditions and cpd concentrations are identical to those described in panel A. Exact DMSO-normalized %M2 − %M1 values for all cell models can be found in Table S3.

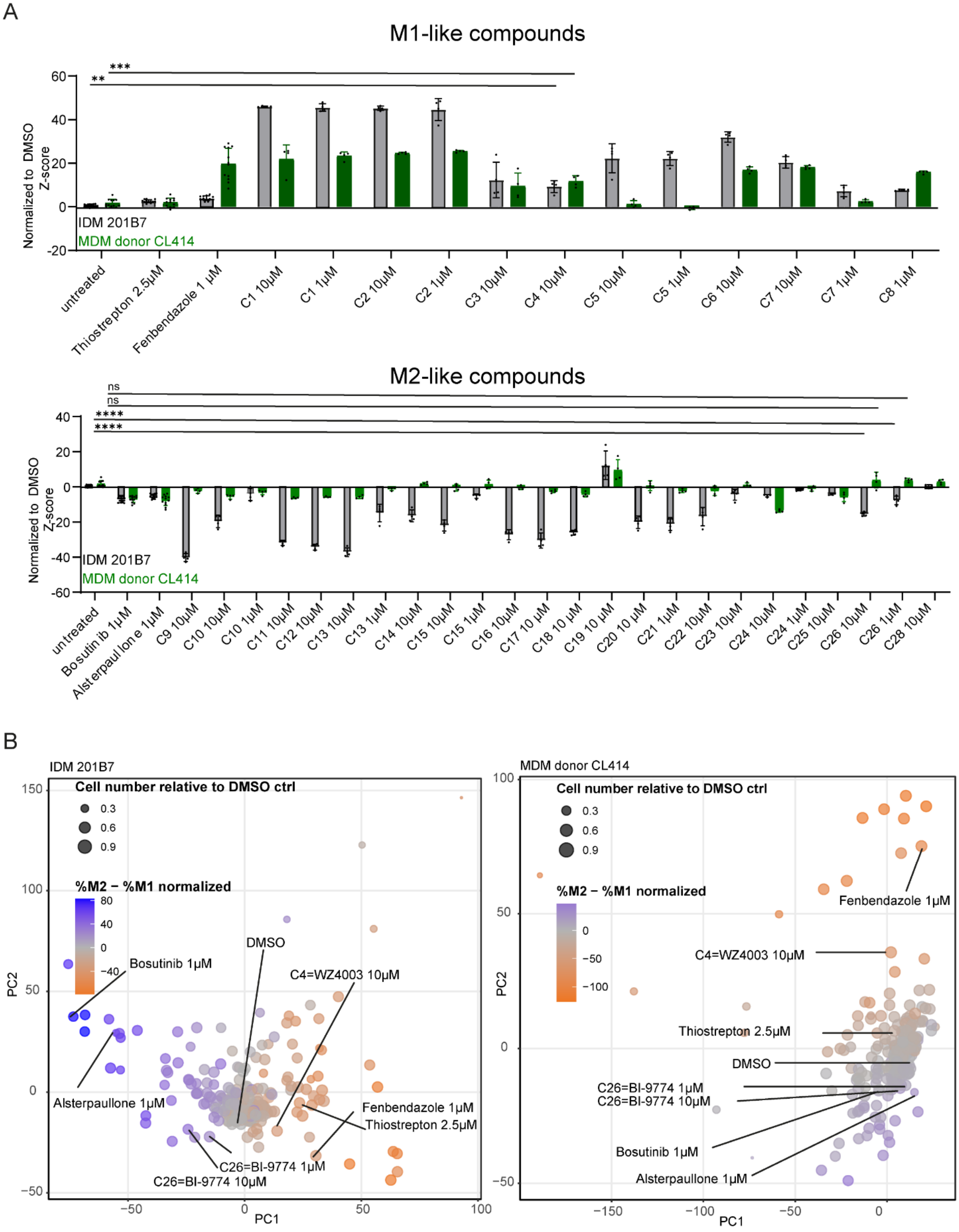
A
M1-like compounds
Normalized to DMSO Z-score
IDM 201B7
MDM donor CL414
untreated
Thiostrepton 2.5µM
Fenbendazole 1 µM
C1 10µM
C1 1µM
C2 10µM
C2 1µM
C3 10µM
C4 10µM
C5 10µM
C5 1µM
C6 10µM
C7 10µM
C7 1µM
C8 1µM
M2-like compounds
untreated
Bosutinib 1µM
Alsterpaullone 1µM
B
IDM 201B7
MDM donor CL414
Cell number relative to DMSO ctrl
%M2 − %M1 normalized
Bosutinib 1µM
DMSO
C4=WZ4003 10µM
Alsterpaullone 1µM
Fenbendazole 1µM
Thiostrepton 2.5µM
C26=BI-9774 1µM
C26=BI-9774 10µM
PC1
PC2

C

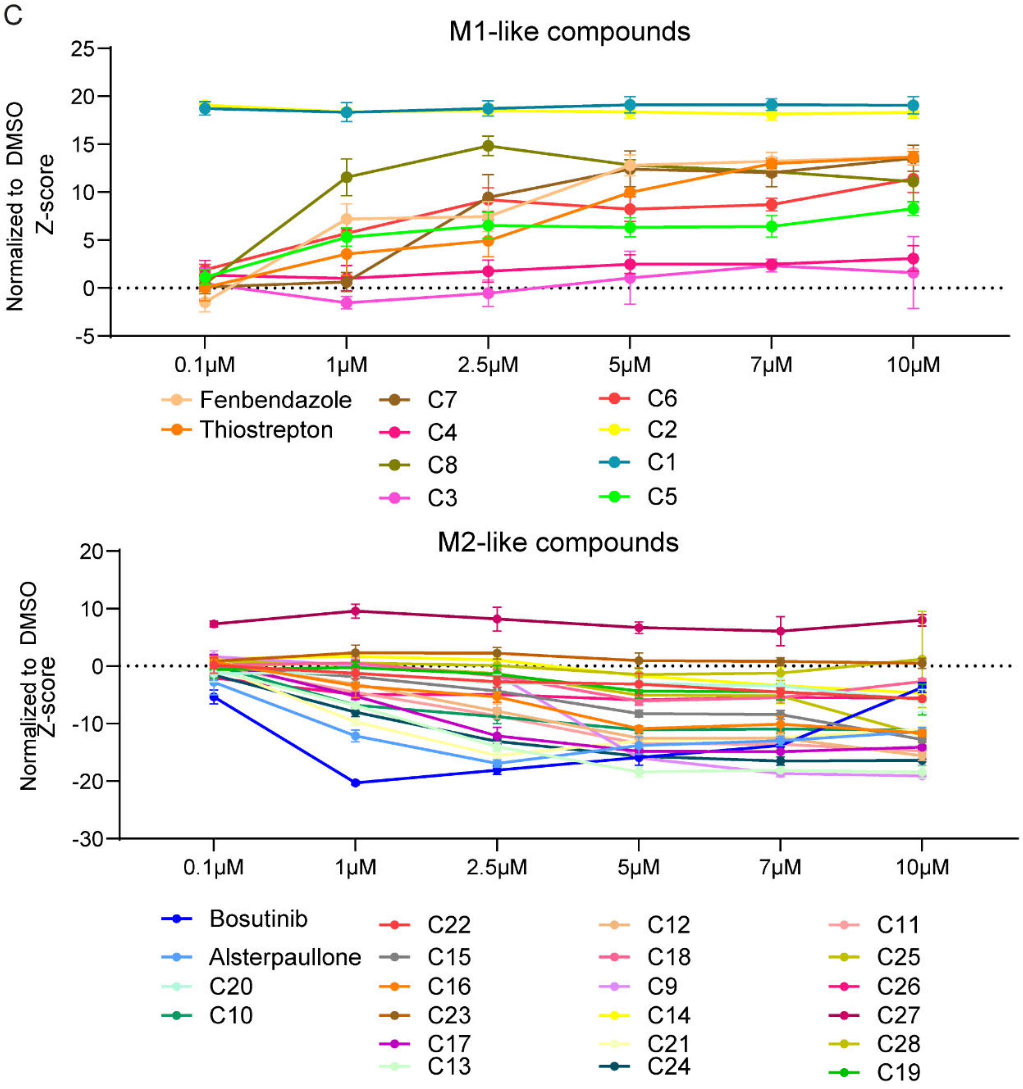
M1-like compounds
Normalized to DMSO Z-score
25
20
15
10
5
0
-5
0.1μM
1μM
2.5μM
5μM
7μM
10μM
Fenbendazole
Thiostrepton
C7
C4
C8
C3
C6
C2
C1
C5
M2-like compounds
Normalized to DMSO Z-score
20
10
0
-10
-20
-30
0.1μM
1μM
2.5μM
5μM
7μM
10μM
Bosutinib
Alsterpaullone
C20
C10
C22
C15
C16
C23
C17
C13
C12
C18
C9
C14
C21
C24
C11
C25
C26
C27
C28
C19

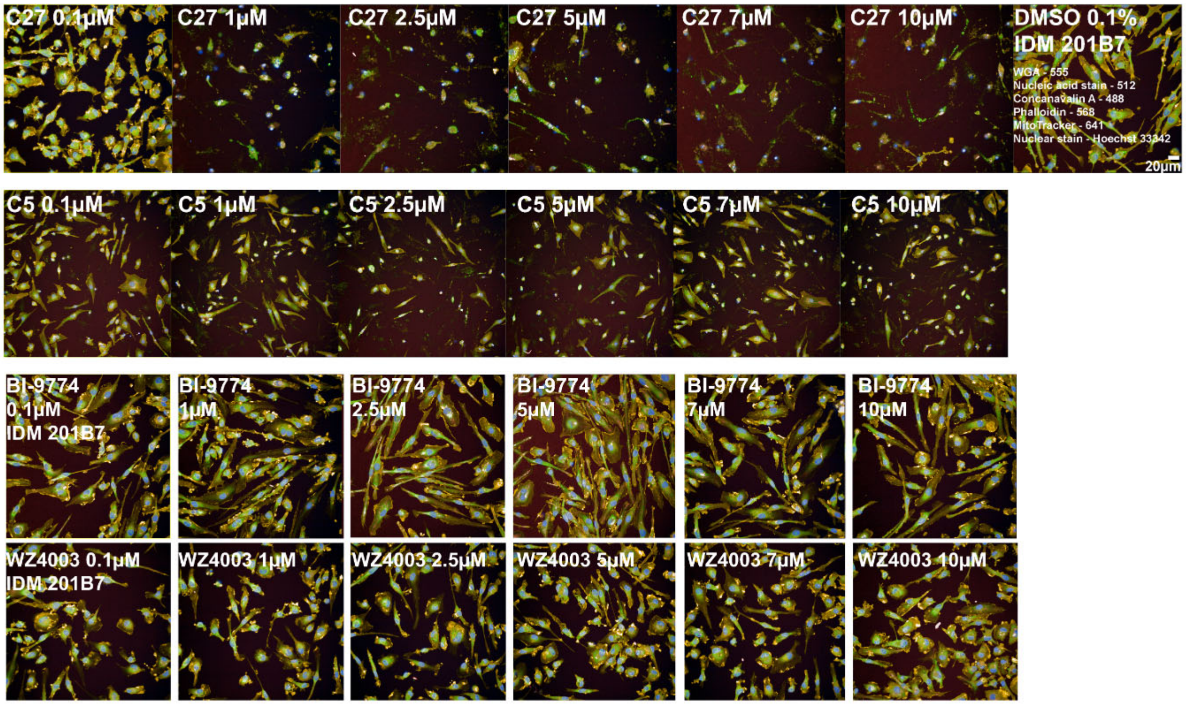
C27 0.1µM
C27 1µM
C27 2.5µM
C27 5µM
C27 7µM
C27 10µM
DMSO 0.1%
IDM 201B7
WGA - 555
Nucleic acid stain - 512
Concanavalin A - 488
Phalloidin - 568
MitoTracker - 641
Nuclear stain - Hoechst 33342
20µm
C5 0.1µM
C5 1µM
C5 2.5µM
C5 5µM
C5 7µM
C5 10µM
BI-9774 0.1µM IDM 201B7
BI-9774 1µM
BI-9774 2.5µM
BI-9774 5µM
BI-9774 7µM
BI-9774 10µM
WZ4003 0.1µM IDM 201B7
WZ4003 1µM
WZ4003 2.5µM
WZ4003 5µM
WZ4003 7µM
WZ4003 10µM

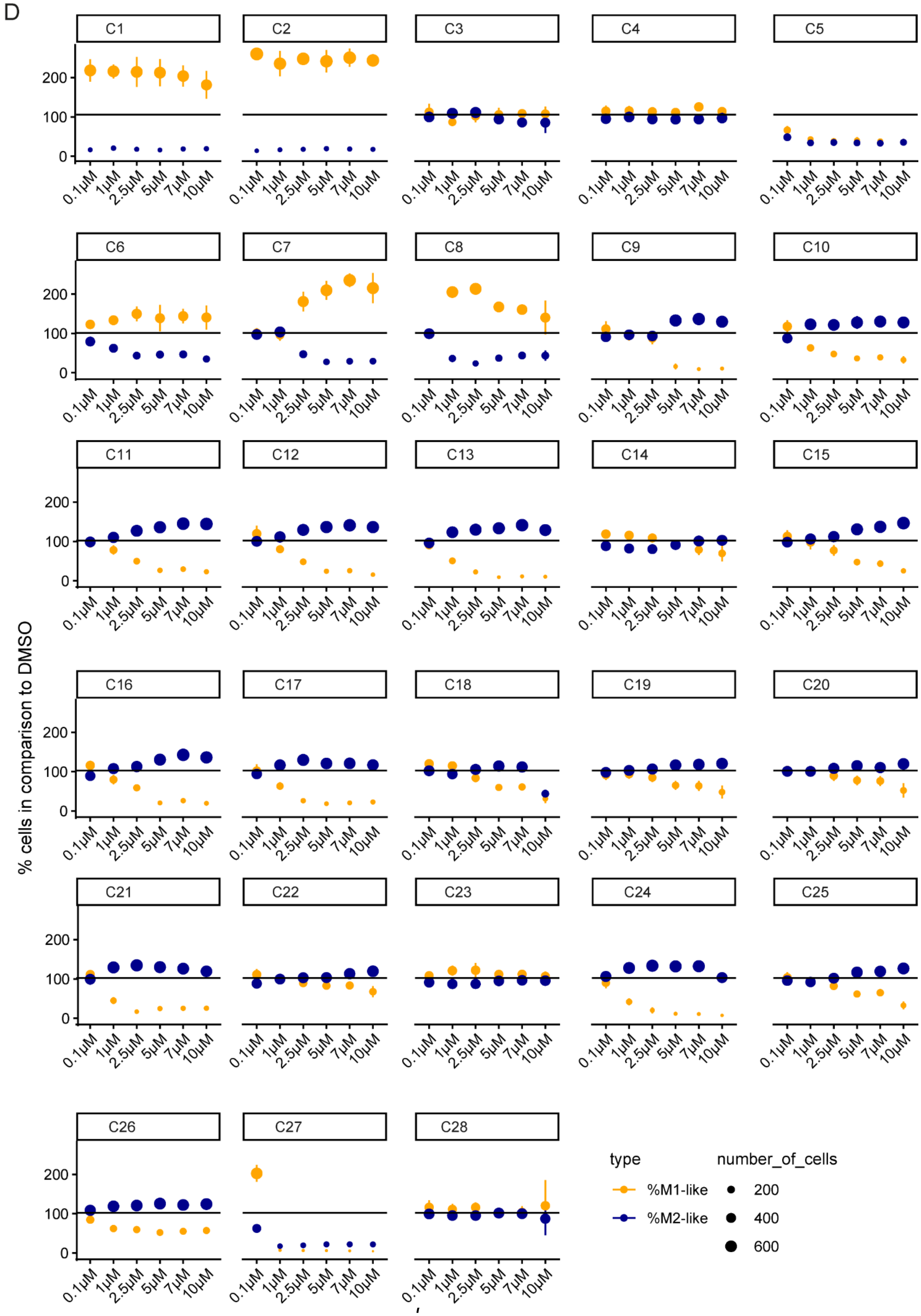
D
C1
C2
C3
C4
C5
C6
C7
C8
C9
C10
C11
C12
C13
C14
C15
C16
C17
C18
C19
C20
C21
C22
C23
C24
C25
C26
C27
C28
% cells in comparison to DMSO
0
100
200
0.1µM
1µM
2.5µM
5µM
7µM
10µM
type
%M1-like
%M2-like
number_of_cells
200
400
600

Figure S2. Dose-dependent cell roundness analysis and cell painting feature profiles of the confirmation screen hits. **(A)** Quantitative Z-score calculations for cell roundness analysis of the confirmation screen hits, categorized into M1- and M2-like cpds. DMSO-normalized Z-scores were determined from 20x magnified images and are depicted as mean ± SD for IDMs (201B7 line) and a representative MDM donor (CL414). Cells were treated with the indicated conditions (C = cpd; see Table S2) for 24 h. Hit selection thresholds were defined based on the internal M1- and M2-like control cpds (bosutinib 1 μM, alsterpaullone 1 and 10 μM, thiostrepton 2.5 μM, and fenbendazole 1 μM). Each library-cpd condition consisted of 4 wells per plate with 5 fields imaged per well (totaling n = 4 wells per condition). Other control conditions consisted of 12 wells per plate with 5 fields imaged per well (totaling n = 12 wells per condition). Statistical significance was determined for IDMs by a two-way ANOVA followed by Dunnett's multiple comparisons test against the vehicle control (DMSO 0.1%); asterisks denote statistical significance (****$p < 0.0001$, **$p < 0.01$, ns = non-significant). **(B)** Principal component analysis (PCA) of the confirmation screen hits determined by cell painting feature profiling. The profiling workflow utilized a customized cell painting image analysis pipeline combined with a linear classifier based on 1,279 extracted features. The hits WZ4003 (C4) and BI-9774 (C26) are highlighted alongside internal controls (DMSO, alsterpaullone 1 μM, thiostrepton 2.5 μM, bosutinib 1 μM, and fenbendazole 1 μM). DMSO-normalized %M2 − %M1 values represent the mean of 4 wells per condition (5 fields per well; 20x magnification) and are depicted for IDMs (201B7 line) and MDM donor CL414. Other control conditions consisted of 12 wells per plate with 5 fields imaged per well (totaling n = 12 wells per condition). The absolute cell count relative to the vehicle (DMSO) control is represented proportionally by the diameter of the data points. Treatment conditions correspond to those described in panel A. Exact DMSO-normalized %M2 − %M1 values for all cell models can be found in Table S4. **(C)** Dose-dependent Z-score kinetics and representative high-content imaging of confirmation screen hits. Dose-response curves illustrate DMSO-normalized Z-scores for cell roundness analysis (separated into M1- and M2-like cpds), depicted as mean ± SD for IDMs (201B7 line). Cells were treated for 24 h across a six-point dilution series (0.1 μM, 1 μM, 2.5 μM, 5 μM, 7 μM, and 10 μM). Each condition was imaged across two replicate 384-well plates (n = 4 wells total per condition). Representative high-content Opera Phenix™ confocal images (40x magnification; one representative field shown) illustrate cell-painted IDMs treated for 24 h with cpds C27, C5, C4 (WZ4003), and C26 (BI-9774) at the indicated concentrations to visualize cpd-induced phenotypic shifts and cytotoxicity. Multiplex staining was performed using PhenoVue™ Kit components (PhenoVue™ 641 Mitochondrial Stain, PhenoVue™ Hoechst 33342 Nuclear Stain, PhenoVue™ Fluor 488-Concanavalin A, PhenoVue™ 512 Nucleic Acid Stain, PhenoVue™ Fluor 555-WGA, and PhenoVue™ Fluor 568-Phalloidin). **(D)** Dose-dependent shifts in polarization state composition for individual hit cpds. Under the identical treatment conditions described in C, the absolute quantities of M1-like (yellow data points) and M2-like (blue data points) MΦs were computed based on the linear classifier %M2 − %M1 output values and total cell counts. Data are expressed as a percentage relative to the respective vehicle (DMSO 0.1%) control reference layout (data depicted as mean ± SD; n = 4 wells across two replicate plates). Data point diameters

proportionally represent the absolute cell count surviving the respective cpd treatment. Individual profiling plots are displayed systematically for all primary hits (C1–C28).

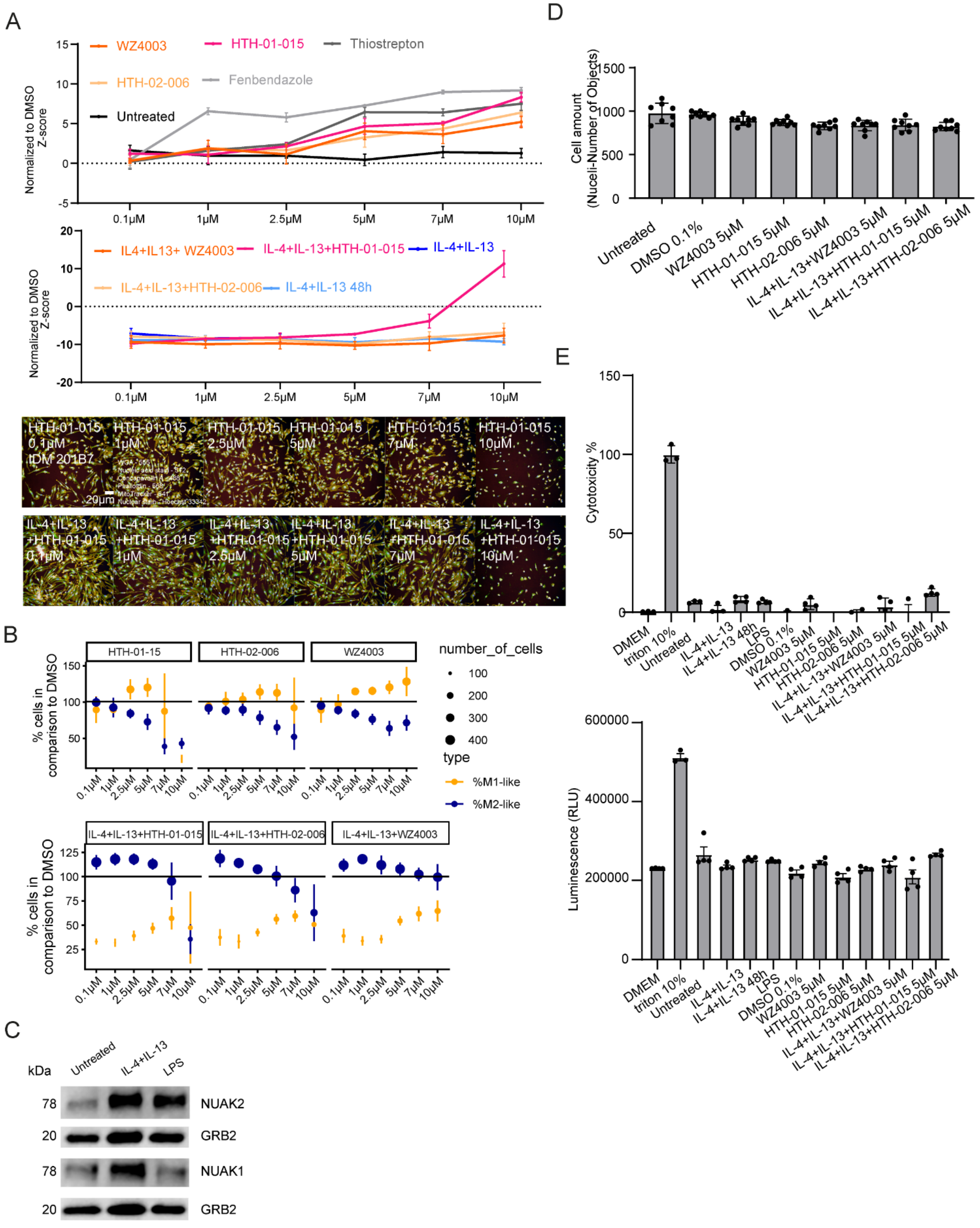
A
WZ4003
HTH-01-015
Thiostrepton
HTH-02-006
Fenbendazole
Untreated
Normalized to DMSO Z-score
0.1μM
1μM
2.5μM
5μM
7μM
10μM
IL4+IL13+ WZ4003
IL-4+IL-13+HTH-01-015
IL-4+IL-13
IL-4+IL-13+HTH-02-006
IL-4+IL-13 48h
HTH-01-015 0.1μM IDM 201B7
HTH-01-015 1μM
HTH-01-015 2.5μM
HTH-01-015 5μM
HTH-01-015 7μM
HTH-01-015 10μM
20μm
IL-4+IL-13 +HTH-01-015 0.1μM
IL-4+IL-13 +HTH-01-015 1μM
IL-4+IL-13 +HTH-01-015 2.5μM
IL-4+IL-13 +HTH-01-015 5μM
IL-4+IL-13 +HTH-01-015 7μM
IL-4+IL-13 +HTH-01-015 10μM
B
HTH-01-15
HTH-02-006
WZ4003
number_of_cells
100
200
300
400
type
%M1-like
%M2-like
% cells in comparison to DMSO
IL-4+IL-13+HTH-01-015
IL-4+IL-13+HTH-02-006
IL-4+IL-13+WZ4003
C
kDa
Untreated
IL-4+IL-13
LPS
78 NUAK2
20 GRB2
78 NUAK1
20 GRB2
D
Cell amount (Nuceli-Number of Objects)
Untreated
DMSO 0.1%
WZ4003 5μM
HTH-01-015 5μM
HTH-02-006 5μM
IL-4+IL-13+WZ4003 5μM
IL-4+IL-13+HTH-01-015 5μM
IL-4+IL-13+HTH-02-006 5μM
E
Cytotoxicity %
DMEM
triton 10%
Untreated
IL-4+IL-13
IL-4+IL-13 48h
LPS
DMSO 0.1%
WZ4003 5μM
HTH-01-015 5μM
HTH-02-006 5μM
IL-4+IL-13+WZ4003 5μM
IL-4+IL-13+HTH-01-015 5μM
IL-4+IL-13+HTH-02-006 5μM
Luminescence (RLU)

F

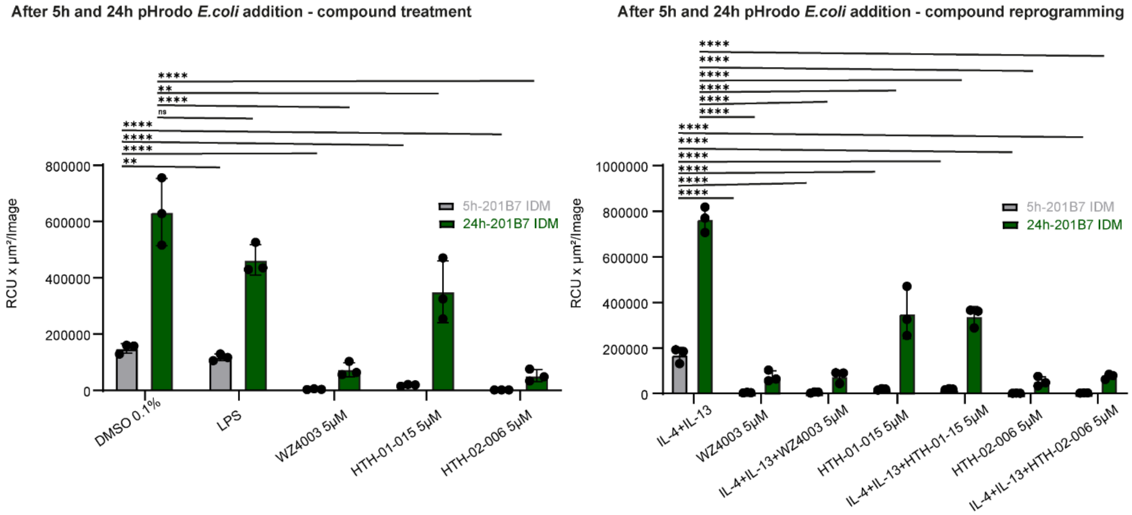
After 5h and 24h pHrodo E.coli addition - compound treatment
RCU x µm²/Image
5h-201B7 IDM
24h-201B7 IDM
DMSO 0.1%
LPS
WZ4003 5µM
HTH-01-015 5µM
HTH-02-006 5µM
After 5h and 24h pHrodo E.coli addition - compound reprogramming
IL-4+IL-13
IL-4+IL-13+WZ4003 5µM
IL-4+IL-13+HTH-01-15 5µM
IL-4+IL-13+HTH-02-006 5µM

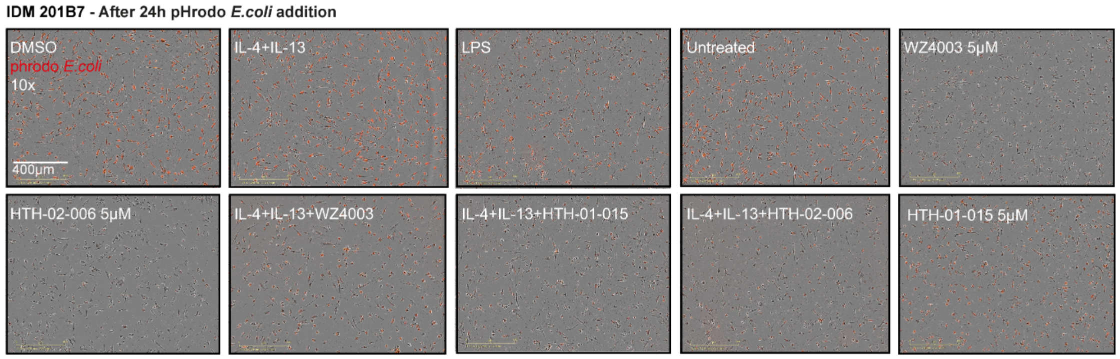
IDM 201B7 - After 24h pHrodo E.coli addition
DMSO
phrodo E.coli
10x
400µm
IL-4+IL-13
LPS
Untreated
WZ4003 5µM
HTH-02-006 5µM
IL-4+IL-13+WZ4003
IL-4+IL-13+HTH-01-015
IL-4+IL-13+HTH-02-006
HTH-01-015 5µM

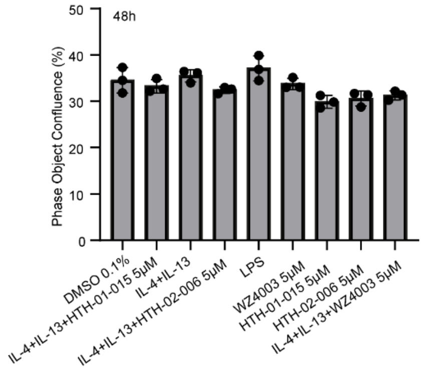
48h
Phase Object Confluence (%)
DMSO 0.1%
IL-4+IL-13+HTH-01-015 5µM
IL-4+IL-13
IL-4+IL-13+HTH-02-006 5µM
LPS
WZ4003 5µM
HTH-01-015 5µM
HTH-02-006 5µM
IL-4+IL-13+WZ4003 5µM

G

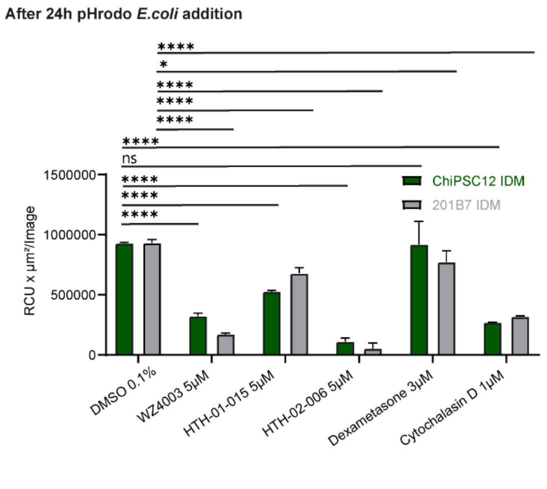
After 24h pHrodo E.coli addition
RCU x µm²/Image
ChiPSC12 IDM
201B7 IDM
DMSO 0.1%
WZ4003 5µM
HTH-01-015 5µM
HTH-02-006 5µM
Dexametasone 3µM
Cytochalasin D 1µM

H

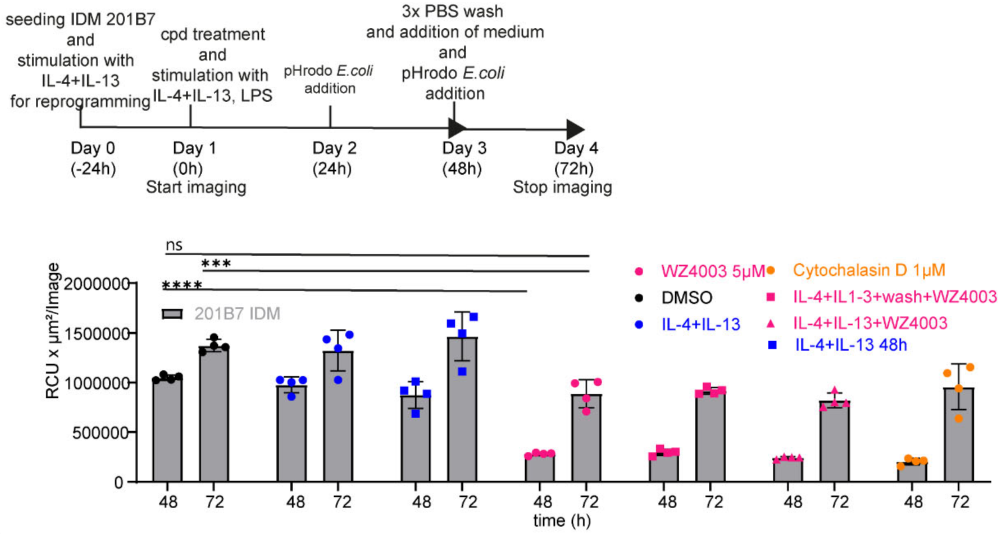
seeding IDM 201B7 and stimulation with IL-4+IL-13 for reprogramming
cpd treatment and stimulation with IL-4+IL-13, LPS
pHrodo E.coli addition
3x PBS wash and addition of medium and pHrodo E.coli addition
Day 0 (-24h)
Day 1 (0h) Start imaging
Day 2 (24h)
Day 3 (48h)
Day 4 (72h) Stop imaging
ns
***
****
201B7 IDM
WZ4003 5µM
DMSO
IL-4+IL-13
Cytochalasin D 1µM
IL-4+IL1-3+wash+WZ4003
IL-4+IL-13+WZ4003
IL-4+IL-13 48h
RCU x µm²/Image
2000000
1500000
1000000
500000
0
48
72
time (h)

I

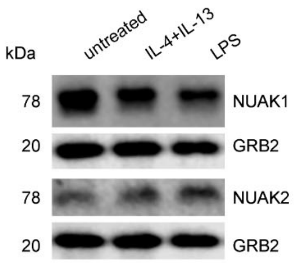
kDa
untreated
IL-4+IL-13
LPS
78
NUAK1
20
GRB2
78
NUAK2
20
GRB2

J

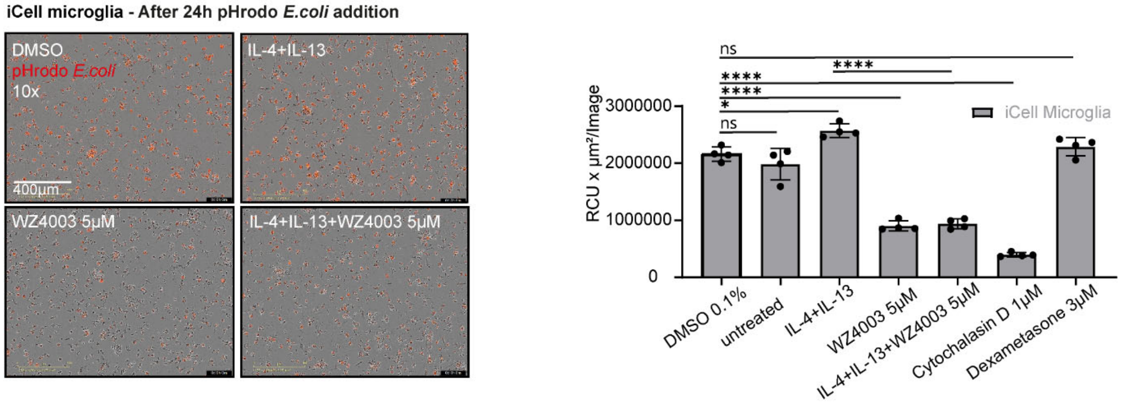
iCell microglia - After 24h pHrodo E.coli addition
DMSO
pHrodo E.coli
10x
400µm
IL-4+IL-13
WZ4003 5µM
IL-4+IL-13+WZ4003 5µM
ns
****
****
****
*
ns
iCell Microglia
RCU x µm²/Image
3000000
2000000
1000000
0
DMSO 0.1%
untreated
IL-4+IL-13
WZ4003 5µM
IL-4+IL-13+WZ4003 5µM
Cytochalasin D 1µM
Dexametasone 3µM

Figure S3. Effect of NUAK1/2 inhibition on cellular cytotoxicity, cell roundness and phagocytosis. **(A)** Dose-dependent Z-score calculation for cell roundness analysis of NUAK1/2i-treated IDMs. DMSO-normalized Z-scores were determined from 20x magnified images and are depicted as mean ± SD for IDMs (201B7 line). IDMs were treated with the indicated cpds (WZ4003, HTH-02-006, and HTH-01-015) for 24 h at six different concentrations (0.1 µM, 1 µM, 2.5 µM, 5 µM, 7 µM, and 10 µM). In the case of MΦ repolarization, IDMs were pre-treated for 24 h with IL-4+IL-13 (20 ng/mL), followed by a subsequent 24 h co-treatment with the respective cpds. Thiostrepton 2.5µM and fenbendazole 1µM served as internal M1-like control cpds. Each condition (4 wells per group, 5 fields imaged per well) was analyzed within a single 384-well plate. Representative high-content Opera Phenix™ confocal images (40x magnification; one representative field shown) illustrate cell-painted IDMs treated for 24 h with HTH-01-015 to visualize cpd-induced phenotypic shifts and cytotoxicity at elevated concentrations (10 µM). Multiplex staining was performed utilizing PhenoVue™ Kit components (PhenoVue™ 641 Mitochondrial Stain, PhenoVue™ Hoechst 33342 Nuclear Stain, PhenoVue™ Fluor 488-Concanavalin A, PhenoVue™ 512 Nucleic Acid Stain, PhenoVue™ Fluor 555-WGA, and PhenoVue™ Fluor 568-Phalloidin). **(B)** Quantification of polarization state composition shifts. Under the identical treatment conditions described in panel A, absolute quantities of M1-like and M2-like MΦs were computed based on the linear classifier %M2 − %M1 output values and overall cell numbers, expressed relative to the vehicle (DMSO 0.1%) control reference. **(C)** Immunoblotting of NUAK1 and NUAK2 expression in IDMs. IDMs were stimulated for 24 h with LPS (5 ng/mL) or IL-4+IL-13 (20 ng/mL). Whole-cell lysates were probed using specific antibodies against total NUAK1 and NUAK2, with GRB2 utilized as the internal loading control. **(D)** Absolute cell counts of NUAK1/2i-treated IDMs. Nuclei were stained with Hoechst 33342 to quantify total cell numbers per imaging area (mean ± SD; 5 fields per well, 8 wells per group within a single 384-well plate) under the treatment conditions described in panel A. **(E)** Lactate dehydrogenase (LDH) cytotoxicity assay of NUAK1/2i-treated IDMs. Total luminescence values (RLU, bottom plot) and corresponding Triton X-100 (10%)-normalized cytotoxicity percentages (top plot) are depicted as mean ± SD. LPS (5 ng/mL) and IL-4+IL-13 (20 ng/mL) stimulations served as additional biological controls. **(F)** Kinetic analysis of *E.coli* phagocytosis in NUAK1/2i-treated IDMs. Target cells were treated as described in panel D. Quantitative data represent total red object integrated intensity (RCU × µm²/image) reflecting the engulfment of pHrodo Red-labeled *E.coli* particles, monitored using an IncuCyte® SX3 live-cell imaging system (mean ± SEM; data points shown at 5 h and 24 h post-addition; 10x magnification). Representative live-cell overlay images (10x magnification; 24 h endpoint) illustrate particle engulfment across the indicated conditions. Baseline cell density was verified via phase object confluence percentages at the assay endpoint. Statistical analysis was performed using a two-way ANOVA followed by Dunnett's post-hoc test against the vehicle control; asterisks denote statistical significance (****$p < 0.0001$, **$p < 0.01$, ns = non-significant). **(G)** Comparative phagocytosis analysis in alternative iPSC lines. Human IDMs generated from the 201B7 and ChiPSC12 lines were evaluated as described in panel E. Cytochalasin D (1 µM, 1 h pre-treatment) and dexamethasone (3 µM, 24 h treatment) served as internal negative and positive

controls for particle engulfment, respectively. Quantitative data represent mean ± SEM monitored at 5 h and 24 h using the IncuCyte® SX3 system. Statistical analysis was performed using a two-way ANOVA followed by Tukey's multiple comparisons test; asterisks denote significance (****$p < 0.0001$, *$p < 0.05$, ns = non-significant). **(H)** Phagocytic recovery analysis following cpd wash-out. IDMs (201B7 line) were treated as outlined in panel F. For MΦ repolarization tracking, cells were treated for 24 h with IL-4+IL-13 (20 ng/mL) followed by 24 h of WZ4003 (5 µM) exposure, or subjected to three subsequent PBS washes following the 24 h cpd treatment (designated as "wash") to allow metabolic recovery. Following an initial 24 h kinetic monitoring of *E.coli* engulfment, cells were washed three times with PBS and re-incubated with fresh *E.coli* particles in cpd-free medium for an additional 24 h. Cytochalasin D (1 µM) and dexamethasone (3 µM) served as internal negative and positive controls. Quantitative data represent mean ± SEM. Statistical significance was resolved via a two-way ANOVA followed by Tukey's post-hoc test (****$p < 0.0001$, *$p < 0.05$). **(I)** Immunoblotting of NUAK1 and NUAK2 expression in iPSC-derived microglia. Human iPSC-derived microglia (iCell microglia) were stimulated for 24 h with LPS (5 ng/mL) or IL-4+IL-13 (20 ng/mL). Whole-cell lysates were resolved and probed for total NUAK1 and NUAK2, with GRB2 serving as the loading control. **(J)** Quantitative phagocytosis analysis in WZ4003-treated iCell microglia. Human iPSC-derived microglia were treated with WZ4003 (5 µM) for 24 h. For microglia repolarization, cells were pre-treated with IL-4+IL-13 (20 ng/mL) for 24 h followed by a 24 h co-incubation with WZ4003 (5 µM). Cytochalasin D (1 µM) and dexamethasone (3 µM) served as negative and positive controls. Data represent total red object integrated intensity (mean ± SEM; 24 h time point; 10x magnification) accompanied by representative live-cell overlay images. Statistical analysis was performed using a one-way ANOVA followed by Dunnett's multiple comparisons test against the vehicle control (****$p < 0.0001$, *$p < 0.05$, ns = non-significant).

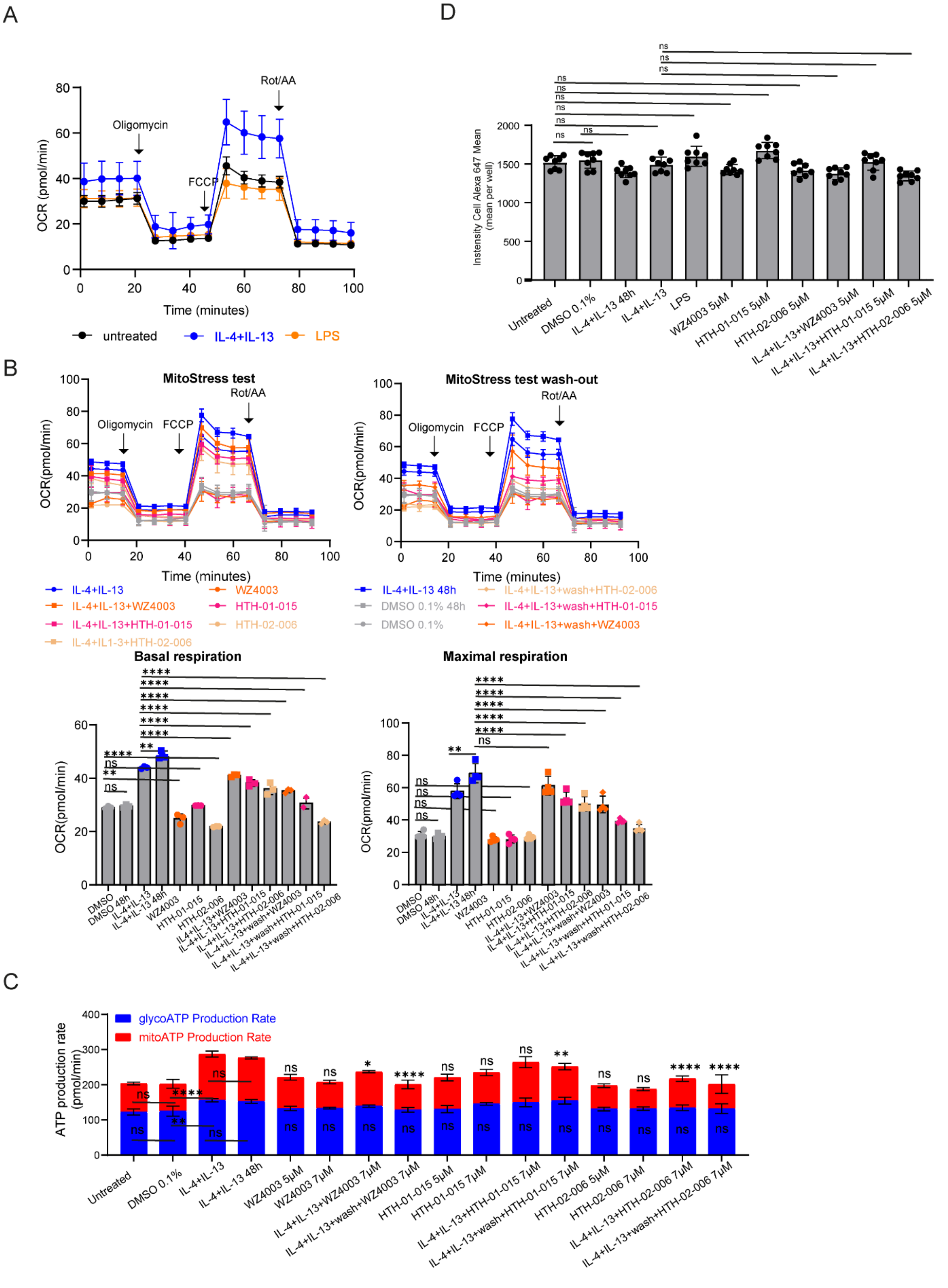
A
Oligomycin
FCCP
Rot/AA
OCR (pmol/min)
Time (minutes)
untreated
IL-4+IL-13
LPS
D
Instensity Cell Alexa 647 Mean (mean per well)
Untreated
DMSO 0.1%
IL-4+IL-13 48h
IL-4+IL-13
LPS
WZ4003 5μM
HTH-01-015 5μM
HTH-02-006 5μM
IL-4+IL-13+WZ4003 5μM
IL-4+IL-13+HTH-01-015 5μM
IL-4+IL-13+HTH-02-006 5μM
B
MitoStress test
MitoStress test wash-out
OCR(pmol/min)
Time (minutes)
IL-4+IL-13
WZ4003
IL-4+IL-13+WZ4003
HTH-01-015
IL-4+IL-13+HTH-01-015
HTH-02-006
IL-4+IL1-3+HTH-02-006
IL-4+IL-13 48h
DMSO 0.1% 48h
DMSO 0.1%
IL-4+IL-13+wash+HTH-02-006
IL-4+IL-13+wash+HTH-01-015
IL-4+IL-13+wash+WZ4003
Basal respiration
Maximal respiration
C
glycoATP Production Rate
mitoATP Production Rate
ATP production rate (pmol/min)
WZ4003 7μM
IL-4+IL-13+WZ4003 7μM
IL-4+IL-13+wash+WZ4003 7μM
HTH-01-015 7μM
IL-4+IL-13+HTH-01-015 7μM
IL-4+IL-13+wash+HTH-01-015 7μM
HTH-02-006 7μM
IL-4+IL-13+HTH-02-006 7μM
IL-4+IL-13+wash+HTH-02-006 7μM

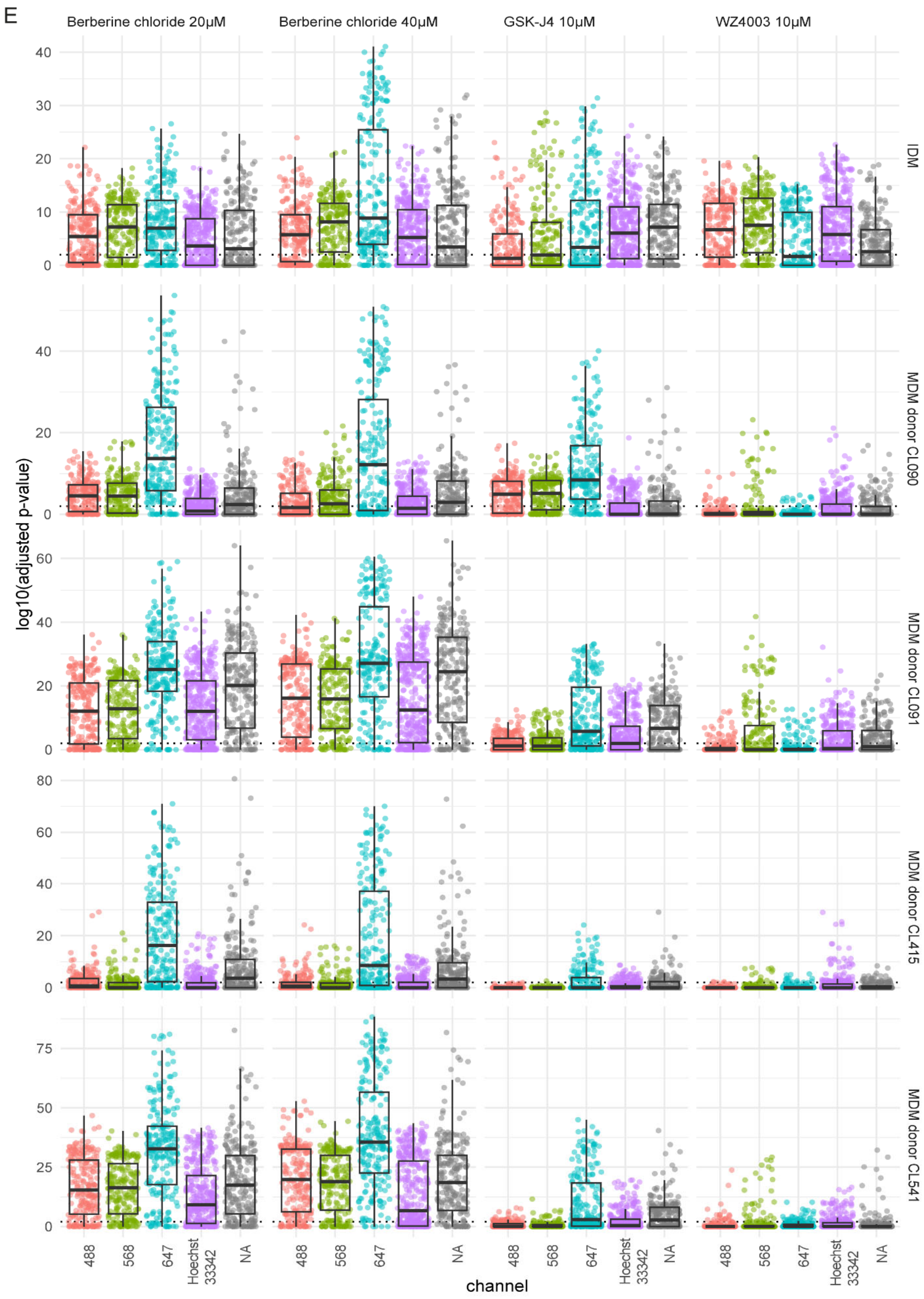
E
Berberine chloride 20µM
Berberine chloride 40µM
GSK-J4 10µM
WZ4003 10µM
IDM
MDM donor CL090
MDM donor CL091
MDM donor CL415
MDM donor CL541
log10(adjusted p-value)
channel
488
568
647
Hoechst 33342
NA

F

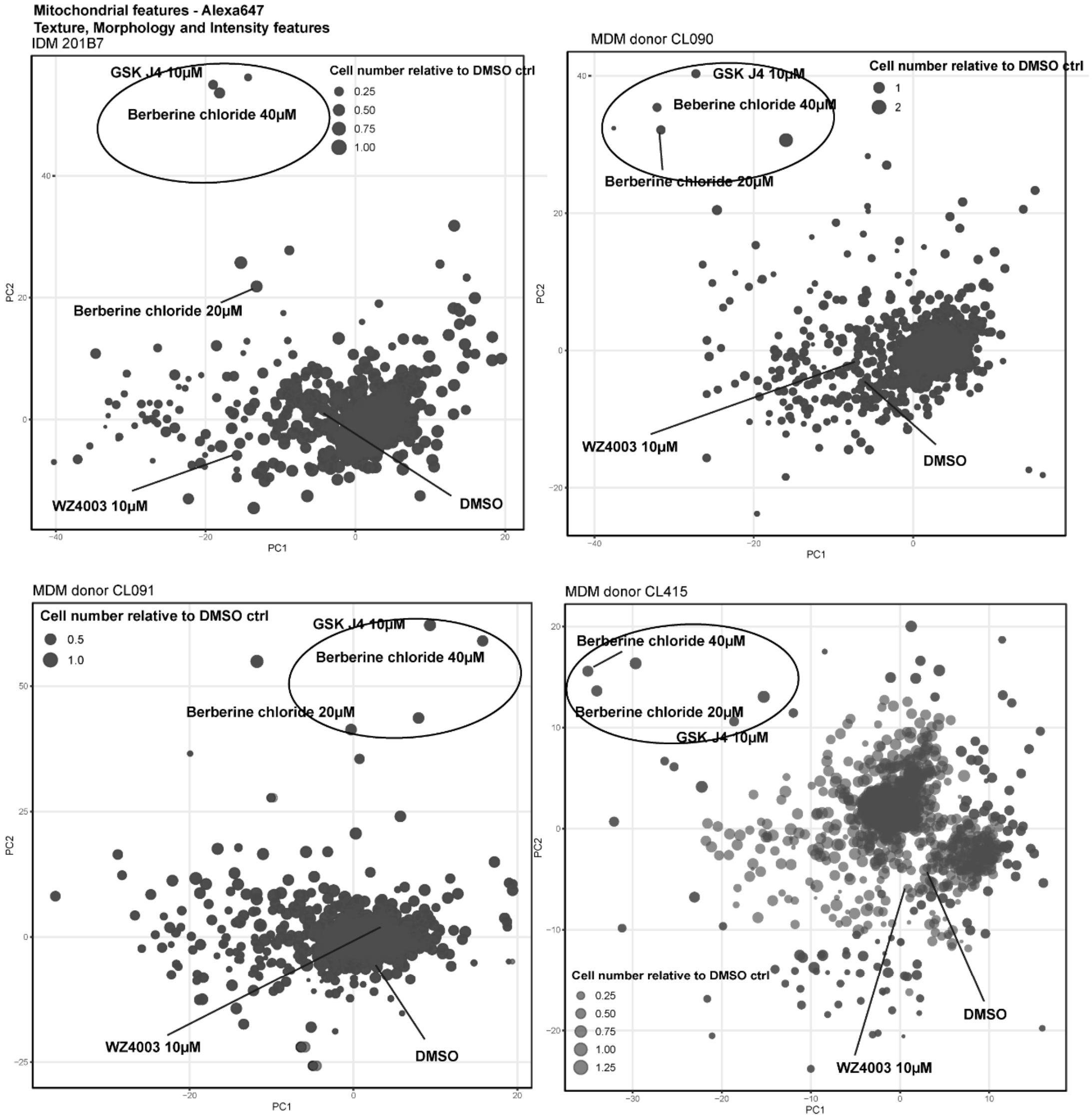
Mitochondrial features - Alexa647
Texture, Morphology and Intensity features
IDM 201B7
GSK J4 10µM
Berberine chloride 40µM
Cell number relative to DMSO ctrl
0.25
0.50
0.75
1.00
Berberine chloride 20µM
WZ4003 10µM
DMSO
PC1
PC2
MDM donor CL090
GSK J4 10µM
Berberine chloride 40µM
Berberine chloride 20µM
Cell number relative to DMSO ctrl
1
2
WZ4003 10µM
DMSO
MDM donor CL091
Cell number relative to DMSO ctrl
0.5
1.0
GSK J4 10µM
Berberine chloride 40µM
Berberine chloride 20µM
WZ4003 10µM
DMSO
MDM donor CL415
Berberine chloride 40µM
Berberine chloride 20µM
GSK J4 10µM
Cell number relative to DMSO ctrl
0.25
0.50
0.75
1.00
1.25
DMSO
WZ4003 10µM

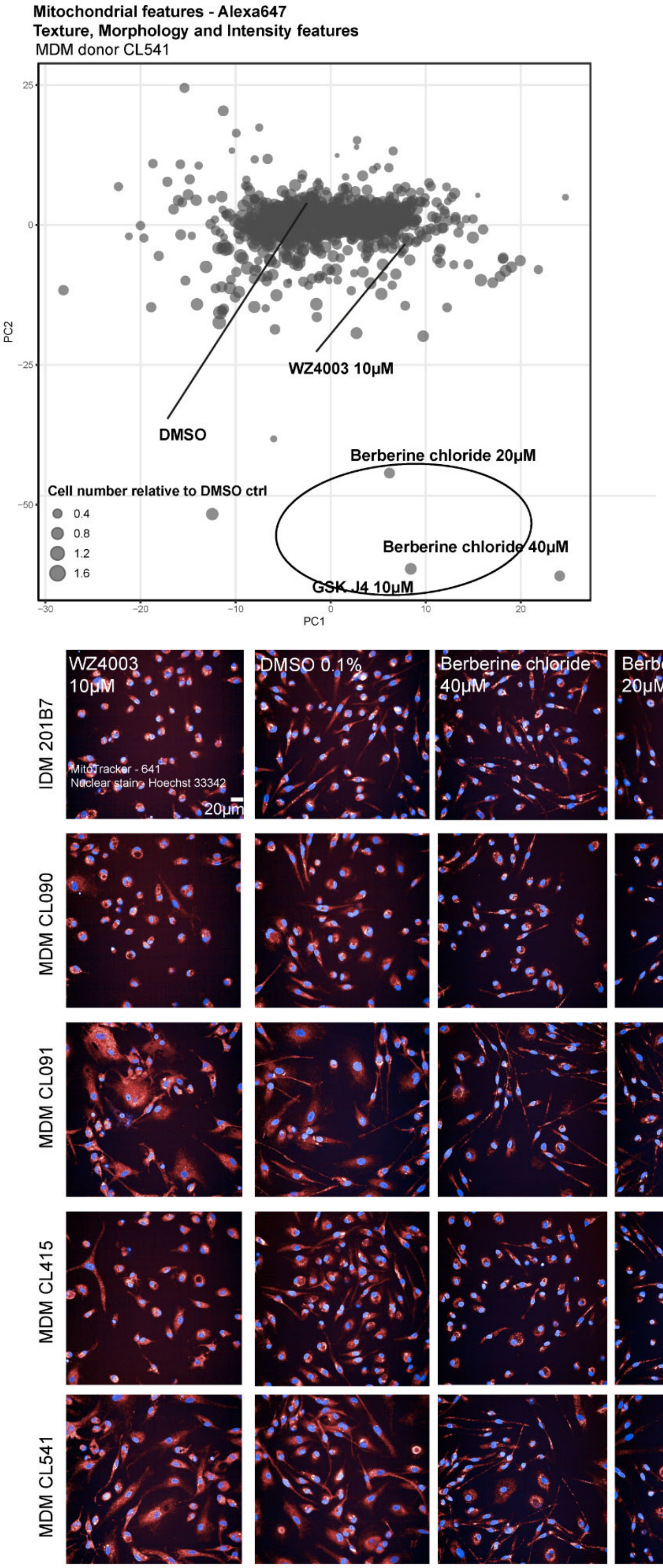
Mitochondrial features - Alexa647
Texture, Morphology and Intensity features
MDM donor CL541
PC2
PC1
WZ4003 10µM
DMSO
Berberine chloride 20µM
Berberine chloride 40µM
GSK J4 10µM
Cell number relative to DMSO ctrl
0.4
0.8
1.2
1.6
WZ4003 10µM
DMSO 0.1%
Berberine chloride 40µM
Berberine chloride 20µM
GSK J4 10µM
IDM 201B7
MDM CL090
MDM CL091
MDM CL415
MDM CL541
MitoTracker - 641
Nuclear stain - Hoechst 33342
20µm

Identification of a NUAK1/2 inhibitor as a macrophage-polarizing compound by a machine learning-based phenotypic cell painting screen

G

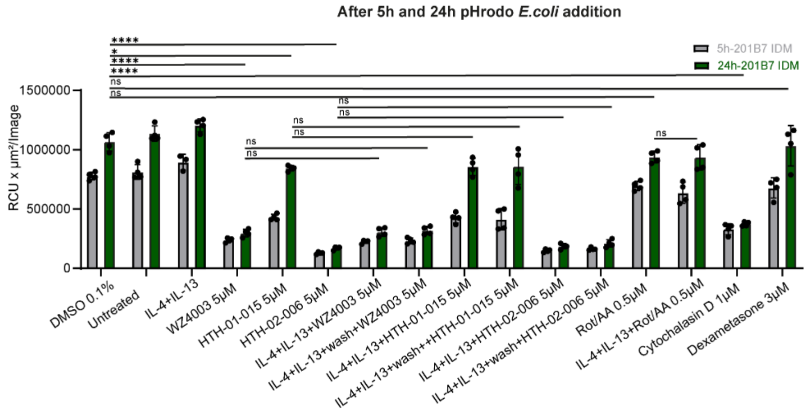

H

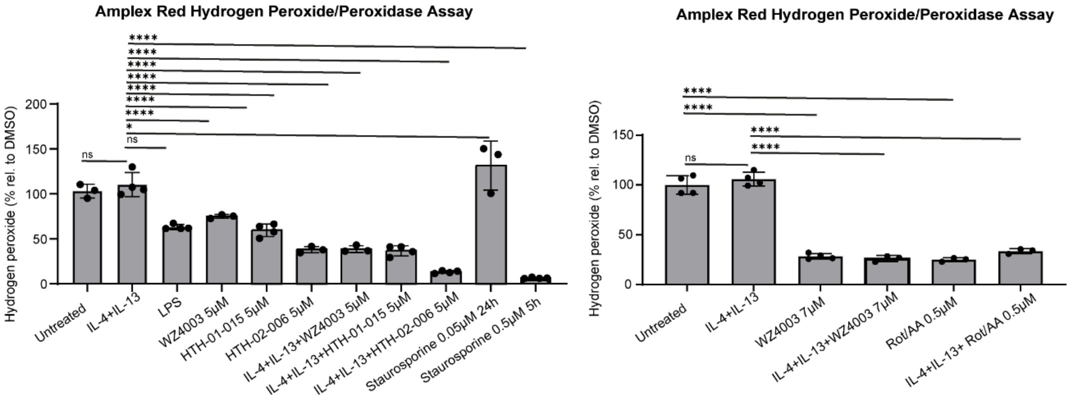

I

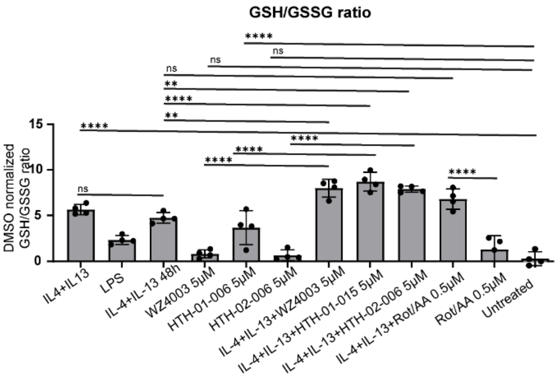

Figure S4. Effect of NUAK1/2 inhibition on mitochondrial function, feature profiles, and cellular redox status. **(A)** MitoStress assay of IDMs treated with WZ4003. IDMs (201B7 line) were treated for 24 h with the indicated conditions (DMSO 0.1%, untreated, LPS 5 ng/mL, IL-4+IL-13 20 ng/mL, and WZ4003 5 μM). For MΦ repolarization, cells were pre-treated for 24 h with IL-4+IL-13 (20 ng/mL), followed by a subsequent 24 h co-treatment with WZ4003. Oxygen consumption rates (OCR; pmol/min) are depicted as mean ± SEM ($n = 4$) replicates per condition) during sequential injections of 1.5 μM oligomycin, 2 μM FCCP, and 0.5 μM Rot/AA (4 measurement cycles per injection block). **(B)** MitoStress assay of multi-cpd NUAK1/2-inhibited IDMs. IDMs were treated for 24 h with the indicated conditions (DMSO 0.1%, IL-4+IL-13 20 ng/mL, WZ4003 7 μM, HTH-02-006 7 μM, and HTH-01-015 7 μM). For MΦ repolarization, cells were treated for 24 h with IL-4+IL-13 (20 ng/mL), followed by a 24 h cpd exposure or a cpd-free recovery phase achieved via three consecutive PBS washes following a 24 h cpd treatment (designated as "wash"). OCR metabolic curves and corresponding basal/maximal respiration bar charts represent mean ± SEM ($n = 4$) replicates). Statistical analysis was performed using a one-way ANOVA followed by Dunnett's post-hoc test against the vehicle control; asterisks denote significance (****$p < 0.0001$, **$p < 0.01$, ns = non-significant). **(C)** ATP production rates of NUAK1/2i-treated IDMs. Quantitative glycolytic ATP production rates (glycoATP, blue bars) and mitochondrial ATP production rates (mitoATP, red bars) are depicted as mean ± SEM ($n = 4$) replicates per condition) under the treatment layouts described in panel B using concentrations of 5 μM and 7 μM for the respective cpds. Statistical significance was resolved via a one-way ANOVA with Dunnett's multiple comparisons test (****$p < 0.0001$, **$p < 0.01$, *$p < 0.05$, ns = non-significant). **(D)** Mitochondrial staining intensity metrics in phenotypically modulated IDMs. IDMs were treated for 24 h as indicated. Relative mitochondrial signal abundance was computed as the well-normalized background-subtracted mean intracellular fluorescence intensity in the Alexa Fluor 647 mitochondrial channel derived from high-content cell-painted images (data depicted as mean ± SD; ($n = 8$) wells). Statistical analysis was determined via a one-way ANOVA (ns = non-significant). **(E)** Distribution profiles of feature variations. Normalized feature profiles from the screening conditions described in panel F were evaluated against the vehicle control (DMSO 0.1%) using an unpaired t-test. The landscape distribution of resulting Bonferroni-adjusted p-values is displayed, demonstrating compartment-specific variability profiles across different multiplex dyes. **(F)** Principal component analysis (PCA) of primary screen hits utilizing targeted mitochondrial features. PCA profiling along the PC1 and PC2 axes was executed utilizing a customized cell painting mitochondrial image analysis pipeline tracking 222 dedicated texture, morphology, and intensity features. Profiles are mapped across independent MΦ models (IDM 201B7 line and MDM donors CL090, CL091, CL415, and CL541) treated with DMSO 0.1%, WZ4003 10 μM, GSK-J4 10 μM, and berberine chloride (20 and 40 μM). Data point diameters proportionally represent absolute cell counts relative to the vehicle control ($n = 4$) wells across two replicate plates per condition; ($n = 12$) wells for berberine chloride and vehicle benchmarks). Representative high-content Opera Phenix™ confocal images (40x magnification) illustrate multiplexed MΦs showing the mitochondrial (PhenoVue™ 641) and nuclear (PhenoVue™ Hoechst 33342) channels. **(G)** Kinetic phagocytosis tracking after extended

E.coli challenge. IDMs were treated for 24 h as indicated. Cytochalasin D (1 μM, 1 h pre-treatment) and dexamethasone (3 μM, 24 h treatment) served as internal negative and positive controls for particle engulfment, respectively. Total red object integrated intensity (RCU × μm^2/image) reflecting pHrodo Red-labeled *E.coli* engulfment was monitored in the IncuCyte® SX3 system, represented at the 5 h and 24 h challenge time points (data depicted as mean ± SEM; 10x magnification). Statistical significance was resolved via a two-way ANOVA followed by Tukey's post-hoc test (****p < 0.0001, *p < 0.05, ns = non-significant). **(H)** Total cytosolic ROS accumulation in NUAK1/2i- and Rot/AA-treated IDMs. IDMs were incubated for 24 h under the indicated conditions. Cytosolic hydrogen peroxide levels are expressed as a percentage relative to the vehicle control (data depicted as mean ± SD; (*n* = 4) replicates), utilizing staurosporine (0.05 μM) as an internal positive control for cell stress. Statistical analysis was performed using a one-way ANOVA followed by Dunnett's multiple comparisons test (****p < 0.0001, *p < 0.05, ns = non-significant). **(I)** Total glutathione status tracking in NUAK1/2i-treated IDMs. Intracellular reduced-to-oxidized glutathione ratios (GSH/GSSG) were determined under the cpd layout blocks described in panel B. Normalized ratios relative to the vehicle control are expressed as mean ± SD (*n* = 4) biological replicates). Significance thresholds were evaluated via a one-way ANOVA followed by Dunnett's multiple comparisons test against the vehicle control (****p < 0.0001, **p < 0.01, ns = non-significant).

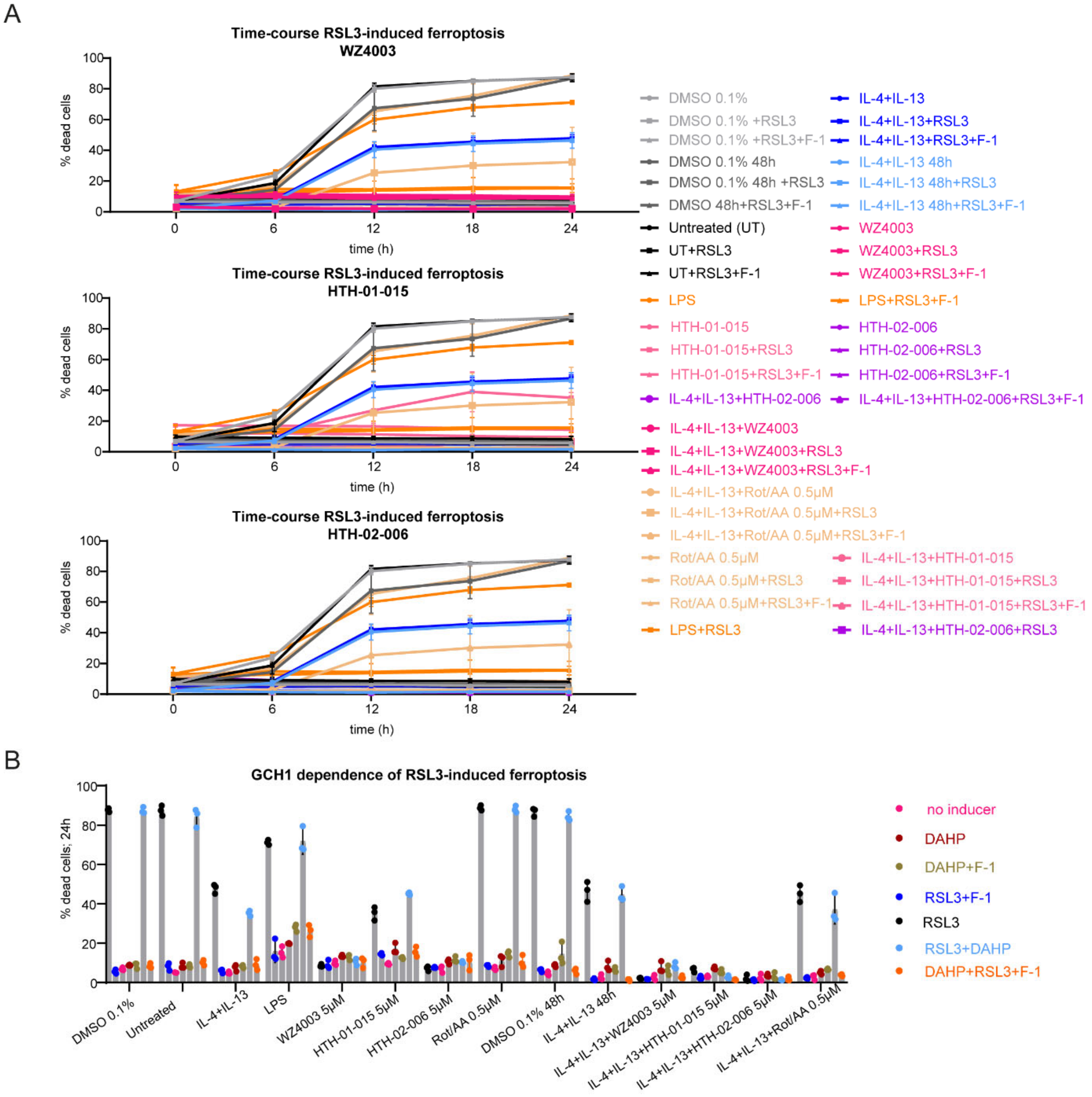

Figure S5. Effect of NUAK1/2 inhibition and GCH1 blockade on RSL3-induced ferroptosis. **(A)** Time-course analysis of RSL3-induced ferroptosis in NUAK1/2i-treated IDMs. IDMs (201B7 line) were treated with the indicated cpds (WZ4003 5 µM, HTH-01-015 5 µM, HTH-02-006 5 µM, and Rot/AA 0.5 µM) or biological stimuli (LPS 5 ng/mL and IL-4+IL-13 20 ng/mL) for 24 h or 48 h. For MΦ repolarization, IDMs were pre-treated for 24 h with IL-4+IL-13 (20 ng/mL), followed by a subsequent 24 h co-treatment with the respective cpds. To induce and modulate ferroptosis, RSL3 (0.1 µM) and the ferroptosis inhibitor ferrostatin-1 (F-1, 1 µM) were added as indicated. Kinetic monitoring of cell death (0 h, 6 h, 12 h, 18 h, and 24 h post-challenge) was performed across all treatment conditions, reading out the percentage of dead cells via cell-impermeant CellTox™ Green dye fluorescence using an IncuCyte® SX3 live-cell imaging system (data depicted as mean ± SD; imaging executed every 6 h; 10x magnification; 2 fields imaged per well; *n* = 3 replicates per condition). **(B)** Evaluation of GCH1 involvement in RSL3-induced ferroptosis within NUAK1/2i-treated IDMs. Cpd treatment, cell repolarization, and phenotypic

monitoring were performed identically to the parameters described in panel A, with data displayed strictly for the 24 h post-challenge endpoint tracking layout (data depicted as mean ± SD; *n* = 3 replicates). To pharmacologically block GCH1 activity, 4 mM DAHP (2,4-Diamino-6-hydroxypyrimidine) was added in the presence or absence of RSL3 and F-1 cotreatments. Statistical analysis was performed utilizing a two-way ANOVA followed by Tukey's multiple comparisons test to resolve significance thresholds between target treatment groups and corresponding vehicle or pathway control blocks (ns = non-significant).

Supplementary Tables

The supplementary tables (Table S1-S5) can be downloaded at:
https://www.mdpi.com/article/10.3390/ijms27167279/s1.
